# BREATHER Plus clinical trial design: A randomised non-inferiority trial evaluating the efficacy, safety and acceptability of short cycle (five days on, two days off) dolutegravir/tenofovir-based triple antiretroviral therapy (ART) compared to daily ART in virologically suppressed adolescents living with HIV aged 12 to <20 years in sub-Saharan Africa☆

**DOI:** 10.1016/j.cct.2025.107963

**Published:** 2025-05-29

**Authors:** Fredrick Katongole, Tiyara Arumugam, Angus Jennings, Constantine Mutata, Patrick Ssebunya, Charity Wamboi, Alexandra Green, Mutsa Bwakura-Dangarembizi, Cissy Kityo, Abraham Siika, Moherndran Archary, Lungile Jafta, Stella Namukwaya, Janet Seeley, Henry Mugerwa, Simon Walker, Naomi Apoto, Margaret J. Thomason, Deborah Ford, Sarah L. Pett, Adeodata R. Kekitiinwa

**Affiliations:** a Baylor College of Medicine Children’s Foundation, Kampala, Uganda; b Department of Paediatrics and Children Health, King Edward VIII Hospital, Enhancing Care Foundation, University of KwaZulu-Natal, Durban, South Africa; c Medical Research Council Clinical Trials Unit at University College London, United Kingdom; d University of Zimbabwe Clinical Research Centre, Harare, Zimbabwe; e Joint Clinical Research Centre, Kampala, Uganda; f Moi University Clinical Research Centre, Eldoret, Kenya; g Fondazione Penta ETS, Padova, Italy; h Medical Research Council/ Uganda Virus Research Institute and London School of Hygiene and Tropical Medicine Uganda Research Unit, Entebbe, Uganda; i Centre for Health Economics, University of York, United Kingdom

**Keywords:** Clinical trial, HIV, Adolescents, Short-cycle, Antiretroviral, Dolutegravir

## Abstract

**Background:**

Novel strategies to improve ART adherence, retention in care and quality of life among adolescents living with HIV (ALHIV) are needed. Short-Cycle Therapy (SCT) with 4/5 sequential days on ART, 2/3 days off ART per week has shown non-inferior virological outcomes and high acceptability, but most data are in adults and are very limited for dolutegravir (DTG)-based SCT.

**Methods:**

BREATHER Plus is an ongoing 96-week non-inferiority randomised trial evaluating efficacy, safety and acceptability of SCT (5 sequential days on, 2 days off at the weekend) with DTG/tenofovir (TNV)-based triple ART versus continuous (daily) therapy (CT) in ALHIV. Participants are aged 12 to <20 years in Kenya/South Africa/Uganda/Zimbabwe, virologically suppressed (Viral Load (VL) <50copies/mL) for ≥12 months at enrollment, with no prior treatment failure. Randomisation is 1:1 to SCT versus CT. VL monitoring for clinical management is 6–12 monthly aligning with standard-of-care. The primary outcome is confirmed virological rebound ≥50 copies/mL by 96 weeks. The trial employs the Smooth Away From Expected (SAFE) non-inferiority frontier, where the non-inferiority margin depends on the observed event risk in the CT arm. Secondary outcomes include HIV resistance, toxicities, patient-reported outcomes and cost-effectiveness. Enrolment of 470 participants completed in June 2023.

**Discussion:**

BREATHER Plus is the first randomised trial specifically evaluating DTG/TNV-triple based SCT. Rapid roll-out of DTG and a pragmatic approach to VL monitoring mean results will be generalisable to ALHIV across sub-Saharan Africa. If SCT provides non-inferior virological suppression to CT, it may offer choice for ALHIV on how they take their ART.

## 1 Background

Globally in 2023, an estimated 1.5 million adolescents (10–19 years) were living with HIV (ALHIV), with close to 90 % in sub-Saharan Africa [1,2]. Adolescents continue to have poorer HIV treatment outcomes [3–5] including lower treatment adherence, higher loss to follow-up, poorer virologic suppression and higher mortality than older people living with HIV.

There has been little research into strategies aimed at improving retention in care and ART adherence in ALHIV in SSA. Short cycle therapy (SCT) incorporates regular breaks in the standard-of-care daily ART during each week, compared to continuous (daily) therapy (CT) regimens. The aims of SCT are to reduce the burden of daily pill taking, whilst maintaining virological suppression. Typically, no tablets are taken for 2 or 3 consecutive days over the weekend period, although strategies with extended periods off-ART of up to 7 days have been considered. As a strategy, SCT may appeal especially to young people, allowing them to have weekends off treatment. The BREATHER trial [6] is the only randomised trial, which has evaluated SCT in children and young people. BREATHER enrolled 199 participants aged 8–24 years (70 in Uganda) and showed non-inferiority of SCT (5 days on, 2 days off) using efavirenz-based ART versus continuous therapy (CT) with respect to virological suppression over 48 and 144 weeks (Table 1); young people expressed strong preference for SCT [6,7]. The only trial to assess SCT on DTG-based ART is the ANRS-QUATUOR 170 study (*n* = 636) [8], a France-wide RCT among predominantly middle-aged males, in which just under half were on integrase-inhibitor (INSTI) based regimens (mostly dolutegravir). ANRS-QUATUOR 170 demonstrated non-inferiority of SCT (4 days on, 3 days off) versus daily ART based on virologic suppression <50c/mL at 48 weeks, with no difference in the intervention effects between ART regimens; extended follow-up to 96 weeks resulted in very few failures on INSTI-based ART (Table 1). Other studies include a small Ugandan trial in adults (*n* = 57) [9], which showed non-inferiority of 5 days on, 2 days off versus CT, mostly efavirenz-based ART (*n* = 56), using a composite treatment failure outcome over 72 weeks (Table 1). Notably, all SCT studies except one small proof-of-concept trial [10] have included viral load (VL) monitoring at least every 12 weeks, so results cannot be generalized to low- and middle-income countries with less frequent VL monitoring.

The World Health Organization (WHO) guidelines now recommend DTG-based regimens (DTG+ 2 nucleos(*t*)ide reverse transcriptase inhibitor (NRTI)) first-line [11] and countries in sub-Saharan Africa have rapidly transitioned young people to DTG. DTG has pharmacological attributes such as a long intracellular half-life and a high genetic barrier to resistance to be a viable SCT option, particularly when partnered with TNV [12,13].

The BREATHER Plus trial aims to demonstrate whether having weekends off DTG based ART with a ‘backbone’ that includes TNV and 3TC/FTC works as well as taking daily ART in ALHIV. If triple-therapy SCT is as effective as daily oral therapy, it will provide a new treatment strategy for ALHIV, which may improve their longer-term outcomes.

## 2 Methods

### 2.1 Objective and hypotheses

The BREATHER Plus trial is evaluating the virologic efficacy, safety, acceptability and quality of life on DTG/TNV based SCT with weekends off compared with CT on DTG/TNV based ART. The trial’s hypotheses are that SCT will provide non-inferior virologic suppression over 96 weeks compared to CT and will be superior with respect to secondary outcomes including toxicity, acceptability, and quality of life (QoL).

### 2.2 Study design, participants and randomisation

The BREATHER Plus trial is a randomised open-label 2-arm, 96-week trial evaluating SCT (five days on, two days off) DTG/TNV-based triple ART compared to daily ART in virologically suppressed ALHIV. The full protocol can be accessed at https://www.mrcctu.ucl.ac.uk/studies/all-studies/b/breather-plus/.

The trial enrolled ALHIV from Uganda, Kenya, South Africa and Zimbabwe, aged between 12 to <20 years, who were on ART and virologically suppressed for the last 12 months, with no history of treatment failure, and on a DTG/TNV based triple therapy regimen for at least one month at screening. Previous ART regimen substitutions due to toxicity, simplification, changes in guidelines or drug availability were not considered as treatment failure, and hence were not exclusions. Pregnancy and breast-feeding were reasons for exclusion and girls who were sexually active had to be on highly effective contraception. Contraception was accessed locally through standard care, which was confirmed to be available at all participating sites (Table 2).

Participants were randomised 1:1 to SCT (intervention arm), or CT (control arm). Randomisation was stratified by clinical centre and mode of infection (horizontal or vertical). The computer-generated randomisation list was prepared using permuted blocks and incorporated securely into the trial database, concealed from local staff. Allocation for each patient was made automatically through the web-enabled database after eligibility had been confirmed by site staff.

### 2.3 Treatment of study participants

Adolescents in both trial arms receive DTG/TNV based triple ART in line with WHO guidelines [22]. Those in the CT arm take their ART daily while those in the SCT arm take their ART for 5 consecutive days a week (Monday to Friday or Sunday to Thursday) and miss ART for the same 2 consecutive days every week. All participants were on TLD at enrolment (tenofovir/lamivudine/dolutegravir, a fixed dose combination of DTG (50 mg), TDF (300 mg) and 3TC (300 mg)). The protocol allowed for TAF (25 mg) in place of TDF if it became available during the trial, and/or FTC (200 mg) instead of 3TC [23]. This was to allow additional flexibility; as of April 2025, TAF had not been used in the trial.

### 2.4 Primary and secondary outcomes

The primary outcome is confirmed viral rebound (defined as the first of two consecutive HIV-1 RNA ≥50 c/mL) by week 96. This outcome was chosen as an objective and clinically relevant measure of the loss of virologic suppression. Secondary outcomes are listed in Table 3. The primary analysis is planned employing an intention-to-treat (ITT) approach; refer to Primary Analysis section below.

### 2.5 Sample size

Non-inferiority of SCT will be assessed by the difference between the SCT and the CT arms in the estimated proportion of participants with confirmed viral rebound (defined as the first of two consecutive HIV-1 RNA ≥ 50 copies/mL) by week 96.

The trial was designed with a fixed non-inferiority margin of 10 %. At design it was estimated a total of 460 participants (230 per group) would provide 90 % power, 2-sided alpha of 5 %, to demonstrate non-inferiority of SCT vs. CT, assuming 11 % of participants met the primary endpoint by the 96-week assessment in both groups [24] and allowing for 10 % loss to follow-up. Assumptions for the sample size calculations were made based on the BREATHER trial results on efavirenz-based regimens [6].

To allow for a lower than anticipated control event rate, we will use the Smooth Away from Expected (SAFE) frontier [25]. Provided that the observed viral rebound rate in the control arm is not lower than 9 %, a 95 % two-sided confidence interval will be computed for the difference in viral rebound between SCT and CT groups and a 10 % non-inferiority margin will be used. If the observed rate of viral rebound in the control arm is less than 9 %, a 99 % two-sided confidence interval will be computed for the difference in viral rebound between SCT and CT groups; the non-inferiority margin will depend on the control event rate as shown in Appendix F. If the upper bound of the respective confidence interval is not higher than the selected non-inferiority margin, then the null hypothesis will be rejected and SCT will be declared non-inferior to control.

### 2.6 Recruitment and follow-up

**Fig. 1:**
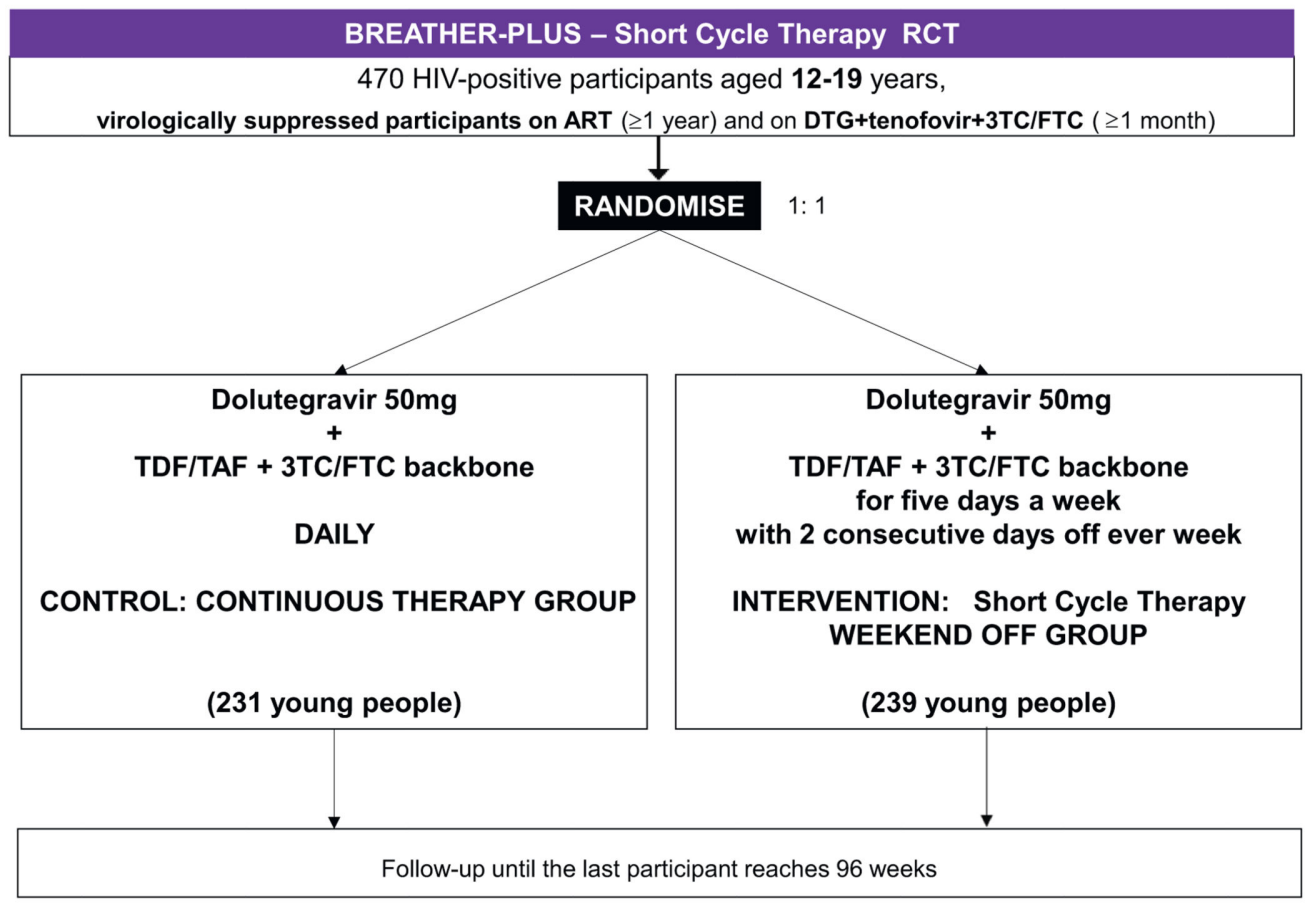
Trial Schema. Abbreviations: DTG = dolutegravir; TDF = tenofovir disoproxil fumarate; TAF = tenofovir alafenamide fumarate; 3TC = lamivudine; FTC = emtricitabine; RCT = randomised controlled trial.

Recruitment was conducted in outpatient clinics, where significant numbers of ALHIV are treated. The trial enrolled 470 participants (231 SCT and 239 CT, see Fig. 1) from five sites in four countries between June 2022 and May 2023 (see Appendix E). Participants will be followed up until the last enrolled participant reaches 96 weeks (12 March 2025). Each participant will attend an end-of-trial visit at ≥96 weeks from enrolment and within ±6 weeks of the last participant reaching 96 weeks, meaning individual participants will be followed up for between 96 and 144 weeks, depending on how early in the trial they were recruited.

### 2.7 Study procedures

Participants were seen at screening, enrolment, week 4 (SCT participants only), and weeks 8, 16, 24, 32, 40 and 48, and will continue to be seen every 12 weeks until the end of follow-up (Table 4). Visits were more frequent in the first year of the trial to allow for regular, 8-weekly pregnancy testing in girls, due to concerns based on observational data linking DTG use during pregnancy to an increased risk of neural tube defects [26]; full study visits were carried out for all participants at each 8-weekly visit. In the light of updated safety data [27–29] and national guidelines, visits, and the corresponding frequency of pregnancy testing, were relaxed to 12-weekly after week 48. The trial started enrolling during the Coronavirus disease 2019 (COVID-19) pandemic, with a mitigation strategy in case people could not attend scheduled appointments, however, this was not needed. In 33 participants, week 1, 2, 3 and 4 visits were also carried out as part of a pilot phase, see Statistical Analysis Plan.

A real time HIV-1 VL was measured at the sites at the screening visit to confirm eligibility. Frequency of real time VL testing during follow-up employed a pragmatic, standard-of-care based approach to maximise generalisability of findings across SSA; In Uganda [30] and Kenya [31], per local guidelines, testing was required at weeks 24, 48, 72 and 96 and 24-weekly thereafter (until age 20 in Uganda, when testing frequency reduces to 48-weekly).Testing was only required at weeks 48 and 96 and 48-weekly thereafter in Zimbabwe [32] and South Africa [33] (with a supplemental 24-week test in Zimbabwe). Participants who have a real time VL ≥ 50 c/mL are brought back to clinic for confirmatory testing, within 4 weeks of the target visit date prior to week 48 and within 6 weeks of the target visit date from week 48 onwards. Targeted real-time testing is also performed for suspected treatment failure.

Provision of more frequent VL testing would likely alter patient management, meaning trial results would not apply to most of sub-Saharan Africa where routine monitoring is 6–12 monthly, depending on country-level guidance. Stored plasma samples are used for retrospective VL testing at all other timepoints. A combination of real-time and retrospective VL results are used to inform the trial endpoints and for review by the Independent Data Monitoring Committee (IDMC). The results on the stored samples are not returned to treating clinicians to inform clinical care but will be shared with participants and their clinicians at the end of the trial.

At the end of the trial, batched genotypic resistance testing will be performed retrospectively on stored samples from all participants who have met the primary outcome. Drug resistance mutations [34,35] will be classified using the latest IAS-USA definitions and drug susceptibility predicted using the latest version of the Stanford database algorithm. Individual results of resistance tests will be given to the treating physician when they become available.

### 2.8 Questionnaires

The trial utilises participant questionnaires (see Appendix B) to evaluate participant adherence to treatment strategy, acceptability, mood and sleep, suicidal ideation and behaviors and health-related quality of life (QoL).

### 2.9 Criteria for discontinuing or modifying allocated interventions

Participants on SCT who have confirmed real-time VL ≥50 c/mL, who become pregnant, have incident TB or stop study investigational medicinal product (IMP, i.e. DTG/TNV plus 3TC/FTC) must return to CT. Following end of breastfeeding or pregnancy (if not breastfeeding) or TB treatment, they may resume SCT. Participants with confirmed real-time VL ≥50 copies/mL on SCT do not return to SCT when their VL suppresses.

### 2.10 Modifying ART

A participant may discontinue the trial IMP if their current treatment fails, or in case of drug toxicity, intercurrent illness, or any change in the participant’s condition that justifies IMP discontinuation in the clinician’s opinion. Treatment changes will be made according to local guidelines. In case of incident tuberculosis co-infection after enrolment in the trial, the dose of DTG will be doubled (100 mg/day taken as DTG 50 mg twice daily) for the duration of rifampicin treatment. With the accrual of more safety data for DTG use during pregnancy, DTG-based regimens are preferred during pregnancy and breast-feeding [22,30,36]. However, some participants may choose to switch off DTG during pregnancy and will be supported by their clinician.

### 2.11 Safety management

Reportable Adverse Events (AEs) in the trial include Serious Adverse Events (SAE), clinical grade 3/4 AEs, laboratory grade 3/4 AEs that are clinically significant, WHO stage 3/4 events, AEs of any grade that lead to the modification of ART, and any suicidal ideation that includes method, intent or plan or any suicidal behaviour. At clinic visits, AEs are screened for using a symptom checklist, completing a clinical assessment, review of laboratory results and, completing a suicidality assessment as per the trial schedule. AEs are graded using the Division of AIDS Table for Grading the Severity of Adult and Paediatric Adverse Events [37].

Pregnancies and suspected cases of drug-induced liver injury are reported as Notable Events (NE). Participants with AEs are followed up until clinical resolution or until the event has stabilised. Pregnancies are followed up to completion, with infants followed up to 4–6 weeks post birth.

Both SAEs and notable events are reported to Sponsor within expedited timelines. SAEs are reported to regulatory agencies as per national requirements. Pregnancy outcomes are reported to the Antiretroviral Pregnancy Register [38].

### 2.12 Strategies to improve adherence

Adherence is checked by pill count done by trial nurse or pharmacist; and short, trial-specific, participant self-administered adherence questionnaires. Participants may receive adherence counselling as per site standard of care, independent of randomised allocation.

### 2.13 BREATHER plus sub-studies

Integrase inhibitors including dolutegravir are associated with neuropsychiatric side-effects. The Neuropsychiatric (NP) toxicity sub-study aims to assess the burden of NP problems among participants, including depression, suicidality, anxiety and sleep disturbance, and to compare symptoms between trial arms, where reduced exposure in the SCT arm might be associated with less side-effects. A short tool, The Mood survey Questionnaire (MSQ) (Appendix B, Fig. S4), and the Columbia-Suicide Severity Rating Scale (C-SSRS) are administered longitudinally to identify mental health issues. The MSQ was developed as a quick and easy to administer tool and will be assessed for sensitivity and specificity in identifying issues in a sample of participants, by comparison with the Patient Health Questionnaire-9 (PHQ-9) for depression, the Generalized Anxiety Disorder-7 (GAD-7) for anxiety and a Sleep survey questionnaire (Appendix B, Fig. S5).

In the Medication Event Monitoring Systems (MEMS Caps) sub-study, participants were selected across Ugandan and Kenyan sites to measure compliance with the protocol during weeks 8–32 (targets, 50 SCT; 50 CT) and weeks 48–72 (targets, 50 SCT; 50 CT). The sub study utilises special bottle caps with a small electronic device embedded inside that fit on standard size ART pill bottles and record the time and date of each bottle opening as a presumptive dose. MEMS Caps data will provide objective data on whether SCT participants are opening their pill bottles 5 days a week and CT participants, 7 days a week.

The Social science sub-study will quantitatively and qualitatively assess secondary trial outcomes of adherence, acceptability of treatment strategy and well-being among participants on SCT versus daily oral ART. All participants complete adherence questionnaires as part of the wider trial procedures. In addition, questionnaires on acceptability, wellbeing and Quality-of-life are completed as per Table 4. Some participants from Uganda and South Africa will be invited to participate in Focus Group Discussions (FGDs) and there will be longitudinal in-depth interviews (IDIs) in South Africa. FGDs and IDIs aim to assess acceptability and experience of treatment strategies, impact of treatment strategies on adherence and well-being and relationships and experience of side-effects.

The costs and cost-effectiveness of the trial’s treatment strategies will be evaluated in a health economics sub-study using a generic health measure (quality-adjusted life-years (QALYs) measured by the EQ-5D) to allow comparison with other interventions. Resource use and total costs from a health system perspective will be estimated using trial data and other sources (e.g. unit costs/prices) to be representative of general roll-out in African countries. Cost-effectiveness will be assessed using incremental cost-effectiveness ratios and compared to appropriate country specific cost-effectiveness thresholds.

Annual blood and urine storage samples have been taken with consent and predicated upon funding, will be used to explore changes in renal function/metabolic parameters/bone health/inflammatory markers/ART levels in a metabolic, renal and bone parameter sub-study.

## 3 Data management

The trial database is in OpenClinica and access is controlled. To protect participants’ confidentiality, participants were assigned a trial identification number and a random three-letter code. The database incorporates checks for eligibility, missing data and ranges, and additional consistency checks are performed by trial statisticians.

## 4 Statistical analysis plan

The final BREATHER plus statistical analysis plan (V2.0) is included in Appendix G and summarised here.

### 4.1 Pilot phase and IDMC oversight

BREATHER Plus started with a pilot phase, in which the first 16 participants randomised to SCT and 17 randomised to CT at sites had real-time VL measurements at weeks 1, 2, and 3 (and a confirmatory VL at week 4 following a single VL ≥50 c/mL at week 3) with measures in the SCT group performed after weekends off. The Independent Data Monitoring Committee (IDMC) reviewed VL data after all pilot participants reached week 4 and determined the trial could continue. Recruitment continued during the pilot phase.

The IDMC has since met three times (February 2023, October 2023, June 2024) to review safety and efficacy data by trial arm. Batched runs of VLs were conducted prior to each IDMC meeting.

### 4.2 Primary analysis

For the primary analysis, the two treatment groups will be compared in the intention-to-treat (ITT) population. The comparison will be of the cumulative probability of virologic rebound by week 96. To allow for censoring, the survival curve for each combination of strata and randomised group will be calculated using a Cox model adjusting for stratification factors (as appropriate) and randomised group. The average cumulative failure function (1-survival curve) for each randomised group will be estimated by standardisation [39] as a weighted average of the corresponding stratum-specific cumulative failure functions with weights equal to the prevalence of that stratum in the trial population. The difference in the probability of virologic rebound between the SCT group and the CT group will be estimated by the difference between the averaged cumulative failure functions at week 96. A 2-sided bias-corrected 95 % or 99 % CI (Appendix F) for the difference in the probability of confirmed viral rebound by week 96 (SCT–CT) will be calculated using appropriate (bias-corrected) percentiles of the bootstrap estimates. The bootstrapping will sample 10,000 times and be stratified by stratification factors. SCT will be considered non-inferior to CT if the upper limit of the respective CI of the difference SCT-CT is less than the selected non-inferiority margin (Appendix F). For analysis of the primary endpoint and other virological outcomes, except for the FDA snapshot analysis, multiple imputation of missing HIV-1 RNA measurements at scheduled visits will be applied if either of the following criteria are met: 5 % of all HIV-1 RNA measurements at scheduled visits are missing or 10 % of confirmatory HIV-1 RNA measurements are missing.

The modified FDA snapshot algorithm will be used to compare the proportion with virological rebound at 48 and 96 weeks. Other secondary outcome measures will be compared for superiority between the SCT and CT groups using appropriate statistical methods in the intention-to-treat population.

A per protocol analysis will be undertaken excluding any participants who did not meet all the eligibility criteria and any participants who reported taking <75 % intended weekend breaks (SCT arm) or spending <90 % of time on ART (CT arm) to earliest of 96 weeks or censoring date. Follow-up will be censored if a participant had a break in any component of ART regimen for more than 7 days, changed ART regimen or switched to CT for reasons other than confirmed viral rebound (SCT arm).

## 5 Trial oversight

1. The IDMC are independent clinical trial experts who review interim analyses of accumulating data by trial arm. The IDMC will advise the Trial Steering Committee (TSC) if the trial should be stopped for safety or other reasons.
2. The Trial Steering Committee (TSC) are members from The BREATHER Plus Consortium plus independent members, including independent Chair and Patient and Public Involvement (PPI) contributors. The TSC provides overall supervision and advice for the trial. The ultimate decision for continuation of the trial lies with the TSC.
3. The BREATHER Plus Consortium, responsible for the day-to-day running and management of the trial, comprises the Trial Chief Investigator (Chair), site Principal Investigators, co-investigators and trial Managers, sub-study leads and members of the Medical Research Council Clinical Trials Unit (CTU) at UCL and PPI contributors.
4. Trial Management Teams (TMTs) at MRC CTU at UCL and sites conduct the trial and ensure that regulatory processes are followed.

## 6 Patient and public involvement (PPI)

The trial teams engage with existing PPI groups at sites, including Youth Trial Boards (YTBs) in Uganda, South Africa and Zimbabwe, in place from previous trials. YTBs consist of individuals aged 15–19 years, who are supported by local coordinators to ensure that the voices of ALHIV are heard, and they contribute meaningfully to the development, delivery and dissemination of paediatric clinical trials within their communities. They contributed to the development of user-friendly Participant Information sheets (Appendix D), infographics (Appendix C) and questionnaires (Appendix B, Fig. S4) used in the BREATHER Plus trial, and to the development of the communications strategy. Two young people who are former YTB members are now non-voting independent members on the TSC.

## 7 Current status of the trial

Enrolment of 470 participants completed between June 2022 and May 2023. Follow-up is ongoing, with close-out visits planned between January and April 2025. The MEMSCaps study fully recruited in Uganda and Kenya.

## 7.1 Baseline characteristics

Median age at enrolment was 16 years (IQR 15–18); 56 % participants were female (Table 5). Most participants were vertically infected (97 %). Median age at ART initiation was 4 years (IQR 2–8) and participants had been on TLD for median 2 years (IQR 2–3) at enrolment.

## 8 Discussion

BREATHER Plus is the first randomised trial evaluating SCT (five days on, two days off) using entirely DTG/TNV based triple ART. It evaluates an innovative strategy aimed at enabling continued ART adherence and better QoL among adolescents [40]. Moreover, reduced exposure to ART will potentially reduce toxicities, for example neuro-psychiatric symptoms, and cost.

ALHIV are a special population who may benefit from SCT treatment. On average, they remain on ART for longer than those who acquire HIV in adulthood, placing them at higher risk of accumulating long-term toxicities [24] and experiencing treatment fatigue. Additionally, they are undergoing rapid growth, pubertal and neurodevelopmental maturation, and may benefit from reduced exposure to drugs through SCT in different ways. Taking weekends off treatment could help young people living with HIV to socialise without worrying about disclosure and offer some freedom from the burden of lifelong daily treatment. Conversely, as adolescents struggle more than adults with treatment adherence [41] there is a risk they may miss more than the 2 regular days off ART thus risking viral rebound, the emergence of drug resistance, limiting future treatment options. BREATHER Plus 96-week comparative data will provide the necessary evidence as to whether SCT can be used safely in virologically suppressed ALHIV.

To ensure the relevance of findings to diverse settings, the trial is taking place in four sub–Saharan African countries where most ALHIV live [42]. The trial uses a pragmatic approach to real-time VL testing aligned with national guidelines; additional retrospective VL testing of stored samples is performed to determine the trial’s virological outcomes and for review by the IDMC, who oversee participant safety. For the primary endpoint of confirmed viral rebound, we use the time of the first HIV-1 RNA of two raised VLs to minimise the impact of different schedules for routine VL monitoring across sites and have considered when imputation might be needed in case of missing data. The trial also addresses the commonly cited reasons for not using SCT in low- or middle-income countries, including the need for co-treatment of TB and management of pregnancies.

BREATHER Plus trial is a non-inferiority trial which aims to demonstrate that SCT efficacy is not unacceptably lower than CT. The choice of the non-inferiority margin is critical to determine the acceptable loss of efficacy and is likely to depend on the control event risk. [25,43]; the non-inferiority margin and significance level will be modified based on the observed confirmed viral rebound risk in the control arm, while preserving power close to 85 % and controlling for type 1 error. This methodology was developed alongside the Statistical Analysis Plans for BREATHER Plus (Appendix G) and the D3/Penta 21 trial [44]. It maintains interpretability of results if the control event risk differs from assumptions, while accommodating the constraints of a fixed sample size in an already funded trial.

## 9 Limitations

BREATHER Plus includes a highly adherent, virologically suppressed, DTG-tolerant participant population, potentially limiting generalisability to all ALHIV. However, while rates of virological suppression are lower in ALHIV than in adults living with HIV, TLD offers a highly effective and tolerable regimen and is now in wide use, and the majority of ALHIV on TLD are virologically suppressed [45]. Thus, results will apply widely among ALHIV, and if SCT is efficacious and safe, can likely be extrapolated to adults suppressed on TLD.

Although we planned to enrol ≥30 % participants with horizontally acquired HIV, this was not achieved despite repeated attempts by all sites to ‘find’ these participants. While there is no reason to think that mode of acquisition impacts biological response to ART, it is possible that behaviour, including adherence, differs between ALHIV who have vertically or horizontally acquired HIV. However, in a small single arm study of SCT, virological outcomes were similar in young people with perinatally acquired HIV or HIV acquired in early childhood versus those who acquired HIV horizontally through risk behaviors, but by 48 weeks rates of virological rebound were high (38 %) in those infected perinatally. [15]

Lastly, the SCT intervention is being tested in an open-label trial because ability to adhere to the strategy is critical for future implementation, and assessment of the effects of SCT on participant-reported outcomes including quality-of-life and acceptability is key. However, there are risks that participants in the CT arm, having been informed of the trial design, may choose to take weekends-off, and participants in the SCT arm may choose to continue daily ART. The team have done their best to mitigate this through patient information, supported by the YTB, and through ongoing adherence counselling for both arms. In addition, adherence to allocated strategy will be measured by the adherence questionnaires and, in subsets, the qualitative work and the MEMS Caps sub study.

## 10 Conclusions

BREATHER Plus will evaluate DTG/TNV-based SCT for sustaining virological suppression over ~2 years in ALHIV in four African countries. With current ART objectives aimed at reducing side effects and improving QoL, the trial will contribute information for increasing treatment options for this population. Sub-studies will provide valuable insights into neuropsychiatric toxicities, adherence, acceptability and cost-effectiveness of SCT vs. CT.

## Supplementary Material

Supplementary data to this article can be found online at https://doi.org/10.1016/j.cct.2025.107963.

Appendix

## Figures and Tables

**Table 1:** Previous studies evaluating efficacy of SCT.

	Study	Year Published	End points	Results
Single Arm Trials				
1	FOTO study [14]: Single arm pilot study of SCT (5 days on. 2 days off) in 30 adults in USA on either NNRTI (efavirenz (*n* = 10) or nevirapine (10)) or PI (10) based regimens VL monitoring at weeks 4, 12, 24, and at least 12-weekly thereafter	2007	VL <50 copies/mL at weeks 48 weeks (primary endpoint) and 24 weeks in as-treated population	Week 24, 90 % (26/29) Week 48, 88 % (23/26); no new failures between weeks 24–48
2	Adolescent Trials Network (ATN) study [15]: Single arm study of SCT (4 days on, 3 days off) in 32 adolescents and young adults (12 to <24 years) on a PI-based regimen. 22/32 had received ≥5 ART regimens at enrolment. VLs 4-weekly for 24 weeks and 8-weekly thereafter	2009	Confirmed VL >400 c/mL by 48 weeks	38 % (12/32) had confirmed VL rebound by 48 weeks
3	The ICCARRE project [16]: Single arm study of SCT (reduction to 5, 4, 3, 2 days on ART) in 94 adults on France on either triple or quadruple ART (63 % PI or NNRTI +2 NRTIs; 25 % NNRTI +3 NRTIs, 11 % raltegravir-based triple or quadruple ART) VL monitoring depended on number of days on treatment but at least 12-weekly	2015	Confirmed VL rebound of ≥50c/mL	Viral suppression was maintained on 4-day a week regimen (mean 87 weeks in 94 patients). Viral rebounds were observed on regimens where ART was given <4 days per week
4	The ANRS 162-4D trial [17]: Single arm study of SCT (4 days on, 3 days off) in 100 adults on triple ART (21 PI, 71 NNRTI-based ART) VLs 4-weekly for 16 weeks and 8-weekly thereafter	2018	Remained in the study with VL < 50 c/mL up to week 48 Virological failure (VF) defined as confirmed VL ≥ 50c/mL	96 % of participants had no failure and remained on SCT; 3/100 participants experienced virological failure and 1/100 stopped SCT for patient choice
Randomised Controlled Trials (RCT)				
5	Randomised FOTO trial [18]: RCT comparing SCT (5 days on, 2 days off) vs CT on efavirenz/tenofovir/emtricitabine in 60 adults VLs at weeks 4, 12, 24	2009	VL <50 copies/mL at week 24	SCT 100 % (25/25), CT 86 % (24/28)
6	Ugandan SCT trial [9]: 3-arm RCT comparing SCT-1 (7 days on, 7 days off) and SCT-2 (5 days on, 2 days off) vs CT in adults in Uganda (98 % on NNRTI based regimens). Recruitment target – 57 per group. VLs at weeks 2, 4, and 6-weekly thereafter	2010	Primary endpoint was treatment failure by 72 weeks (VL ≥ 10,000 c/mL or 2 consecutive VL ≥ 1000 c/mL or VL ≥ 400 c/mL at 72 weeks, 30 % drop from baseline in CD4 on 2 consecutive measures, death due to treatment, or opportunistic infection). VL measured 6-weekly.	SCT-2 11.5 % (6/52, 5 VL failures), CT 22 % (11/51, 9 VL failures); inferiority of SCT-2 was rejected SCT-1 was stopped early due to a high failure rate
7	BREATHER Trial [6,7]: RCT comparing SCT (5 days on, 2 days off) vs CT on efavirenz-–based ART in 199 young people aged 8 to 24 years from 11 countries (35 % from Uganda) VLs 12-weekly	2018	Confirmed VL rebound of ≥50c/mL by 48 weeks (primary endpoint) and by 144 weeks (extended follow-up)	Week 48: SCT 6 % (6/99), CT 7 % (7/100), difference (SCT-CT) -1.2 % (90 %CI -7.3, 4.9) Week 144: SCT 15 % (15/99), CT 13 % (13/100), difference (SCT-CT) 1.9 % (90 %CI -6.6, 10.4)
8	A-TRI-WEEK [19]: Pilot RCT comparing SCT (3 days on [Monday, Wednesday, Friday], 4 days off) vs CT on efavirenz-based ART in 61 adults in Spain with an open-label extension period VLs 1, 2, 4, 6, 8, 12 and 24 weeks on SCT; 12 and 24 weeks on CT	2018	Treatment failure to week 24 (any of confirmed VL ≥ 37 c/mL, discontinuation of ART schedule for any reason, study withdrawal/lost to follow-up for any reason, pregnancy)	Week 24: SCT 0 % (0/30), CT 0 % (0/31), difference (SCT-CT) 0 % (95 %CI -14.1, 14.1)
9	The AlTernAte Days (ATAD) [20]: RCT comparing alternate days vs CT on efavirenz-based ART in 196 adults in Italy VLs 4-weekly to 12 weeks, then 12-weekly to 48 weeks	2019	Confirmed virological response (VL < 40 c/mL per FDA-defined time to loss of virological response [TLOVR] algorithm) by week 48.	Week 48: 93.9 % (93/99) ATAD, 96.9 % (95/98) CT, difference (ATAD-CT) -3 % (95 %CI -8.86, 2.86)
10	The ANRS 170 QUATUOR study [8]: RCT comparing SCT (4 days on, 3 days off) vs CT on triple ART in 636 adults (modified ITT population) in France Third agents included INSTI (48 %), NNRTI (47 %), PI (6 %) VLs at weeks 4, 12 and 12-weekly thereafter	2019	VL <50c/mL at 48 weeks (FDA snapshot, primary endpoint). Virological failure (VF, confirmed VL rebound of ≥50c/mL) at week 48 (secondary), and, in those on SCT, at week 96	Week 48, VL < 50: SCT 96 % (304/318), CT 97 % (308/318), difference (SCT-CT) -1.3 % (95 % CI -4.2, 1.7) Week 48, VF: SCT 1.9 % (6/318), CT 1.3 % (4/318), difference (SCT-CT) 0.6 % (-1.3, 2.6). VF on INSTI were SCT 2 % (3/152), CT 0.7 % (1/152) Week 96, VF: SCT 4 % (13/318); including 2 % (3/152) on INSTIs (0/73 on DTG)
11	Proof of concept FOTO RCT with bictegravir [10]: RCT comparing SCT (5 days on, 2 days off) vs CT on bictegravir/tenofovir alafenamide/emtricitabine in 60 adults in Taiwan VLs at weeks 4, 28, 52	2024	BIC C_trough_ > 162 ng/mL (primary) at weeks 4, 28, 52 VL < 50c/mL at weeks 4, 28, 52	90 %, 93 %, 100 % in SCT group maintained effective trough concentrations at weeks 4, 28, 52 VL < 50: SCT, 100 %, 93 %, 100 %, CT 97 %, 93 %, 97 %
12	BEFTAF-RED [21]: Pilot RCT comparing 3 [Monday, Wednesday, Friday], 2[Tuesday, Friday] or 1[Wednesday] dose per week vs CT on bictegravir-based ART in 40 adults in Spain VLs 12-weekly	2025	VL <50 c/mL (FDA snapshot) at 12 and 48 weeks (on-treatment and ITT populations)	Week 12 VL < 50 c/mL on-treatment: CT 100 % (9/9), 3 days 90 % (9/10), 2 days 100 % (10/10), 1 day 80 % (8/10) Week 12 VL < 50 c/mL ITT: CT 100 % (9/9), 3 days 90 % (9/10), 2 days 100 % (10/10), 1 day 100 % (10/10) Week 48 VL < 50 c/mL on-treatment: CT 89 % (8/9), 3 days 90 % (9/10), 2 days 90 % (9/10), 1 day 70 % (7/10) Week 48 VL < 50 c/mL ITT: CT 89 % (8/9), 3 days 100 % (10/10), 2 days 100 % (10/10), 1 day 90 % (9/10)

Abbreviations: SCT = short cycle therapy; NNRTI = non-nucleoside reverse transcriptase inhibitor; PI = protease inhibitor; NRTI = nucleos(*t*)ide reverse transcriptase inhibitor; INSTI = integrase inhibitor; BIC = bictegravir; ART = Antiretroviral Therapy; VL = viral load; RCT = randomised controlled trial; ITT = intention-to-treat; VF = virological failure.

**Table 2:** Participant inclusion and exclusion criteria.

Inclusion Criteria
HIV-1 positive Aged 12 to 19 years Aware of HIV status On ART for ≥1 year No previous regimen change for treatment failure On ART consisting of DTG, tenofovir and lamivudine/emtricitabine for ≥1 month prior to screening Virologically suppressed with all HIV-1 RNA viral loads <50copies/mL^a^ in the last 12 months up to and including screening. Additionally, there must be one result <50copies/mL at least 12 months prior to screening and the viral load at trial screening must be <50 copies/mL Girls who are sexually active must be willing to adhere to highly effective methods of contraception^b^ Written informed consent provided by participant (if aged 18 to 19 years) and/or carer/legal guardian (if participant aged 12 to 17 years) as appropriate Written informed assent in participants aged 12 to 17 years
Exclusion Criteria
Females who are pregnant or breastfeeding Females who plan to become pregnant during the trial follow-up or are unwilling to use a highly effective method of contraception for the duration of the trial if sexually active. Moderate or High-risk score on the Columbia-Suicide Severity Rating Scale On treatment for any active tuberculosis (TB) Contraindication to continued receipt of dolutegravir or any formulation of tenofovir, lamivudine/emtricitabine Underlying medical condition that in the opinion of the Investigator precludes participation Previous randomisation in the LATA trial^c^

Abbreviations: HIV-1 = human immunodeficiency Virus-1; DTG = dolutegravir; ART = antiretroviral therapy; TB = tuberculosis; LATA = long-acting treatment in adolescents: A randomised, open-label, two-arm, 96 week trial in virologically suppressed HIV-1-positive adolescents aged 12–19 years of age in sub-Saharan Africa.

a If a historic viral load is from a diluted sample (maximum dilution 1:5), and below lower limit of quantification (LLQ), a calculated VL < 100 copies/mL is allowed; if the viral load in the diluted sample is equal to the LLQ, the calculated VL should be below 50 copies/mL. If there are any viral loads measured on dried blood spots since the most recent viral load on plasma more than 12 months ago these must be below the LLQ for the assay used. The screening sample viral load must always be <50 c/mL and cannot be done using a dry blood spot.

b Highly effective contraception are injectable, implantable, oral and intrauterine contraceptives which have an expected failure rate < 1 % per year.

c Clinical Trials registration, NCT05154747.

**Table 3:** Primary and secondary outcome measures.

Primary outcome measure
▪ The proportion of participants with confirmed virological rebound*, defined as 2 consecutive plasma HIV-RNA ≥50 copies/mL at any time up to the 96-week assessment.
Secondary outcome measures
Efficacy
▪ Proportion of participants with HIV-RNA ≥50 copies/mL at 48 and 96 weeks using the modified Food and Drug Administration (FDA) snapshot algorithm ▪ The proportion of participants with HIV-RNA ≥1000 copies/mL (confirmed) by week 96 ▪ The number and type of HIV mutations at confirmed virological rebound** ▪ HIV-RNA <50 copies/mL and no switch to second-line ART for treatment failure at 24, 48, 72 and 96 weeks
Safety
▪ Change in toxicity profile including change in metabolic parameters (lipids, glycosylated haemoglobin (HbA1c), phosphate), renal function (estimated Glomerular Filtration Rate (eGFR) from baseline to 96 weeks; change in anthropometric measures from baseline to 48 and 96 weeks ▪ Time to any new or recurrent WHO stage 3 or WHO stage 4 event or death ▪ Incidence of serious, grade 3, 4 and 5, and treatment-modifying (of any grade) adverse events ▪ The proportion of participants with any change from baseline ART regimen
Patient Reported Outcomes
▪ Adherence, acceptability, wellbeing and neuropsychiatric toxicities and neuropsychiatric problems (e.g. depression, anxiety and sleep disturbance) ▪ Healthcare resource utilisation (as a sub-study outcome) ▪ Health-related quality-of-life (as a sub-study outcome

Abbreviations: HIV RNA = human immunodeficiency virus ribonucleic acid; WHO = World Health Organization; ART = antiretroviral therapy; FDA = Food and Drug Administration; HbA1c = glycosylated haemoglobin; eGFR = estimated glomerular filtration rate. virological rebound is defined as two consecutive HIV-RNA ≥50 copies/mL.

* virological rebound is defined as two consecutive HIV-RNA ≥50 copies /mL.

** As obtaining resistance data is challenging when viral loads are very low, resistance testing may need to be restricted to samples with a higher VL where the chances of being able to sequence are greater; this will also be dependent on available technologies for testing.

**Table 4:** Trial Assessments.

Study week number	Screening	Randomisation	W0	W4 (SCT-only)	W8	W16	W24	W32	W40	W48	W60	W72	W84	W96	Followup beyond W96	Close-out visit
Signed Informed consent	X	Confirm														
Clinical assessment [1]	X	X	X	X	X	X	X	X	X	X	X	X	X	X	Every 12 weeks	X
Vital Signs			X		X		X			X				X	Every 48 weeks	X
Dispensing antiretroviral drugs (Trial IMP)			X	X	X	X	X	X	X	X	X	X	X	X	Every 12 weeks	X
Laboratory Assessments																
Pregnancy test (urine) - only female participants [2]	X	X	X	X	X	X	X	X	X	X	X	X	X	X	Every 12 weeks	X
HBsAg Screening	Done once for all participants, except for female participants who become pregnant, where it is repeated.															
HIV-1 RNA VL [3]	X						(X)			X		(X)		X	Every 48 weeks	X
Haematology [4			X							X				X		
Biochemistry [5]			X											X		
Lipids (same draw as biochemistry)			X											X		
HbA1c			X							X				X	As per local practice	
T-cell lymphocyte subsets (same draw as haematology) [6]			X							X				X	Every 48 weeks	
Other Assessments																
Adherence assessment [7]			X	X	X	X	X	X	X	X	X	X	X	X	Every 12 weeks	X
Acceptability questionnaire (HATQoL questionnaire)			X				X			X				X	Every 48 weeks	X
Mood survey			X		X		X			X		X		X	Every 48 weeks	X
C-SSRS questionnaire	X	X			X		X			X		X		X	Every 48 weeks	X
EQ5D			X				X			X				X		X
Sample Storage																
Mandatory plasma storage sample [8]			X	X	X	X	X	X	X	X	X	X	X	X	Every 24 weeks	X
Optional plasma storage sample [9]			X							X				X		
Urine storage sample [9]			X											X		
MEMS Caps [10]					X	X	X	X		X	X	X				

Every effort is made to minimise loss to follow up. Participants who miss clinic visits are traced using home visits and mobile phone calls. If a participant or their carer chooses to discontinue participation in the trial, prior to transferring to routine clinic follow-up, the participant will be asked to have assessments performed as appropriate for a close-out trial visit, although they would be at liberty to refuse any or all individual components of the assessment. It shall be discussed with the participant and their carer whether they are willing to be contacted in the future, in order to collect routine data for the trial, or, if not, whether data may be collected from their medical notes. () Optional if done in routine care. [1] Clinical assessment includes medical and ART history, clinical examination, weight, height, mid upper arm circumference, waist circumference, paediatric WHO staging for HIV and adverse events (starting from week 0). [2] For girls who have reached menarche.[3] Real-time/local VL to be done at screening, weeks 48 and 96 and then every 48 weeks (with confirmatory VLs for HIV-1 RNA ≥50 c/mL); more frequent VLs may be done if site routine VLs are more frequent. An additional VL is required if treatment failure is suspected. Retrospective VL testing is performed using routine stored plasma [15][15] at the scheduled trial visits when a real-time VL is not done. [4] Haematology: haemoglobin, red blood cells, white blood cells, lymphocytes, neutrophils, platelets. [5] Biochemistry: urea, creatinine, albumin, alanine transaminase, aspartate transaminase, bilirubin. [6] CD3+, CD4+, CD8+ T-lymphocyte percentage and absolute, total lymphocyte count. [7] Pill count (except week 0) and adherence questionnaire [8] At all scheduled trial visits plasma samples are stored for future batch testing for retrospective VL, and low-level viremia and resistance testing in a subset of samples. Additionally, a plasma sample is stored at unscheduled visits if treatment failure is suspected (all trial participants), stored samples are used for the evaluation of the total HIV-1 DNA and resistance mutations on HIV-1 proviral DNA using the next generation sequencing (NGS). [9] Only in participants who have provided additional consent/assent for the storage of optional samples [10] Only in participants invited (and who have consented/assented) to take part in the MEMS Caps sub-study. Only conducted in Uganda and Kenya. Abbreviations: IMP = investigational medicinal product; C-SSRS = Columbia-suicide severity rating scale; HbA1AC = haemoglobin A1c; HBsAg = hepatitis B surface antigen; HATQoL = HIV/AIDS targeted quality of life; PK = pharmacokinetic; HIV-1 RNA VL = HIV RNA human immunodeficiency Virus-1 ribonucleic acid viral load; C-SSRS = Columbia-suicide severity rating scale; EQD5D = EuroQol-5 dimension; MEMS = medication event monitoring system; CD3+, CD4+, CD8+ = cluster of differentiation 3, -4, -8; VL = viral load; NGS = next generation sequencing; DNA = deoxyribonucleic acid; MEMS = medication event monitoring system

**Table 5:** Characteristics at enrolment into the BREATHER Plus trial.

	Total
Participants randomised Country (%)	470
Kenya	84 (18)
South Africa	32 (7)
Uganda	212 (45)
Zimbabwe	142 (30)
Age category (%), years	
<15	140 (30)
15- < 18	207 (44)
≥18	123 (26)
Median age (IQR), years	16.5 (14.6, 18.1)
Sex (%)	
Male	207 (44)
Female	263 (56)
Ethnicity (%)	
Black	470 (100)
Median BMI (IQR), kg/m2	19.6 (18.0, 21.7)
Mode of HIV-1 acquisition (%)	
Mother to child transmission	454 (97)
Sexual contact	7 (1)
Blood product	1 (0)
Unknown	4 (1)
Other^1^	4 (1)
Viral load (%)^2^	
<50 copies/mL	466 (99)
≥50 copies/mL	4 (1)
WHO HIV clinical stage (%)	
Stage 1	220 (47)
Stage 2	94 (20)
Stage 3	123 (26)
Stage 4	33 (7)
Median time since HIV-1 diagnosis (IQR), years	13.0 (10.6, 14.8)
Median age at ART start (IQR), years	4.1 (1.8, 7.7)
Median time on ART (IQR), years	11.8 (8.6, 14.1)
Median time on dolutegravir-based regimen (IQR), years	2.5 (2.1, 3.2)
Median time on tenofovir disoproxil fumarate/lamivudine/dolutegravir (IQR), years	2.4 (1.8, 2.9)
Median CD4+ T-cell count (IQR), cells/mm3^3^	878 (690, 1119)
Median CD4+/CD8+ T-cell ratio (IQR)^3^	1.3 (1.0, 1.6)
Median haemoglobin (IQR), g/dL^4^	13.3 (12.3, 14.4)
Median eGFR (IQR), mL/min	114 (100, 131)
Median HbA1c (IQR), mmol/mol	36 (33, 39)
Median total cholesterol:HDL cholesterol ratio (IQR)	3.2 (2.8, 3.6)

Abbreviations: BMI=Body Mass Index; HIV-1 = Human Immunodeficiency Virus-1; WHO=World Health Organization; IQR = interquartile range; ART = antiretroviral therapy; CD4 = cluster of differentiation 4; CD8 = cluster of differentiation 8; T-cell = thymus cell; eGFR = estimated glomerular filtration rate; HbA1Ac = haemoglobin A1c; HDL = high-density lipoprotein.

1 Other modes of infection include cross contamination at birth, injection with infected blood, suspected malicious infection, suspected sharing of sharps with infected relative.

2 All participants required viral load <50copies/mL at screening to be eligible for the trial. Enrolment viral loads were measured retrospectively.

3 2 participants missing CD4+ and CD4+/CD8+ T-Cell results.

4 4 participants missing haemoglobin results.

## Data Availability

Data requests are reviewed and data will be shared where possible.

## Abbriviations

3TC: Lamivudine
AE: Adverse event.
ALHIV: Adolescent living with HIV.
AR: Adverse reaction.
ART: Antiretroviral therapy.
ARV: Antiretroviral.
BMI: Body Mass Index.
CD4/--3/--8: cluster of differentiation 4/--3/--8.
CI: Confidence interval.
COVID-19: Coronavirus Disease 2019.
C-SSRS: Columbia-Suicide Severity Rating Scale.
CT: Continuous Therapy.
CTU: Clinical Trials Unit.
DNA: deoxyribonucleic acid.
DTG: Dolutegravir.
eCRF: electronic Case Report Form.
EDCTP: European Developing Countries Clinical Trials Partnership.
eGFR: estimated glomerular filtration rate.
EQD5D: EuroQol-5 Dimension
FDC: Fixed dose combination.
FGD: Focus group discussions.
FTC: Emtricitabine.
GCP: Good Clinical Practice.
GDPR: General Data Protection Regulation.
HATQoL: HIV/AIDS targeted quality of life.
HbA1c: HaemoglobinHemoglobin A1c/glycosylated Haemaglobin
HBSAg: hepatitis B surface antigen.
HDL: high-density lipo-protein.
HIV or HIV-1: Human Immunodeficiency Virus or Human Immunodeficiency Virus-1.
IDI: In-depth interviews.
IDMC: Independent Data Monitoring Committee.
IMP: Investigational medicinal product.
INSTI: Strand transfer Integrase inhibitor.
IQR: interquartile range.
ITT: Intention-to-treat.
JCRC: Joint Clinical Research Centre.
LATA: Long-Acting Treatment in Adolescents: A randomised, open-label, two-arm, 96 week trial in virologically suppressed HIV-1-positive adolescents aged 12–-19 years of age in sub-Saharan Africa.
LDL: Low-density lipoprotein.
MEMS: medication event monitoring system.
MRC: Medical Research Council.
MRC CTU at UCL: Medical Research Council Clinical Trials Unit at University College London.
NGS: Next generation sequencing.
NI: Non-inferiority.
NRTI: Nucleoside reverse transcriptase inhibitor.
NNRTI: Non-nucleoside reverse transcriptase inhibitor
NTD: Neural Tube Defects.
OD: Once daily.
PK: Phar-macokinetic.
PPI: Patient and Public Involvement
RNA: ribonucleic acid.
SAE: Serious adverse event.
SAR: Serious adverse reaction.
SCT: Short cycle therapy.
SD: Standard deviation.
TAF: Tenofovir alafenamide fumarate.
TB: Tuberculosis.
T-cell: Thymus cell
TDF: Tenofovir disoproxil fumarate.
TLD: Fixed dose combination of TDF, lamivudine and dolutegravir.
TNV: Tenofovir.
TMT: Trial Management Team.
TSC: Trial Steering Committee.
UCL: University College London.
VLHIV: Viral load.
WHO: World Health Organization
YTB: Youth Trial Board.
