# Supplementary material for: BREATHER Plus clinical trial design: A randomised non-inferiority trial evaluating the efficacy, safety and acceptability of short cycle (five days on, two days off) dolutegravir/tenofovir-based triple antiretroviral therapy (ART) compared to daily ART in virologically suppressed adolescents living with HIV aged 12 to <20 years in sub-Saharan Africa
Source: Contemp Clin Trials. Author manuscript; Available in PMC 2025 Jul 20. (PMC7617909; doi:10.1016/j.cct.2025.107963)
Supplement: Appendix [file EMS206883-supplement-Appendix.pdf]

Supplementary Materials

BREATHER Plus Trial

Table of Contents

Appendix A: BREATHER Plus Consortium Members; Oversight Committees 3

Appendix B: BREATHER Plus Participant Questionnaires 7

Figure S1. BREATHER Plus Adherence Questionnaire - Randomisation 7
Figure S2. BREATHER Plus Adherence Questionnaire – Continuous Treatment 8
Figure S3. BREATHER Plus Adherence Questionnaire – Weekends Off 9
Figure S4. BREATHER Plus Mood Survey 11
Figure S5. BREATHER Plus Sleep Survey 12

Appendix C: BREATHER Plus Infographic 15

Appendix D: BREATHER Plus Participant Information Sheet Template 26

Appendix E: BREATHER Plus Trial Recruitment 41

Appendix F: BREATHER Plus choice of non-inferiority margin and significance level based on observed confirmed viral rebound risk using the Smooth Away from Expected (SAFE) frontier 42

Appendix G: BREATHER Plus Statistical Analysis Plan 43

Appendix A: BREATHER Plus Consortium Members; Oversight Committees

MRC CTU at UCL: Sarah Pett, Deborah Ford, Margaret Thomason, Alasdair Bamford, Ellen White, Jessica Kirk, Angus Jennings, Helen Ainscough, Rebecca Dodds, Molly Bush, Simona Salomone, Paul Crawley, Alexandra Green, George Pettitt, Jemima Shickle, Stephen Townsend, Hannah Sweeney, Margaret Hook, Sara Peres, Nadine Van Looy, Andy Sum, Diana Gibb, Annabelle South, Elizabeth Chappell, Aoife Nolan

Baylor College of Medicine Children's Foundation, Uganda: Adeodata Kekitiinwa, George Patrick Akabwai, Fredrick Katongole, Naomi Apoto, Resty Babirye Okello, Ronald Nabimba, Muzamil Nsibuka Kisekka, Collins Mujyanama, Gerald Agaba Muzorah, Gideon Ahimbisibwe, Florence Namuli, Rose Namaganda, Lameck Kiyimba, Angella Baita, Rachael Namuddu Kikabi, Henry Balwa, Susan Tukamuhebwa, Maria Benita Aino, Judith Tikabibamu, Anthony Kirabira, Lekku Lawrence, Barbra Nantume, Muhammad Lutalo, Diana Louis Anena, Rose Jacqueline Kadhuba, Patricia Nahirya Ntege, Teddy Kembabazi

Joint Clinical Research Centre, Uganda: Cissy Kityo, Victor Musiime, Shamim Nakabuye, Miriam Namasinga, Peace Hellen Masiga, Roselyn Kabasingo, Claire Nasaazi, Centurio Wandera, Josephine Kobusingye, Disan Mulima, Alex V Musiime, Faith Mbasani, Ezra Lutalo, Brenda Namukwaya Efrance, Odoch Denis, Baliruno David, Ampaire Phionah, Edgar Ayesiga Wandigali, Patrick Ssebunya, Barbara Mukanza, Rashidah Nazzinda, Ritah Mbabazi, Abigail Atwine, Juliet Ankunda, Priscilla Kyobutungi, Elizabeth Kaudha, Sharif Musumba, Jesca Nantale, Mariam Naabalamba, Mangadalen Nansaigi, Diana Antonia Rutebarika, Mary Nannungi, Benson Ouma, Edward

Bagirigomwa, Eddie Rubanga, Josephine Namusanje, Henry Mugerwa, Crispus Katemba, Deborah Kahinju, Eram David Williams, Juliet Ategeka, Ocitti Sunday Paul Labeja, Joan Nantege, Shirat Nakabiri, Jeremy Kirunda, Charles Draleku, Jamilah Namwanga, Joan Nangiya, Baker Rubinga, Rodney Kazooba, Cate Naluyima, Christopher Lwanga, Faith Balmoi Labote, Dridah Nakiboneka, Bibian Nakigozi Nakato, Christine Nambi, Milly Ndigendawani, Jovia Kavuma, Timothy Lubwama, Frank Mbamanya, Nabuuma Haawa Muweesi, Carol Otike

University of Zimbabwe Clinical Research Centre, Zimbabwe: Mutsawashe Bwakura-Dangarembizi, Kusum Nathoo, Hilda Mujuru, Misheck Phiri, Stuart Chitongo, Wendy Mapfumo, Sandra Musarurwa, Taona Mudzviti, Columbus Moyo, Shepherd Mudzingwa, Shirley Mutsai, Vivian Mumbiro, Joy Chimanzi, Makhosonke Ndlovu, Maureen Tshuma, Ruth Nhema, Ennie Chidziva, Cleopatra Langa, Godfrey Musoro, Dorinda Mukura, Edson Marimo, Tinashe Chidemo, Bernadette Malunda, Musunga Tomu, Lynnet Nyakudya, Nicholas Dhibi, Alfed Kateta, Sidney Sithole, Lynette Chibanda, Deka Vincent, Elaine Mwandiwata, Secrecy Gondo, Moses Chitsamatanga, Farai Selina Matimba, Tsitsi Gwenzi, Nathalie Mudzimirema, Allen Matubu, Prosper Sibonile Dube, Pia Ngwaru, Joyline Bhiri, Trust Mukanganiki, Vinie Kouamou, Rufaro Chivaura, Xeshelihle Mhlanga, Hazel Patricia Munyama, Sibusisiwe Weza, Vongai Margaret Chanaiwa, Shamiso Barbara Gwande, Constantine Mutata

Moi University Clinical Research Centre, Kenya: Abraham Siika, Winstone Nyandiko, Cecilia Kiilu, Charity Wambui, Ronald Tonui, Viola Kirui, Caroline Watiri Maina, Florence Kiwunja Njulu, Cornelius Chege, Natalie Sang, Beatrice K. Jakait, Damaris Lagat, Salinah Cherutich, Benedister

Kangogo, Cheruiyot, Brian Odul, Hilda Kaziga, Teri Tarus, Getruth Jerop Kiptoo, Vincent Kipchirchir, Clinton Otieno Achida, Tom Oyoo Osoro, Jairus Kipyego, Violah Samoei, Martha Mokeira Mokaya, Wilson Lokitala Ekiru, Ruth Bosire, Rispher Chepkoech, Festus Kirwa Rugut, Matthew Justus Mutuku, Hellen Jerotich Kiplagat, Millicent Orido, Eslyne Jepkemboi, Vincent Otieno Onyango, Alice Mudogo, Mary Chebet, Joan Chepkurui Kotut, Philemon Kipchirchir Mutai, Eunice Jepkemboi, Julie Chepchumba Choge, Evans Ochieng Ouno, Cecilia Chebet Mengich, Amina Msuo Shali, Solomon Kimurgor Ngetich, Dorcas Akinyi Opot, Shauri Kahindi Gona, Richard Kipsang Kirui, Damaris Jepchumba Kiprutto, Mowlem Pierre, Dorcus Adhiambo, Nicholas Kigen, Sophia Simatwa, Michael Njenga, Cornelius Magut, Kennedy Oyamo, Edna Jepngetich, Gideon Obila, Lydiah Maru, Victor Kibet Langat, Iris Jaluha Sande, Scholastica Njeri Wanjiru, Martha Kaimuri Mwongela

Durban International Clinical Research Site, Enhancing Care Foundation, South Africa:
Moherndran Archary, Sundrapragasen Pillay, Rosie Mngqibisa, Raziya Bobat, Nozibusiso Rejoice Mosia, Precious Sphiwe Cebekhulu, Nombuso Nkosi, Rashina Nundlal, Saajida Rizvi, Jabu Mkhulise, Innocentia Thandokuhle Mncube, Nathaniel Malatjie, Shingirai Chimene, Zethu Mnyandu, Sheleika Singh, Clement Pillay, Tiyara Arumugam, Shauna Francis, Michelle Moodley, Sylvia Nylobo, Fathima Mather, Ida Mundhree, Popi Shabalala, Ntombizonta Ignatia Phewa, Khombisile Muthwa, Simangele Abigail Bengu, Tshamand Fulufhelo Netshitangani, Rayania Premoutt, Kelly Shermon Clark, Mthokozisi Khuzwayo

Neuropsychiatric sub-study: Cissy Kityo, Henry Mugerwa

Social Science sub-study: Janet Seeley, Sarah Bernays, Nothando Ngwenya, Tamlyn Seunanden, Stella Namukwaya, Allen Asiimwe

Health Economics sub-study: Paul Revill, Simon Walker

PK sub-study: David Burger, Angela Colbers, Tom Jacobs, Lisanne Bevers

Youth Trials Board: Magda Conway, Lungile Jafta, Mercy Shibemba, Carlo Giaquinto

Trial Steering Committee Members: Karina Butler, Sam Phiri, Avy Violari, Nathan Ford, Imelda Mahaka, Sarah Pett, Addy Kekitiinwa, Hebron Kalikwani, Priscilla Nakiwala

Data Monitoring Committee Members: Anton Pozniak, Rodolphe Thiebaut, Jane Crawley

Data Monitoring Committee Observers: Justus Ashaba, Lizzie Chappell

Appendix B: BREATHER Plus Participant Questionnaires

Figure S1. BREATHER Plus Adherence Questionnaire - Randomisation

BREATHER

Participant Number: ☐☐☐☐☐☐☐☐☐

3 Letter Code: ☐☐☐

Date of Birth: d d m m m y y y y

Worksheet 30 - Adherence - Randomisation
v1.0 20 Apr 2022

Visit Week:	0 ✓
Visit Date:	d d m m m y y y y

THE FOLLOWING INFORMATION SHOULD BE ENTERED INTO THE 'Adherence - Randomisation' eCRF

We know that it can be difficult taking HIV medicines every day. We are interested in finding out what it is like for you. Being honest about whether or not you take your medicines may help others in the future. Please tick the answer that best describes what is happening to you. Please only think about the HIV medicines you are taking, and not any other medicines. Thank you for your help.

1. How do you take your HIV medicines?

☐ My parent/carer gives me my medicines or supervises me to take them

☐ My parent/carer reminds me or supports me to take them

☐ I take them independently

2. HIV medicines over the last 7 days

During the last 7 days I took:

ALL my pills every day	MOST of my pills	About ONE-HALF of my pills	VERY FEW of my pills	NONE of my pills
☐	☐	☐	☐	☐

3. Did you complete this form on your own?

☐ Yes

☐ No   If No, who else was involved?

e.g. parent, carer, nurse, friend ....

Worksheet 30 – Adherence – Randomisation, v1.0 20 Apr 2022   Page 1 of 1

Figure S2. BREATHER Plus Adherence Questionnaire – Continuous Treatment

BREATHER

Participant Number: ☐☐☐☐☐☐☐☐☐

3 Letter Code: ☐☐☐

Date of Birth: d d m m m y y y y

Worksheet 31 - Adherence – Continuous Treatment
v1.0 20 Apr 2022

Visit Week:	4 (Pilot) ☐	8 ☐	16 ☐	24 ☐	32 ☐	40 ☐
	48 ☐	60 ☐	72 ☐	84 ☐	96 ☐	108 ☐
	120 ☐	132 ☐	144 ☐	156 ☐	168 ☐	180 ☐
	Unscheduled ☐			Other Scheduled Visit ☐ Week Number: ☐☐☐		
Visit Date:	d d m m m y y y y					

THE FOLLOWING INFORMATION SHOULD BE ENTERED INTO THE 'Adherence – Continuous Treatment' eCRF

We know that it can be difficult taking HIV medicines every day. We are interested in finding out what it is like for you. Being honest about whether or not you take your medicines may help others in the future. Please tick the answer that best describes what is happening to you. Please only think about the HIV medicines you are taking, and not any other medicines. Thank you for your help.

1. How do you take your HIV medicines?

☐ My parent/carer gives me my medicines or supervises me to take them

☐ My parent/carer reminds me or supports me to take them

☐ I take them independently

2. HIV medicines over the last 7 days

During the last 7 days I took:

ALL my pills every day	MOST of my pills	About ONE-HALF of my pills	VERY FEW of my pills	NONE of my pills
☐	☐	☐	☐	☐

3a. Some people find they forget to take their medicines at weekends. Have you missed any of your HIV medicines for 2 days in a row at the weekend (Friday/Saturday or Saturday/Sunday) since your last visit?

☐ Yes

☐ No *(Go to Q4)*

3b. If yes, since your last visit for how many weekends have you missed your HIV medicines for 2 days in a row?

(Write number in box) ☐

4. Did you complete this form on your own?

☐ Yes

☐ No If No, who else was involved?

e.g. parent, carer, nurse, friend ....

Worksheet 31 – Adherence – Continuous Treatment, v1.0 20 Apr 2022 Page 1 of 1

Figure S3. BREATHER Plus Adherence Questionnaire – Weekends Off

BREATHER

Participant Number: ☐☐☐☐☐☐☐☐☐

3 Letter Code: ☐☐☐

Date of Birth: d d m m m y y y y

Worksheet 32 - Adherence – Weekends Off
v1.0 20 Apr 2022

Visit Week:	4 ☐	8 ☐	16 ☐	24 ☐	32 ☐	40 ☐
	48 ☐	60 ☐	72 ☐	84 ☐	96 ☐	108 ☐
	120 ☐	132 ☐	144 ☐	156 ☐	168 ☐	180 ☐
	Unscheduled ☐			Other Scheduled Visit ☐	Week Number: ☐☐☐	
Visit Date:	d d m m m y y y y					

THE FOLLOWING INFORMATION SHOULD BE ENTERED INTO THE 'Adherence – Weekends Off' eCRF

We know that it can be difficult taking HIV medicines every day. We are interested in finding out what it is like for you. Being honest about whether or not you take your medicines may help others in the future. Please tick the answer that best describes what is happening to you. Please only think about the HIV medicines you are taking, and not any other medicines. Thank you for your help.

1. How do you take your HIV medicines?

☐ My parent/carer gives me my medicines or supervises me to take them

☐ My parent/carer reminds me or supports me to take them

☐ I take them independently

2. Which days are you supposed to miss your HIV medicines as part of this trial?

(Please tick 2 boxes)

☐ Friday

☐ Saturday

☐ Sunday

3. Since you last clinic visit did you miss all the doses you were supposed to miss?

☐ Yes *(Go to Q5)*

☐ No

4a) If no, how many weekend breaks did you take?

(Write number in box) ☐

4b) Please give reasons for not taking a break: *(tick all that apply)*

☐ Told not to miss weekend doses by doctor

☐ Didn't want to miss weekend doses

☐ Forgot to miss weekend doses

☐ Didn't understand about missing weekend doses

☐ Other

If other, please specify here: ....

BREATHER

Participant Number: ☐☐☐☐☐☐☐☐☐

3 Letter Code: ☐☐☐

Date of Birth: d d / m m m / y y y y

Visit Date: d d / m m m / y y y y

5. Since your last clinic visit, did you always restart your HIV medicine doses immediately after your weekend breaks?

☐ Yes *(Go to Q7)*

☐ No

6. If no, after how many weekends did you not restart your doses immediately after the weekend break?

(Write number in box) ☐

7. During the last 7 days (ignoring breaks allowed at the weekend) I took:

ALL my pills every day	MOST of my pills	About ONE-HALF of my pills	VERY FEW of my pills	NONE of my pills
☐	☐	☐	☐	☐

8. Did you complete this form on your own?

☐ Yes

☐ No If No, who else was involved?

e.g. parent, carer, nurse, friend ....

Figure S4. BREATHER Plus Mood Survey

BREATHER

Participant Number: ☐☐☐☐☐☐☐☐☐

3 Letter Code: ☐☐☐

Date of Birth: d d | m m m | y y y y

Worksheet 33 - Mood survey
V2.0 13 Oct 2022

Visit Week:	0 ☐	8 ☐	24 ☐	48 ☐	72 ☐	96 ☐	144 ☐
	Unscheduled ☐			Other Scheduled Visit ☐ Week Number: ☐☐☐			
Visit Date:	d d | m m m | y y y y						

THE FOLLOWING INFORMATION SHOULD BE ENTERED INTO THE 'Mood survey' eCRF

Guidance: For each the following questions, place an "X" in one of the boxes

Over the last 2 weeks, how often have you been bothered by any of the following problems?	Not at all	Several days *(1 to 7 days over the last 2 weeks)*	More than half the days *(More than 7 days over the last 2 weeks)*	Nearly every day
1) Worrying too much about different things				
2) Not being able to stop or control worrying				
3) Feeling afraid as if something awful or bad might happen				
4) Little interest or pleasure in doing things				
5) Feeling down, depressed or hopeless				
6) Feeling bad about yourself, or that you are a failure or have let yourself or your family down				
7) Difficulty falling asleep				
8) Difficulty staying asleep				
9) Problem waking up too early				
10) Unpleasant dreams that appear almost real or nightmares				

	Very good	Good	Fair	Poor	Very poor
11) Over the past 14 days how would you rate your quality of sleep?					

	Not difficult at all	A little or Somewhat difficult	Very difficult	Extremely difficult
12) If you have been having any of these problems, how difficult have these problems made it for you to do your schoolwork or your work, take care of things at home or get along with other people?				

Worksheet 33 – Mood Survey, v2.0 13 Oct 2022 Page 1 of 1

Figure S5. BREATHER Plus Sleep Survey

BREATHER

Participant Number: ☐☐☐☐☐☐☐☐☐

3 Letter Code: ☐☐☐

Date of Birth: d d / m m m / y y y y

Worksheet 36 - Sleep Survey
v1.0 20 Apr 2022

Visit Week:	24 ☐	48 ☐	Unscheduled ☐	Other Scheduled Visit ☐ Week Number: ☐☐☐
Visit Date:	d d / m m m / y y y y			

THE FOLLOWING INFORMATION SHOULD BE ENTERED INTO THE 'Sleep Survey' eCRF

The following questions refer to the times you get in and out of bed in order to sleep (not including naps).

A BREATHING AND SNORING QUESTIONS

Over the past 4 weeks . . .	Never	1-2 nights per week	3-5 nights per week	6-7 nights per week	Don't know
1. How often were you aware that you have snored?					
2. Were you aware that you had times when you stopped breathing during your sleep?					

B QUESTIONS ABOUT FATIGUE AND TIREDNESS

Over the past 7 days . . .	Not at all	A little bit	Somewhat	Quite a lot	Very much
1. I feel fatigued					
2. I feel weak all over					
3. I feel listless ("washed out")					
4. I feel tired					
5. I have trouble starting things because I am tired					
6. I have trouble finishing things because I am tired					
7. I have energy					
8. I am able to do my usual activities					
9. I need to sleep during the day					
10. I am too tired to eat					
11. I need help doing my usual activities					
12. I am frustrated by being too tired to do the things I want to do					

Worksheet 36 – Sleep Survey, v1.0 20 Apr 2022 Page 1 of 3

BREATHER

Participant Number: ☐☐☐☐☐☐☐☐☐

3 Letter Code: ☐☐☐

Date of Birth: d d m m m y y y y

Visit Date: d d m m m y y y y

	Not at all	A little bit	Somewhat	Quite a lot	Very much
13. I have to limit my social activity because I am tired					

C SLEEPING DIFFICULTY					
Please rate below any difficulties you have had in sleeping over the past 2 weeks					
	None	Mild	Moderate	Severe	Very severe
1. Difficulty falling asleep					
2. Difficulty staying asleep					
3. Problem waking up too early					
	Very satisfied	Moderately satisfied	Satisfied	Dissatisfied	Very dissatisfied
4. How satisfied/dissatisfied are you with your current sleep pattern?					
If you have any sleep problems, please provide further information below. If you do not have sleep problems, please tick 'not at all'					
	Not at all	A little	Somewhat	Much	Very much
5. How noticeable to others do you think your sleep problem is, in terms of impairing the quality of your life?					
6. How worried/distressed are you about your current sleep problem?					
7. To what extent do you consider your sleep problem currently interferes with your daily functioning (e.g. daytime fatigue, mood, ability to function at work/daily chores, concentration, memory, etc.)?					

D QUALITY OF SLEEP					
Over the past 7 days . . .	Not at all	A little bit	Somewhat	Quite a lot	Very much
1. My sleep was restless					

BREATHER

Participant Number: | | | | | | | | | |

3 Letter Code: | | | |

Date of Birth: d d m m m y y y y

Visit Date: d d m m m y y y y

	Not at all	A little bit	Somewhat	Quite a lot	Very much
2. I was satisfied with my sleep					
3. My sleep was refreshing					
4. I had difficulty falling asleep					
	Never	Rarely	Sometimes	Often	Always
5. I had trouble staying asleep					
6. I had trouble sleeping					
7. I got enough sleep					
	Very poor	Poor	Fair	Good	Very good
8. My sleep quality was					

E	SOME FINAL QUESTIONS ABOUT SLEEP!

Over the past 7 days . . .	Not at all	A little bit	Somewhat	Quite a lot	Very much
1. I had a hard time getting things done because I was sleepy					
2. I felt alert when I woke up					
3. I felt tired					
4. I had problems during the day because of poor sleep					
5. I had a hard time concentrating because of poor sleep					
6. I felt irritable because of poor sleep					
7. I was sleepy during the daytime					
8. I had trouble staying awake during the day					

Appendix C: BREATHER Plus Infographic

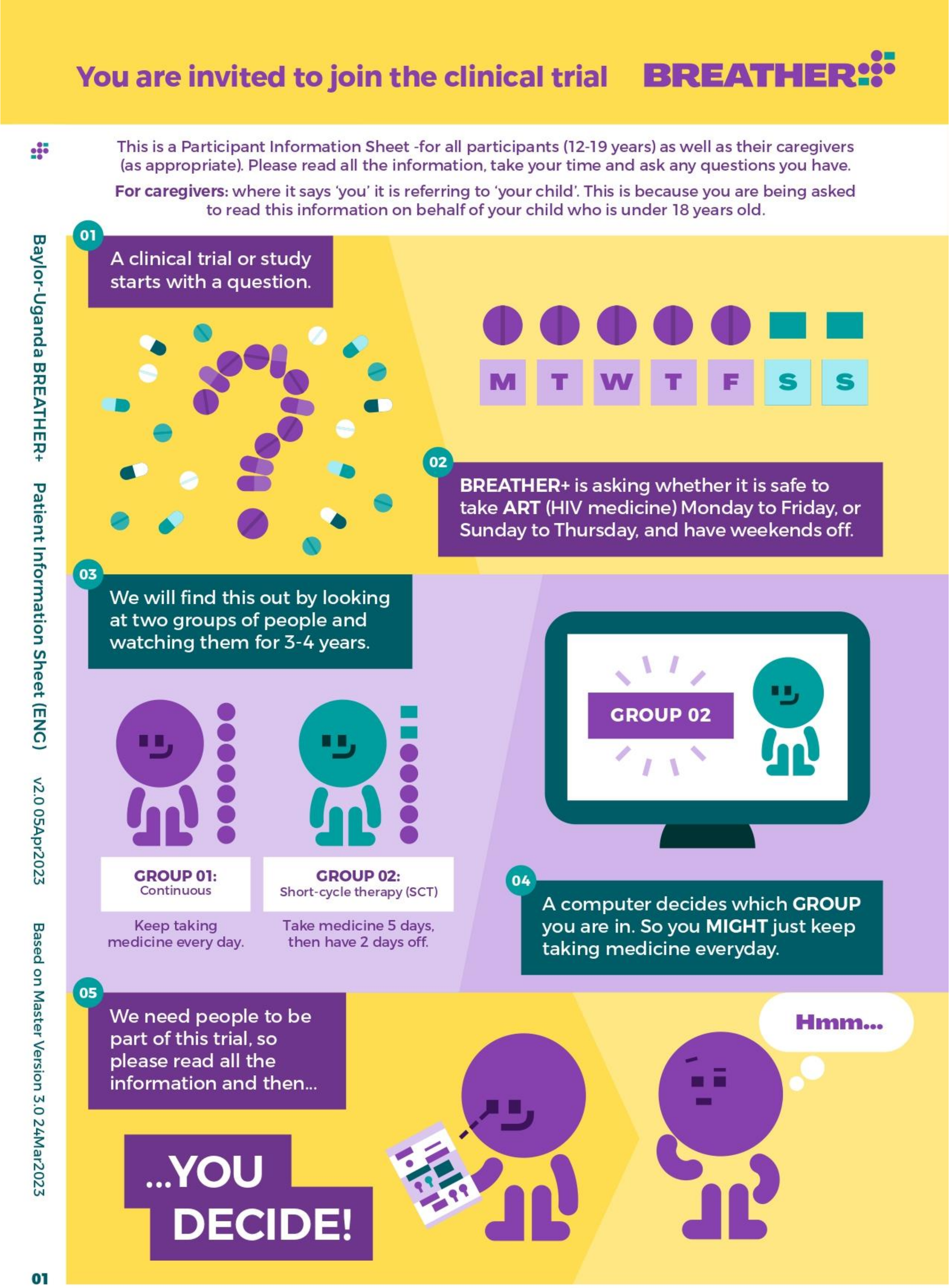

WHO is going to be part of this trial?

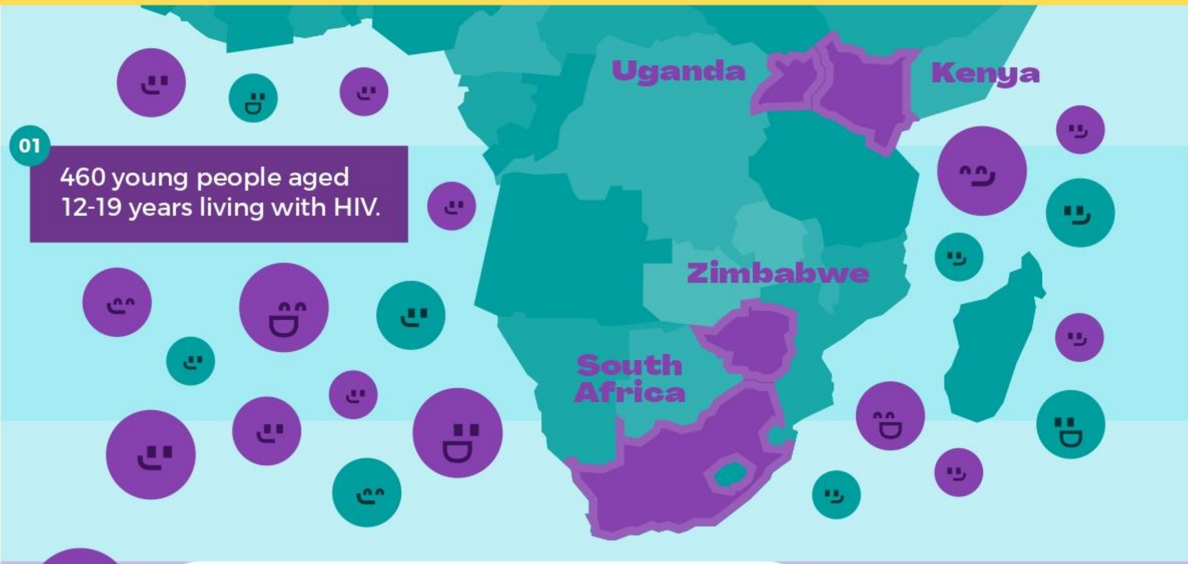

01 460 young people aged 12-19 years living with HIV.

WHY is this trial happening?

02 Some of the HIV medicines now remain in the blood for a long time. We think you might be able to stop the medicine at the weekend but need to run this trial to check it is safe.

WHY me?

03 **There are lots of reasons:**

1) You are interested in the research.

2) You know you have HIV.

3) You've been taking HIV medicine for over a year.

4) The medicines you are taking include dolutegravir, tenofovir and lamivudine /emtricitabine. You probably know this medicine as TLD (or sometimes as TED).

5) You are doing really well on medicine and have an undetectable viral load (which means the virus is completely asleep).

6) You are not pregnant – or planning to become pregnant during the trial – and if you are having sex, you are willing to use effective contraception (your clinic will help with this).

Do I HAVE to?

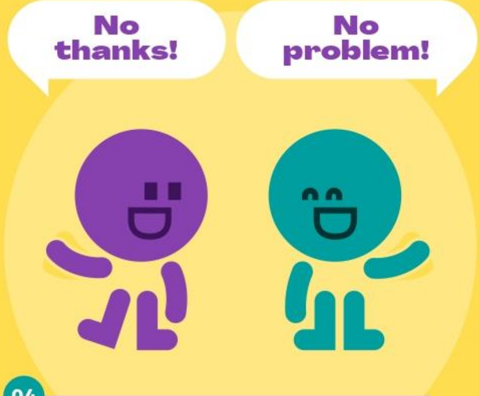

04 You can say **'NO'** now, or leave the trial at any point. It will **NOT** affect the way you get your HIV medicine.

If I say YES, what next?

01

- Aged 12-17? Give your permission by signing an **ASSENT** form. Your caregiver will need to give their permission too, by signing a **CONSENT** form.
- Aged 18-19? Give your permission by signing a **CONSENT** form.
- Have a blood test to make sure it is safe for you to join.
- Answer some questions about whether you are feeling happy or sad.

If you are female and have started your period:

- You will give a urine sample for a pregnancy test.
- The Doctor will talk to you about contraception. If you need this, it'll be free.

02

If you are in the SCT group, we see you after **4 weeks**, then again after **4 weeks**, to check you are OK

Week 1	Week 2	Week 3	Week 4 ✖
Week 5	Week 6	Week 7	Week 8 ✖
Week 9	Week 10	Week 11	Week 12
Week 13	Week 14	Week 15	Week 16 ✖

BREATHER+ pilot group

The first 30 people who join we want to check really closely, so they will come every week for the first **4 weeks**.

Week 1 ✖	Week 2 ✖	Week 3 ✖	Week 4 ✖
Week 5	Week 6	Week 7	Week 8 ✖
Week 9	Week 10	Week 11	Week 12
Week 13	Week 14	Week 15	Week 16 ✖

After this, **EVERYONE** comes to appointments every **8 weeks** in the first year and every **12 weeks** from the second year onwards.

03

The HIV medicine will be provided to you for the BREATHER+ trial.

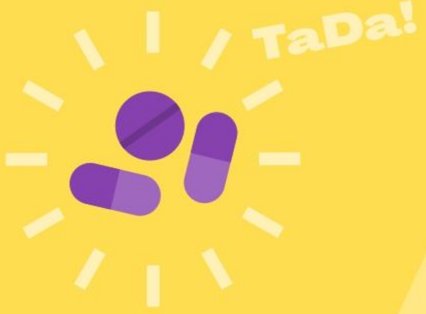

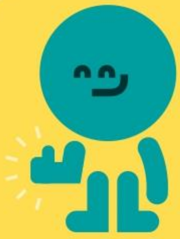

04

You give permission for Blood samples to be taken and stored up to 5 years after the end of BREATHER+.

At each clinic visit: TESTS

01 Blood tests to make sure the medicine is working properly (every 8 weeks in year 1, every 12 weeks in year 2, then every 24 weeks in year 3 onwards), check your immune system, kidneys and liver (every year)

02 At some visits your height, weight, waist, blood pressure and pulse (to make sure your heart is healthy) will be measured. The Doctor will look for signs of any illnesses and ask you how you are doing.

03 Check a urine sample to see if you are pregnant.

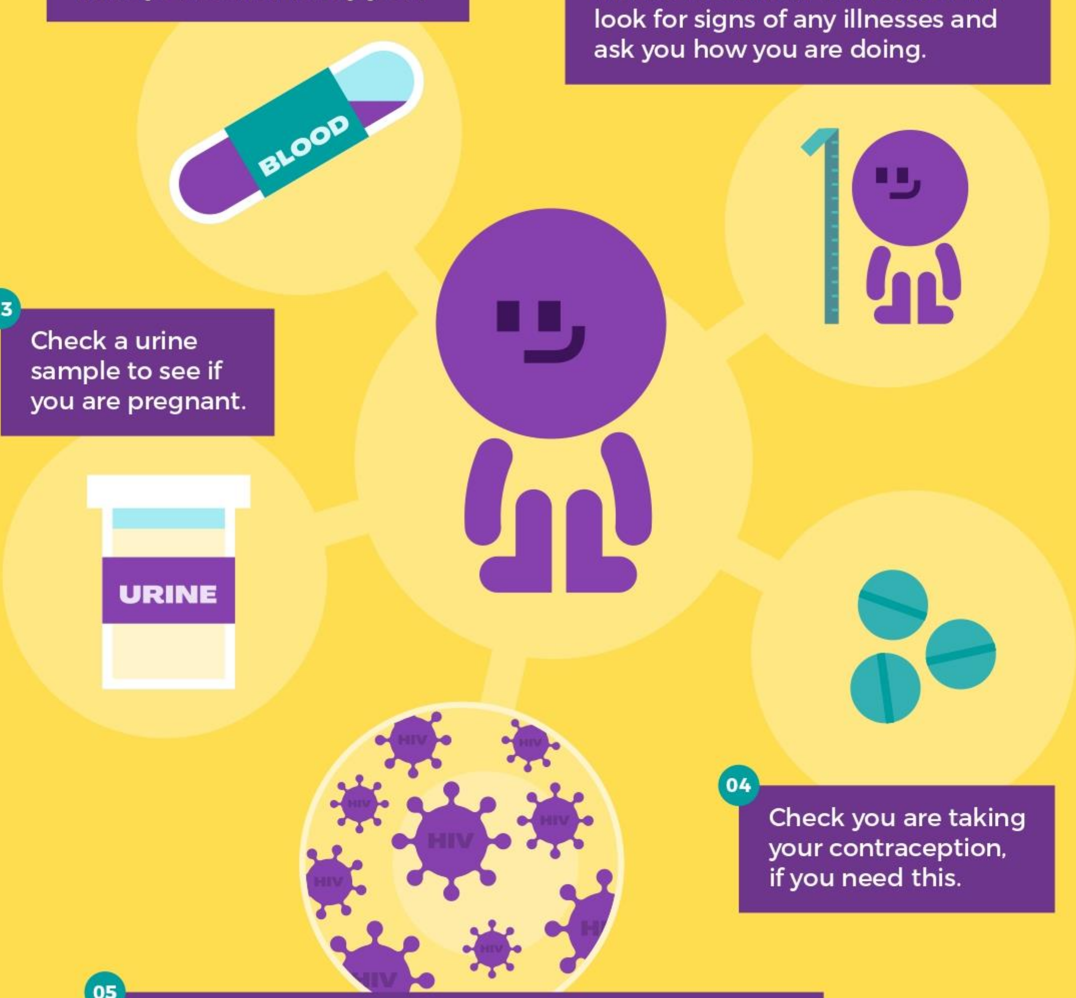

04 Check you are taking your contraception, if you need this.

05 Your clinic will test the HIV virus in your blood, usually once a year, but also if your Doctor has any concerns. If the level of HIV virus gets too high, you will need to come back for a second test at least one week later. If you are in the SCT group, and the second test is still the same, you will be asked to start taking your medicines every day for the rest of BREATHER+. You will not be able to have any more weekend breaks.

Baylor-Uganda BREATHER+ Patient Information Sheet (ENG) v2.0 05Apr2023 Based on Master Version 3.0 24Mar2023

04

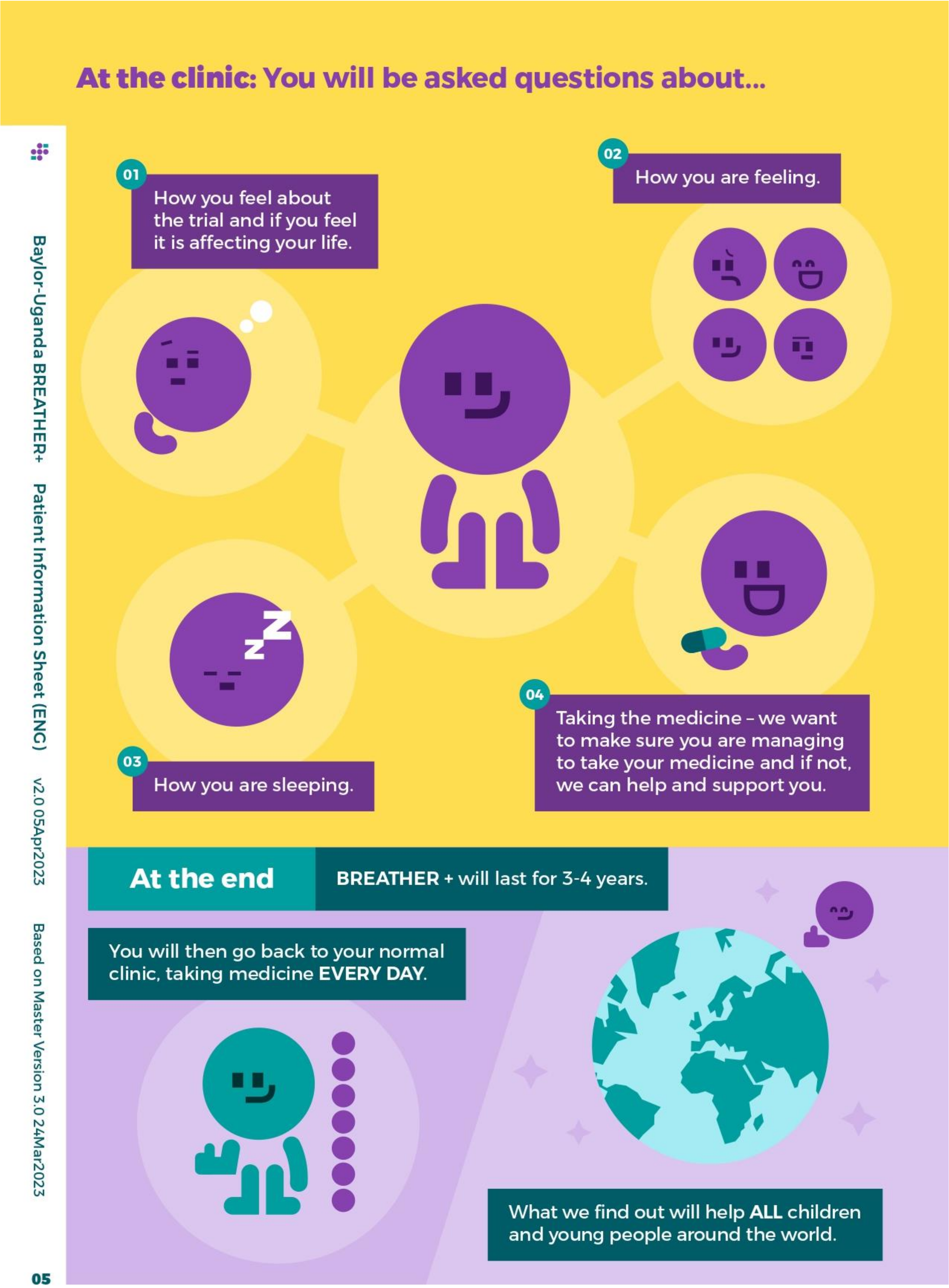

At the clinic: You will be asked questions about...

01 How you feel about the trial and if you feel it is affecting your life.

02 How you are feeling.

03 How you are sleeping.

04 Taking the medicine – we want to make sure you are managing to take your medicine and if not, we can help and support you.

At the end

BREATHER + will last for 3-4 years.

You will then go back to your normal clinic, taking medicine **EVERY DAY**.

What we find out will help **ALL** children and young people around the world.

Risks and side effects

Baylor-Uganda BREATHER+ Patient Information Sheet (ENG) v2.0 05Apr2023 Based on Master Version 3.0 24Mar2023

We face risks everyday. This doesn't mean they are bad, we just need information to understand risks and then decide if we want to take them.

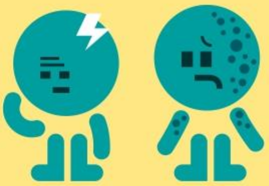

01 Sometimes the HIV medicine can cause a rash, itching, vomiting, stomach pain, weight gain, sleep problems, depression, lack of energy, feeling light-headed or may affect your liver, which is why the Doctor needs to watch you closely.

02 Very rarely, people on dolutegravir get very depressed, and have thoughts about suicide.

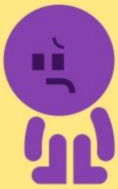

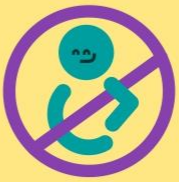

03 Very rarely women who were taking dolutegravir early on in pregnancy, have a baby with a damaged brain or not able to walk. This is called Neural Tube Defect. As more information has become available, we know this risk is rare. In fact, the risk of NTD with dolutegravir is about the same as with other HIV medicines. Although we worry less about this risk now, it is safer not to get pregnant during the trial. See Additional Information.

Always tell your Doctor if you have side effects, feel sad, or just don't feel yourself anymore. **They can help you with side effects.**

Advantages & disadvantages to taking part

Advantages

- You are helping many other young people in the future.
- Making treatment better and easier to take for all young people living with HIV
- You might have freedom from your medicine at weekends.
- You may have fewer side effects.

Disadvantages

- Come to the clinic more often.
- You might find it harder to remember to take your tablets if you are only taking them 5 days a week. Forgetting them can lead to more HIV virus in your body.
- The weekends off may not control the virus as well as taking medicines every day. This could mean your viral load goes up, and you have to change to different medicines to bring it down to undetectable.

06

Additional Information

BREATHER+

It is important you take your time, and read all of this information. If there is anything you don't understand, ask your Doctor or nurse for more information. There is no problem if you don't want to do the trial. You will be looked after in the clinic just the same even if you decide not to take part in **BREATHER+**.

Why this trial is called BREATHER+

This research trial is testing to see whether having a short 2-day break, or a 'breather' from your TLD HIV medicine, just at the weekend is as good as taking TLD every day.

Confidentiality and your information

Your clinic has your contact details and keeps these confidentially, in a locked cabinet. If you join **BREATHER+**, you will be given a trial number and a 3-letter code. ALL study information, blood samples, and forms sent to the Researchers will be marked only with your trial number, 3-letter code and date of birth. Your full name will never be used outside of the clinic.

What happens to your information

The Researchers need the information collected from you for the trial, and some information from your medical records, to be able to answer the question of whether having weekends off TLD HIV medicine is safe. This trial-related information will be sent to Researchers based in Africa and the United Kingdom who designed this trial.

From time to time, the Researchers will ask for your clinic records to be checked against the information that has been sent to them. This is called monitoring. During **BREATHER+** 'Trial Monitors' will visit your clinic to check that the research is being done properly.

Your information after BREATHER+ has finished

Once **BREATHER+** has finished, the Researchers will keep some of your information to check the trial results. They won't know who you are, no names will ever be mentioned in reports they write. They will keep your information for up to 25 years. All information in **BREATHER+** is kept safe and secure.

If you agree to take part, we would like your permission for Researchers to use the information and samples collected in **BREATHER+** for other HIV research. The research purposes your information and samples will be used for will be compatible with BREATHER+ in improving the lives of adolescents living with HIV.

Your information if you leave BREATHER+ early

You can stop being part of **BREATHER+** at any time, without giving a reason, but please talk to your study Doctor or nurse first. They can help with any concerns you may have or provide you with more information.

If you decide to stop taking the study treatment, we would like to keep you in the trial and continue collecting information about you. This is important, because it helps us to make sure that the results of the study are reliable.

If you don't want to do this, that's fine, but we will keep the information and samples that we already have for you. To make sure **BREATHER+** runs properly, you would not be able to change any of the information/samples that have already been collected.

If you stop doing the trial early, it won't change your care at all, you will still be looked after and get your HIV medicines in the clinic.

Finding out more about how your information is used

You can find out more about how the Researchers use your information in the following ways:

1) Asking one of the research team. They can explain it to you.
2) Visiting this website: **www.ctu.mrc.ac.uk/privacy/**
3) Sending an email to **data-protection@ucl.ac.uk** with your questions about how your information is kept safe.
4) Visiting the Sponsor (UCL's) website: **www.ucl.ac.uk/legal-services/privacy/ucl-general-research-participant-privacy-notice**

Days off for the SCT group

If you are in the SCT group, your weekend off HIV medicines can be Friday and Saturday, or Saturday and Sunday. You can decide which is best for you.

More information about what will happen at each clinic visit

You will have blood tests every 8 weeks in the first year in the trial. In year 2, blood tests will be every 12 weeks. In year 3 onwards, blood tests will be every 24 weeks. At the yearly visit, you will have a blood test to check your immune system, blood sugar and levels of HIV virus. Every other year, blood tests will be done to measure your levels of fats, and see how you liver and kidneys are doing. Each visit will take 1-3 hours.

The usual amount of blood taken at each visit, is 1-3 teaspoons. But at 3 visits it will be 9-11 teaspoons because we need to do extra tests. The visits where more blood is taken are at the beginning of the trial, and at the beginning of every year after that while you are in the trial. This might sound like a lot of blood, but it is completely safe to take and you will feel fine.

At the very end of the trial, you will have a 'close-out' visit. At this last visit you will do some of the questionnaires and have a blood test to measure the level of HIV virus. Some of the blood from this test will also be stored for later analysis.

Blood tests you have to have

If you are part of **BREATHER+** you will need to have blood tests to check how much virus you have in your body. Some of these samples we will store for up to 5 years after the end of the trial. As we said before, your name will not appear on any of these samples, they will be coded.

Tests you do not have to have

You will be asked if you are happy to have other blood and urine samples taken. These are optional, so you can say 'no'. These optional samples are taken and stored, and can be used later for research on HIV. They are done three times in the trial. We store these for up to 5 years after the end of the trial because new tests are being developed all the time. If your samples can be kept up to 5 years after the end of the trial, it will give us a better chance of using these new tests.

Travel costs for trial visits

Reasonable travel costs will be paid for all your trial visits. This will include costs for your carer's travel too (if you are 12-17 years of age).

Sending your blood tests to another country

Some of the stored blood tests may be sent to another country to be looked at in a laboratory. This is because the right kind of laboratory isn't always available in every country. These samples will not have your name on them, just your trial number. 3-letter code and date of birth.

If your viral load increases

Your clinic will test the HIV virus in your blood, usually once a year, but also if your Doctor has any concerns. If the amount of HIV virus in your body gets too high, you will need to come back after about a week so we can test you again. If your test results are still too high:

SCT group:

You will need to start taking your medicine every day and stop having weekends off.
Your medical team will help you with this, so do not worry.

Continuous group:

You might have to change your medicines to a different combination.
Your medical team will help you with this, so do not worry.

The medicine being used

The medicine in **BREATHER+** is called **T** (tenofovir), **L** (lamivudine), **D** (dolutegravir).

TLD is used very widely for the treatment of HIV and is a very safe medicine. It is a licensed HIV medicine. This means TLD has had lots of checks to make sure it works well to control the virus, and is safe to use. It is licensed for use in young people and adults. You will have already been on TLD for at least one month before agreeing to come into the trial, so this medicine isn't new for you.

Occasionally instead of TLD you might be on TED. In this combination, instead of lamivudine, there is another medicine called emtricitabine. This medicine works exactly like lamivudine, and is very safe and approved for use in children, adolescents and adults.

Side-effects

All medicines have side effects and you must tell your Doctor or nurse if you feel anything different. This could be physical symptoms (rashes, feeling sick etc.) but also if you don't feel like your normal self.

Kidneys and bones

Two side-effects from this medicine that happen occasionally are that your kidney's don't function properly or that your bones aren't as strong as they should be. This is why we will be doing tests on these throughout **BREATHER+** to make sure you are OK.

If I get side-effects and have to change my medicines, can I continue on the weekends off?

You may have side-effects that means your Doctor thinks you should stop taking TLD. If this happens you won't be able to continue having weekends off. This is because other HIV medicine don't stay in the body as long as TLD. Because of this you will have to take your new HIV medicines every day. Your Doctor will give you more information about this.

Pregnancy

If you are female and accidentally get pregnant, there is a very small chance of your baby having something called 'Neural Tube Defect'. This is when the baby has a damaged brain or may not be able to walk. It is a small risk for every woman – about 1 baby for every 1000 pregnancies, but is a little greater if you take TLD or any other HIV medicines in the first 8 weeks of pregnancy, about 1-2 babies for every 1000 pregnancies. Your Doctor will talk to you more about this.

It is important to understand that the benefits of taking HIV medicine before, during and after pregnancy are much greater than any of the very rare risks of NTD, as it keeps mothers well and prevents babies being infected with HIV.

If you decide you want to get pregnant then you must talk to your Doctor **BEFORE** this happens.

Also the Doctor will talk to you about taking folate. Folate is found in vegetables and other foods, it can also be taken as a tablet. All of us need folate, but it is very important early on in pregnancy. Folate reduces the risk of neural tube defect.

If you do get pregnant during the trial, then we want to make sure you and your baby are OK. To do this we will want to see your baby about a month after they are born, or get information from the clinic looking after your baby about how your baby is doing. This information will also be sent under a code to the pregnancy register in the United States. This pregnancy register is where information about the safety of HIV medicines in pregnancy is reported from all over the world. This information is really important for helping researchers to understand how safe HIV medicines are in pregnancy.

Other things you should know

There is a very small risk from taking blood. People can feel dizzy, and sometimes there is pain when the needle enters through your skin. You can get a bruise or small blood clot where the blood has been taken. Very rarely, you can get an infection where the needle was inserted.

Your blood pressure will be measured using a band on your upper arm. The band tightens, and squeezes your arm for a few minutes. It can be a little uncomfortable but only for a minute or two.

Because one group of **BREATHER+**, the SCT group, will take HIV medicine 5 days a week and not 7, the virus may come back. If it does, then there is risk you could pass it on if you have sex without a condom.

Sub-studies

Most clinical trials have 'sub-studies'. These are different small pieces of research that answer different questions from the main one the study is asking. In **BREATHER+** the sub-studies are:

Happiness

During **BREATHER+** you will be asked to fill in confidential questionnaires about:

- When you are taking your medication
- Whether you are happy with being part of the study
- Whether you have had any thoughts about harming yourself or even tried to hurt yourself, because you were very unhappy
- How it is affecting you and your life (in a good and bad way)

If these show you are feeling unhappy, worried or having problems with sleeping, we may ask for you to speak with a specialist (a psychologist, or psychiatrist) to help you with how you are feeling.

We may also ask you to be involved in a 'neuropsychiatric sub-study'. There is a separate information sheet and assent/consent form for this.

Neuropsychiatric means looking at the brain and nervous system. This will help us understand why you are feeling the way you are and what is worrying you. It will also mean that we can help you.

We may also ask you to take part in this sub-study if you have none of these feelings, so we can check we are not missing anything else.

Being part of this sub-study means you will be asked to fill in different questionnaires. Your Doctor may also think it's a good idea to speak with a specialist (a psychologist, or psychiatrist) to help you with how you are feeling if the questionnaires in the sub-study suggest you are feeling very worried or unhappy.

Taking medicine (Adherence)

We need 200 young people from **BREATHER+** for another sub-study where they will use **MEMS** (Medication Event Monitoring Systems) caps.
A MEMS cap is an electronic device fixed to your medicine bottles which records when it is opened (the time and date). If you agree to take part in this sub-study you will be given a MEMS cap to fit to your medicine bottle for 6 months either in the first or second year of the trial. Your Doctor or nurse will explain how it works and show you how to use it.

How you are feeling

We want to find out more about how young people living with HIV feel about taking medicine, being part of this clinical trial and life with HIV. A few young people will be invited to be part of a sub-study run by Social Scientists. These are people specially trained to ask questions and really listen to your answers. If you want to be part of this, let your nurse or Doctor know. There is a separate information sheet and assent/consent form for this.

When BREATHER+ finishes

After **BREATHER+** has ended everyone will go back to taking HIV medicine every day. You and your Doctor will continue to make decisions about your treatment. If stopping HIV medicines at the weekend is found to be a safe and a good way of treating young people living with HIV you might be able to have a treatment break at weekends.

Additional Information

BREATHER+

How will you find out the results

When **BREATHER+** has finished, there will be some different ways to find out what was learnt:

1) We will tell all the participants, and their carers (as appropriate), the results. We may do this in a short information sheet, or through community meetings held at your clinic.
2) The Researchers will publish a summary of the results on the website of the MRC CTU at UCL **www.ctu.mrc.ac.uk**
3) The results will be published in a medical journal, so that other Doctors can see them and learn from them. You can ask your Doctor for a copy of any publication. Your identity and any personal details will be kept confidential. No named information about you will be published in any report of **BREATHER+**.

Keeping you safe

BREATHER+ has been looked at by an independent group of people called a 'Research Ethics Committee'. Their job is to check the study does not hurt anyone. They have said this study is safe.

A second group called the 'Independent Data Monitoring Committee' will meet regularly during the trial and decide if the trial should keep going or whether it is not safe and should stop.

Where to report worries or concerns

If you have any concerns about the study, the way you have been treated in the trial or the way it has been run please talk to your study Doctor or nurse.

Investigators:

Dr. Adeodata Rukyalekere Kekitiinwa,
Principal Investigator : 0772462686

Dr. George Patrick Akabwai,
Co-Investigator : 0772982863

The hospital running **BREATHER+**

Baylor College of Medicine
Children's Foundation, Uganda
Block 5 Mulago Hospital
PO Box 72052, Clock Tower
Kampala - Uganda
Tel: +256-(0)-417-119100/Tel: +256-(0)-312-119100
Email: admin@baylor-uganda.org

If you are still unhappy, or if you wish to complain, please use the normal clinic complaints process.

If you as the participant, are harmed by taking part in **BREATHER+**, or if you are harmed because of someone's negligence, then you may be able to take legal action. If this happens you can contact:

Professor Grace Ndeezi,
Chairperson Joint Clinical Research Centre
– Research Ethics Committee (JCRC-REC)

Plot 101, Lubowa Off Entebbe Road; P.O Box 10005, Kampala.
Tel: 0772453191, 04147723000.
Email: irb@jcrc.org.ug; gndeezi@gmail.com

BREATHER+ is funded by the European and Developing Countries Clinical Trials Partnership. This is a partnership between researchers in Europe, the United Kingdom and in Africa aimed at improving the health of people living in Africa.

BREATHER+ is sponsored by University College London (UCL), which is a university based in the UK. This means UCL has overall responsibility for the conduct of the study.

Questions?

Thank you for taking the time to think about being part of **BREATHER+**. Please ask any questions and let us know if there are things that you do not understand or would like more information about.

This information sheet has been developed by members of different Youth Trials Boards. These are groups of young people living with HIV who are working to give children and young people a voice in clinical trials and research. They represent different countries around the world.

Appendix D: BREATHER Plus Participant Information Sheet Template

To be presented on local headed paper   To be presented on local headed paper

MASTER TEMPLATE: BREATHER+ MAIN TRIAL INFORMATION SHEET – PARENT/GUARDIAN/CARER OF PARTICIPANTS AGED 12-17 YEARS

BREATHER

Information Sheet Version & Date: X.0 DD-MMM-YYYY
Based on Master PISCF Version & Date: 3.0 24-Mar-2023
Protocol Version: 3.0, 24-Mar-2023

1. Introduction

Dear Parent/Legal Guardian,

Your child is invited to participate in a research study with an investigational medicine. Before you agree to let your child take part in this research, you must read this information sheet, as it contains important information to help you decide whether it is in your child's best interests to participate.

You are encouraged to ask as many questions as needed in order to ensure that you understand the study procedures, including possible risks and benefits. If you have any questions that are not properly explained or answered in this information leaflet, please feel free to ask a study staff member to give you more information. You are welcome to take this document home with you and to discuss your child's possible participation in the study with your family and friends.

The study has been approved by [insert ethics and regulatory bodies] for compliance with medical and ethical standards. In addition, the study will be conducted according to the 2013 Declaration of Helsinki, Guidelines for good practice in the conduct of clinical trials and Ethics in Health Research: Principles, Processes and Structures (2015) which deals with your rights as a research participant and which guide the study doctor (investigator) in health research involving human participants.

The doctor in charge of this study is [insert site PI]. The study team will be paid by the study funder, the European and Developing Countries Clinical Trials Partnership EDCTP, to conduct this study.

2. Your child's rights as a participant

Your child's participation in this study is voluntary. You may choose for your child not to be in the study or to leave the study at any time by telling the study doctor. If you decide to not let your child participate in the study or to withdraw your child's consent, your child will not lose any benefits to which they are otherwise entitled.

The study doctor has the right to withdraw your child from the study if it is considered to be in your child's best interests, in which event, the reasons will be given to you.

3. Study summary

Some HIV medicines can remain in the blood for a long time. We think young people might be able to stop their HIV medicine at the weekend but need to run this study to check it is safe. BREATHER+ is asking whether it is safe to take HIV medicine Monday to Friday, or Sunday to Thursday, and have weekends off. We will find this out by following two groups of young people for 3-4 years.
Group 1: Continuous - keep taking HIV medicine every day
Group 2: Short-Cycle Therapy (SCT) – take HIV medicine 5 days, then have 2 days off

If your child is in the SCT group, their weekend off HIV medicines can be Friday and Saturday, or Saturday and Sunday. You and your child can decide which is best for them.

Approximately 460 participants aged 12-19 years living with HIV in South Africa, Uganda, Kenya and Zimbabwe will participate in BREATHER+. At [insert site name], around [insert recruitment target] participants will take part in BREATHER+.

The HIV medicine will be provided to your child for the BREATHER+ study. The HIV medicine in BREATHER+ is called T (tenofovir), L (lamivudine), D (dolutegravir). TLD is used very widely for the treatment of HIV and is a very safe medicine. It is a licensed HIV medicine for use in young people and adults. This means TLD has had lots of checks to make sure it works well to control the virus, and is safe to use. Your child will have already been on TLD for at least one month before agreeing to come into the BREATHER+ study, so this medicine isn't new for them. Occasionally instead of TLD your child might be on TED. In this combination, instead of lamivudine, there is another medicine called emtricitabine. This medicine works exactly like lamivudine, and is very safe and approved for use in children, adolescents and adults.

4. Study design

A computer decides which of the two groups your child is in. So your child MIGHT just keep taking medicine everyday.

5. Why has my child being invited to take part?

There are lots of reasons why your child is being invited to take part in BREATHER+ including:
1) They are interested in the research.
2) They know they have HIV.
3) They've been taking HIV medicine for over a year.
4) The medicines they are taking include dolutegravir, tenofovir and lamivudine/emtricitabine. You probably know this medicine as TLD (or sometimes as TED).
5) They are doing really well on medicine and have an undetectable viral load (which means the virus is completely asleep).
6) They are not pregnant – or planning to become pregnant during the study – and if they are having sex, they are willing to use effective contraception (your child's clinic will help with this).

6. What does my child have to do if they are in this study?

If you decide for your child to take part in BREATHER+ you will need to:

- Give your permission by signing a consent form

Your child will need to:

- Give their permission by signing an assent form
- Have a blood test to make sure it is safe for them to join
- Answer some questions about whether they are feeling happy or sad

If your child is female and has started their period:

- They will give a urine sample for a pregnancy test
- The doctor will talk to your child about contraception. If they need this, it'll be free

The first 30 people who join BREATHER+ will be part of the pilot study, and will come to clinic every week for the first 4 weeks.

If your child is in the SCT group, we see them after 4 weeks, then again after another 4 weeks, to check they are OK. If your child is in the continuous group, we see them after 8 weeks to check they are OK. After this, EVERYONE comes to appointments every 8 weeks in the first year, then every 12 weeks from year 2 onwards.

At clinic visits your child will have:

- Blood tests to make sure the HIV medicine is working properly (every 8 weeks in year 1, every 12 weeks in year 2, then every 24 weeks in year 3 onwards)
- At the yearly visit, your child will have a blood test to check their immune system, blood sugar and levels of HIV virus
- Every other year, blood tests will be done to measure your child's levels of fats, and see how their liver and kidneys are doing
- At some visits your child's height, weight, waist, blood pressure and pulse (to make sure their heart is healthy) will be measured. The doctor will look for signs of any illnesses and ask your child how they are doing
- Questionnaires about how your child feels about the study and if they feel it is affecting their life
- Questionnaires about how they are feeling
- Questionnaires about how they are sleeping
- Questionnaires about taking the HIV medicine – we want to make sure they are managing to take their HIV medicine and if not, we can help and support your child
- Your child's clinic will test the HIV virus in their blood, usually once a year, but also if your child's doctor has any concerns. If the level of HIV virus gets too high, your child will need to come back for a second test at least one week later. If your child is in the SCT group, and the second test is still the same, your child will be asked to start taking their medicines every day for the rest of BREATHER+. Your child will not be able to have any more weekend breaks
- Female participants: have a urine sample collected to see if they are pregnant
- Female participants: check they are taking their contraception, if they need this

Each visit will take 1-3 hours.

The tables below describes the study procedures that will be done at each visit.

STUDY WEEK NUMBER	SCRN	RAND	0	1	2	3	4	4	8	16	24	32	40	48	60	72	84	96	THEN EVERY
**Clinical Assessments and dispensing requirements**				PILOT				SCT only											
Informed consent/assent	•																		
Review eligibility	•	•	•																
Demographics	•																		
Complete HIV viral load history and ART history	•																		
Complete medical history and HIV-1 infection confirmed		•																	
Contraception check	•	•	•	•	•	•	•	•	•	•	•	•	•	•	•	•	•	•	12 weeks
Clinical assessment		•					•	•	•	•	•	•	•	•	•	•	•	•	12 weeks
Symptoms check		•					•	•	•	•	•	•	•	•	•	•	•	•	12 weeks
Vital signs			•						•		•			•				•	48 weeks and close-out visit
Concomitant medication check and ART regimen review			•	•	•	•	•	•	•	•	•	•	•	•	•	•	•	•	12 weeks
Adherence assessment with pill count			•	•	•	•	•	•	•	•	•	•	•	•	•	•	•	•	12 weeks
AE and health utilisation assessment			•	•	•	•	•	•	•	•	•	•	•	•	•	•	•	•	12 weeks
Dispense HIV medicines			•				•	•	•	•	•	•	•	•	•	•	•	•	12 weeks

STUDY WEEK NUMBER	SCRN	RAND	0	1	2	3	4	4	8	16	24	32	40	48	60	72	84	96	THEN EVERY
**Laboratory Assessments**				PILOT				SCT only											
Biochemistry			•															•	
Lipids			•															•	
HbA1c			•											•				•	
Haematology			•											•				•	
T cell bloods			•											•				•	48 weeks
Real-time Plasma HIV-1 RNA	•			•	•	•	(•)							•				•	48 weeks and at close-out visit
Urine pregnancy test	•	•							•	•	•	•	•	•	•	•	•	•	12 weeks

STUDY WEEK NUMBER	SCRN	RAND	0	1	2	3	4	4	8	16	24	32	40	48	60	72	84	96	THEN EVERY
**Other Assessments**				PILOT				SCT only											
Adherence questionnaire			•				•	•	•	•	•	•	•	•	•	•	•	•	12 weeks
Columbia-Suicide Severity Rating Scale (C-SSRS)	•	•							•		•			•		•		•	48 weeks, and at close-out visit
Mood Survey			•						•		•			•		•		•	48 weeks, and at close-out visit
Acceptability and wellbeing questionnaire (HATQoL)			•								•			•				•	48 weeks and at close-out visit
Quality of Life questionnaire (EQ-5D)			•								•			•				•	And at close-out visit
**Storage Samples**				PILOT				SCT only											
Mandatory Plasma (10mL) stored for HIV RNA and/or potential resistance testing			•				•	•	•	•	•	•	•	•	•	•	•	•	24 weeks and at close-out visit
Plasma 10mL (for bone, renal & inflammatory biomarkers) – optional/additional samples			•											•				•	
Urine for renal biomarkers – optional/additional samples			•															•	
**Estimated total blood draw per visit (in mL)**	5		45	5	5	5	15	10	10	10	10	10	10	40	0	10	0	50	

The usual amount of blood taken at each visit, is 1-3 teaspoons. But at 3 visits it will be 9-11 teaspoons because we need to do extra tests. The visits where more blood is taken are at the beginning of the study, and at the beginning of every year after that while your child is in the study. This might sound like a lot of blood, but it is completely safe to take and your child will feel fine.

At the very end of the study, your child will have a 'close-out' visit. At this last visit your child will do some of the questionnaires and have a blood test to measure the level of HIV virus. Some of the blood from this test will also be stored for later analysis.

If your child's viral load increases
Your child's clinic will test the HIV virus in their blood, usually once a year, but also if their doctor has any concerns. If the amount of HIV virus in your child's body gets too high, they will need to come back after about a week so we can test them again. If your child's test results are still too high:

SCT group: Your child will need to start taking their HIV medicine every day and stop having weekends off. Your child's medical team will help them with this, so do not worry.
Continuous group: Your child might have to change their HIV medicines to a different combination. Your child's medical team will help them with this, so do not worry.

BREATHER+ will last for 3-4 years. After BREATHER+ has ended everyone will go back to taking HIV medicine every day. You and your child's doctor will continue to make decisions about their treatment. If stopping HIV medicines at the weekend is found to be a safe and a good way of treating young people living with HIV your child might be able to have a treatment break at weekends. What we find out will help ALL children and young people living with HIV around the world.

For this study to be successful, it is important that your child co-operates fully with the study doctor and staff and follow their instructions precisely. Please inform the study doctor of all the medicines that your child is currently taking.

7. What will happen to your child's samples?

If your child is part of BREATHER+ they will need to have blood tests to check how much HIV virus they have in their body. Some of these samples we will store for up to 5 years after the end of the study. Your child's name will not appear on any of these samples, they will be coded.

You will be asked if you are happy for your child to have other blood and urine samples taken. These are optional, so you can say 'no'. These optional samples are taken and stored, and can be used later for research on HIV. They are done three times in the study. We store these for up to 5 years after the end of the study because new tests are being developed all the time. If your child's samples can be kept up to 5 years after the end of the study, it will give us a better chance of using these new tests.

Some of the stored blood tests may be sent to another country to be looked at in a laboratory. This is because the right kind of laboratory isn't always available in every country. These samples will not have your child's name on them, just their study number, 3-letter code and date of birth.

8. What are the possible side effects?

We face risks every day. This doesn't mean they are bad, we just need information to understand risks and then decide if we want to take them. All medicines have side effects and your child must tell their doctor or nurse if they feel anything different. This could be physical symptoms (rashes, feeling sick etc.) but also if they don't feel like their normal self.

Sometimes the HIV medicine can cause a rash, itching, vomiting, stomach pain, weight gain, sleep problem, depression, lack of energy, feeling light-headed or may affect the liver, which is why the doctor needs to watch your child closely.

Very rarely, people on dolutegravir get very depressed, and have thoughts about suicide. Very rarely, women who were taking dolutegravir early on in pregnancy, have a baby with a damaged brain or not able to walk. This is called Neural Tube Defect. As more information has become available, we know the risk is rare. In fact, the risk of NTD with dolutegravir is about the same as with other HIV medicines. Although we worry less about this risk now, it is safer to not get pregnant during the study.

Two side-effects from this medicine that happen occasionally are that your child's kidneys don't function properly or that their bones aren't as strong as they should be. This is why we will be doing tests on these throughout BREATHER+ to make sure your child is OK.

Your child may have side-effects that means their doctor thinks they should stop taking TLD. If this happens your child won't be able to continue having weekends off. This is because other HIV medicine don't stay in the body as long as TLD. Because of this your child will have to take their new HIV medicines every day. Your child's doctor will give you more information about this.

Always tell your child's doctor if your child has side effects, feels sad, or just doesn't feel themselves anymore. They can help your child with side-effects.

Because one group of BREATHER+, the SCT group, will take HIV medicine 5 days a week and not 7, the virus may come back. If it does, then there is risk your child could pass it on if they have sex without a condom.

9. What are the possible risks of study procedures?

There is a very small risk from taking blood. People can feel dizzy, and sometimes there is pain when the needle enters through the skin. Your child can get a bruise or small blood clot where the blood has been taken. Very rarely, people can get an infection where the needle was inserted.

Your child's blood pressure will be measured using a band on their upper arm. The band tightens, and squeezes the arm for a few minutes. It can be a little uncomfortable but only for a minute or two.

You or your child must inform the study doctor immediately if they experience any negative effects, complications or injuries while taking part in the study. Because the medicines they take is investigational there may be risks with the use of the medicines that are not currently known. You will be notified of any new significant findings that may affect your willingness for your child to continue in the study.

10. Risks to participants of childbearing potential

If your child is female and gets pregnant during the study, there is a very small chance of their baby having something called 'Neural Tube Defect'. This is when the baby has a damaged brain or may not be able to walk. It is a small risk for every woman – about 1 baby for every 1000 pregnancies, but is a little greater if they take TLD or any other HIV medicines in the first 8 weeks of pregnancy, about 1-2 babies for every 1000 pregnancies. Your child's doctor will talk to you more about this.

It is important to understand that the benefits of taking HIV medicine before, during and after pregnancy are much greater than any of the very rare risks of NTD, as it keeps mothers well, and prevents babies being infected with HIV. If your child decides they want to get pregnant then they must talk to their doctor BEFORE this happens.

Also the doctor will talk to your child about taking folate. Folate is found in vegetables and other foods, it can also be taken as a tablet. All of us need folate, but it is very important early on in pregnancy. Folate reduces the risk of neural tube defect.

If your child does get pregnant during the study, then we want to make sure your child and their baby are OK. To do this we will want to see your child's baby about a month after they are born, or get information from the clinic looking after your child's baby about how the baby is doing. This information will also be sent under a code to the pregnancy register in the United States where information about the safety of HIV medicines in pregnancy is reported from all over the world. This information is really important for helping researchers to understand how safe HIV medicines are in pregnancy.

If your child is able to have children and they are sexually active, they must agree to use highly effective contraception (methods which have an expected failure rate less than 1% per year) during participation in the study. BREATHER+ considers the following contraceptive methods to be acceptable;

- Injectable contraceptives (such as Depo Provera and Nur-isterate)
- Implantable contraceptives (such as Evra)
- Oral contraceptives (such as Femodene, Yasmin, Yaz Plus, Nordette, etc)
- Intrauterine contraceptives (such as Nova-T, Mirena)

No other contraception will be allowed.

If your child becomes pregnant during the study, you must contact the study doctor immediately, who will advise you on whether your child should continue with the study medicines. With your permission, the pregnancy will be followed-up. The study doctor will advise you about your child and their baby's future medical care.

11. What are the possible advantages and disadvantages of being in the study?

Possible advantages of being part of BREATHER+ include:

- Your child is helping many other young people with HIV in the future.
- Making treatment better and easier to take for all young people living with HIV.
- Your child might have freedom from their HIV medicine at weekends.
- Your child may have fewer side effects.

Possible disadvantages of being part of BREATHER+ include:

- Coming to the clinic more often.
- Your child might find it harder to remember to take their HIV medicine if they are only taking them 5 days a week. Forgetting them can lead to more HIV virus in your child's body.
- The weekends off may not control the virus as well as taking HIV medicines every day. This could mean your child's viral load goes up, and they have to change to different HIV medicines to bring it down to undetectable.

12. Alternative treatments

If you decide not to let your child take part in this study, your child will still receive the best current care and HIV medicine, from your usual doctor; this may or may not include the study medicine.

13. Compensation in the event of a study-related injury

The BREATHER+ study has an insurance policy taken out by University College London (the Sponsor of the study) which will cover any bodily injury your child suffers as a result of taking part in the study.

The insurer will pay for all reasonable medical costs required to treat your child's bodily injury, in accordance with the Good Clinical Practice Guidelines. You may request a copy of these guidelines from the study doctor.

The insurer will pay without your child having to prove that the research was responsible for their bodily injury.

The insurer will **not** pay for harm if, during the study your child:

- Uses medicines or substances that are not allowed
- Does not follow the study doctor's instructions
- Does not tell the study doctor that they have a bad side effect from the study medicine
- Suffers an injury arising from negligence on your child's part or does not take reasonable care of themselves and their study medicine.

If your child is harmed and the insurer pays for the necessary medical costs, usually your child will be asked to accept that insurance payment as full settlement of the claim for medical costs. However, accepting this offer of insurance cover does not mean you give up your right to make a separate claim for other losses based on negligence.

If your child belongs to a private medical scheme, you should inform the scheme that your child is participating in a research study.

14. What about confidentiality?

Your child's clinic has their contact details and keeps these confidentially, in a locked cabinet. If your child joins BREATHER+, they will be given a study number and a 3-letter code. ALL study information, blood samples, and forms sent to the researchers will be marked only with their study number, 3-letter code and date of birth. Your child's full name will never be used outside of the clinic.

The researchers need the information collected from your child for the study, and some information from your child's medical records, to be able to answer the question of whether having weekends off TLD HIV medicine is safe. This study-related information will be sent to researchers based in Africa and the United Kingdom who designed this study.

From time to time, the researchers will ask for your child's clinic records to be checked against the information that has been sent to them. This is called monitoring. During BREATHER+ study monitors will visit your child's clinic to check that the research is being done properly.

Once BREATHER+ has finished, the researchers will keep some of your child's information to check the study results. They won't know who your child is, no names will ever be mentioned in reports they write. They will keep your child's information for up to 25 years. All information in BREATHER+ is kept safe and secure. If you agree for your child to take part, we would like your permission for researchers to use the information and samples collected in BREATHER+ for other HIV research. The research purposes your information and samples will be used for will be compatible with BREATHER+ in improving the lives of adolescents living with HIV.

Your child can stop being part of BREATHER+ at any time, without giving a reason, but please talk to your child's study doctor or nurse first. They can help with any concerns you may have or provide you with more information.

If your child decides to stop taking the study medicines, we would like to keep them in the study and continue collecting information about them. This is important, because it helps us to make sure that the results of the study are reliable.

If your child doesn't want to do this, that's fine, but we will keep the information and samples that we already have for them. To make sure BREATHER+ runs properly, you would not be able to change any of the information/samples that have already been collected.

If your child stops doing the study early, it won't change their care at all, they will still be looked after and get their HIV medicines in the clinic.

You can find out more about how the researchers use your child's information in the following ways:

1) Asking the study doctor or nurse. They can explain it to you

2) Visiting this website www.ctu.mrc.ac.uk/privacy/

3) Sending an email to data-protection@ucl.ac.uk with your questions about how your child's information is kept safe

4) Visiting the Sponsor (UCL's) website: https://www.ucl.ac.uk/legal-services/privacy/ucl-general-research-participant-privacy-notice

Study information can be accessed by:

- Study monitors, auditors and contractors who may work for the Sponsor or its affiliates/authorized representatives, who check that the study is being performed correctly and that the information collected about your child is accurate;

- Research Ethics Committees that have approved this study and will ensure that your child's rights and well-being are safeguarded;
- National regulatory authorities involved in keeping research safe for participants.

The sponsor or representatives may use the study data sent to them for the following purposes:
- To see if the study medicines works and is safe.
- To compare the study medicines to other medicines.
- For other activities relating to the study medicines.
- To investigate how the body responds to HIV and its treatment

You have the right to ask the study doctor about the data being collected on your child and to see your child's personal health information and, if applicable, ask for corrections.

If you should withdraw your child from the study, data collected prior to your child's withdrawal may still be processed along with other data collected as part of the study. No new information will be collected for the study database unless you specifically consent to that. However, the law does require that any side effects your child may suffer are documented and reported. To complete the study findings, your child's long-term health status may also be obtained (unless you object). You have the right to require that any previously retained records are destroyed.

15. Keeping your child safe

BREATHER+ has been looked at by an independent group of people called a 'Research Ethics Committee'. Their job is to check the study does not hurt anyone. They have said this study is safe.

A second group called the 'Independent Data Monitoring Committee' will meet regularly during the study and decide if the study should keep going or whether it is not safe and should stop.

16. Will my child be compensated for taking part in the study?

You will not receive payment for participating in the BREATHER+ study.

Any study-related costs, such as study medicines, tests, examinations and procedures specified in the protocol will be paid for by the funder. Neither you, your child, your medical scheme nor your healthcare provider will be responsible for these expenses.

The Sponsor has made provision to reimburse you and your child for out-of-pocket expenses such as travelling to and from the study site and to compensate your child for other miscellaneous costs, such as time spent at the site and inconvenience, because of study participation. You and your child will receive a minimum amount of [insert amount in local currency] per visit.

17. Study results

When BREATHER+ has finished, there will be some different ways to find out what was learnt:
1. We will tell all the participants, and their carers (as appropriate), the results. We may do this in a short information sheet, or through community meetings held at your child's clinic.

2. The researchers will publish a summary of the results on the website of the MRC CTU at UCL (http://www.ctu.mrc.ac.uk/).
3. The results will be published in a medical journal, so that other doctors can see them and learn from them. You can ask your child's doctor for a copy of any publication. Your child's identity and any personal details will be kept confidential. No named information about your child will be published in any report of BREATHER+.

18. Sub-studies

Most studies have 'sub-studies'. These are different small pieces of research that answer different questions from the main one the study is asking. In BREATHER+ the sub-studies are:

A. Happiness

During BREATHER+ your child will be asked to fill in confidential questionnaires about:

- When they are taking their HIV medicines
- Whether they are happy with being part of the study
- Whether they have had any thoughts about harming themselves or even tried to hurt themselves, because they were very unhappy
- How the study is affecting them and their life (in a good and bad way)

If these questionnaires show your child is feeling unhappy, worried or having problems with sleeping, we may ask for your child to speak with a specialist (a psychologist, or psychiatrist) to help them with how they are feeling.

We may also ask your child to be involved in a 'neuropsychiatric sub-study'. There is a separate information sheet and assent/consent form for this. Neuropsychiatric means looking at the brain and nervous system. This will help us understand why your child is feeling the way they are and what is worrying them. It will also mean that we can help your child. We may also ask for your child to take part in this sub-study if they have none of these feelings, so we can check we are not missing anything else.

Being part of this sub-study means your child will be asked to fill in different questionnaires. Your child's doctor may also think it's a good idea to speak with a specialist (a psychologist, or psychiatrist) to help your child with how they are feeling if the questionnaires in the sub-study suggest your child is feeling very worried or unhappy.

B. How your child is feeling

We want to find out more about how young people living with HIV feel about taking HIV medicine, being part of this study and living with HIV. A few young people will be invited to be part of a sub-study run by Social Scientists. These are people specially trained to ask questions and really listen to your child's answers. If you want your child to be part of this, let your child's nurse or doctor know. There is a separate information sheet and assent/consent form for this.

19. What do I do if I have questions or problems?

If you have any concerns about the study, the way your child has been treated in the study or the way it has been run please talk to your child's study doctor or nurse.

Investigators:

[Insert site specific BREATHER+ details of 2 trial site investigators (including names, role and phone numbers)]

The hospital/site running BREATHER+
[Insert site specific BREATHER+ hospital name and address (full address)]

If you are still unhappy, or if you wish to complain, please use the normal clinic complaints process.

If your child is harmed by taking part in BREATHER+, or if your child is harmed because of someone's negligence, then you may be able to take legal action. If this happens you can contact:
[Insert country/site details of the local REC – name, address, phone number and email address]

BREATHER+ is funded by the European and Developing Countries Clinical Trials Partnership (EDCTP). This is a partnership between researchers in Europe and in Africa aimed at improving the health of people living in Africa.

BREATHER+ is sponsored by University College London (UCL), which is a university based in the UK. This means UCL has overall responsibility for the conduct of the study.

Thank you for taking the time to think about your child being part of BREATHER+. Please ask any questions and let us know if there are things that you do not understand or would like more information about.

MASTER TEMPLATE: BREATHER+ MAIN TRIAL CONSENT FORM – PARENT/GUARDIAN/CARER OF PARTICIPANTS AGED 12-17 YEARS

[To be presented on local headed paper]

[Remove text box and insert relevant details/logos here]

BREATHER+

BREATHER+: A randomised open label 2-arm, 96-week trial evaluating the efficacy, safety and acceptability of short cycle (five days on, two days off) dolutegravir/tenofovir-based triple antiretroviral therapy (ART) compared to daily dolutegravir/tenofovir-based triple ART in virologically suppressed HIV-infected adolescents aged 12 to 19 years of age in sub-Saharan Africa

Version 3.0, 24-Mar-2023

Centre Name & Number: ....

Study Number: ....

Study 3-Letter Code: ....

Name of Researcher: ....

	Initial boxes to agree
1. I confirm that I have read and understood the information sheet for the **BREATHER+** research study **Version 3.0 24-Mar-2023** and have been given a copy to keep.	
2. I confirm that I have had the opportunity to ask questions about BREATHER+ and the use of my child's personal data, and I have received satisfactory answers to all of my questions.	
3. I understand that the results of my child's screening tests might show that they are not eligible to take part in the BREATHER+ study.	
4. I understand that sections of any of my child's medical notes may be looked at by responsible representative involved in the running of the study, regulatory authorities, or the drug companies providing the HIV medicines, where it is relevant to my child's taking part in this research. I give permission for these individuals to have access to my child's records, but understand that my child's confidentiality will be maintained.	
5. I understand that my child may not benefit directly by participating in this study but that the research may help people with HIV in the future.	
6. I understand that my child's participation in all aspects of this study is voluntary and that they are free to withdraw from the study at any time, without giving any reason and without their medical care or legal rights being affected.	
7. I agree to allow blood samples to be taken from my child and analysed for the purpose of the BREATHER+ trial.	
8. I agree that my child can provide blood samples which will be stored for testing the amount of HIV virus and resistance testing. I give permission for these samples to be tested in a laboratory in a different country.	

	Initial boxes to agree
9. I agree for my child to take part in the above study.	
10. I understand that even if I give my permission for my child to participate, and they don't give their agreement (assent) then they cannot participate in the BREATHER+ trial.	

The following statements are optional:

	Initial boxes to agree
I give permission for my child's stored samples to be made available for research outside of the BREATHER+ trial protocol, where the samples would be stored appropriately and the research approved separately. I understand that some of these projects may be carried out by researchers other than the MRC CTU at UCL. I understand that the results of these research projects are unlikely to have any implications for my child personally. *(If you do not wish to give this permission, do not put your initials in the box – your child can still take part in the trial).*	
I give permission for my child's data to be made available for research outside of the BREATHER+ trial protocol, where the data would be stored appropriately and the research approved separately. I understand that some of these projects may be carried out by researchers other than the MRC CTU at UCL. I understand that the results of these research projects are unlikely to have any implications for my child personally. *(If you do not wish to give this permission, do not put your initials in the box – your child can still take part in the trial).*	
I give permission for additional blood and urine samples to be taken and stored from my child. *(If you do not wish to give this permission, do not put your initials in the box – your child can still take part in the trial).*	
I agree for my child to take part in the adherence substudy using MEMs caps. *(If you do not wish to give this permission, do not put your initials in the box – your child can still take part in the trial).*	

If you agree that your child may take part, please complete the section below:

Parent/Guardian/Carer signature (or thumbprint)	Name of Parent/Guardian/Carer	Date
Witness's signature (if thumbprint used above)	Name of witness	Date

To be completed by the person taking informed consent:

I have provided the BREATHER+ study information to the participant's Parent/Guardian/Carer in full and answered all his/her questions. To the best of my knowledge, he/she understands the purpose, interventions, risks and benefits of this study and willingly agrees for his/her child to be enrolled into BREATHER+.		
Signature of person conducting the informed consent process	Name of person conducting the informed consent process	Date

I would/would not *(please circle as appropriate)* like my child's family Doctor to be notified about their participation in this trial. [Country specific, delete if not applicable]

Signature of Parent/Guardian/Carer:________________________________Date:_______________

Name of Family Doctor: ____

Contact address of Family Doctor: ____

IMPORTANT: One signed original to be kept in the BREATHER+ trial file by the research team
One signed copy to be given to the participant's Parent/Guardian/Carer
One signed copy to be kept in the clinic file

Appendix E: BREATHER Plus Trial Recruitment

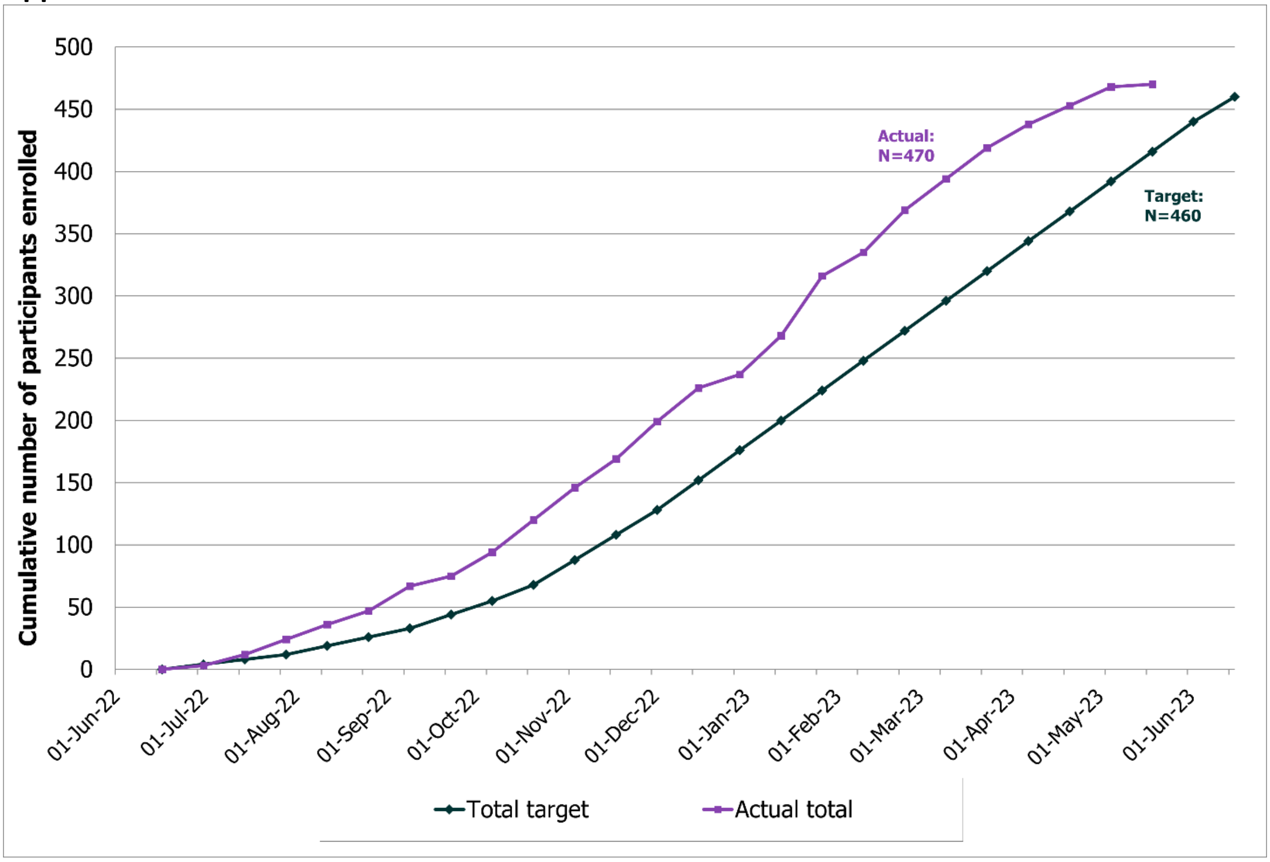

Appendix F: BREATHER Plus choice of non-inferiority margin and significance level based on observed confirmed viral rebound risk using the Smooth Away from Expected (SAFE) frontier

Observed confirmed viral rebound risk (P0)*	1%	2%	3%	4%	5%	6%	7%	8%	9%	10%	11%	12%	13%	14%	15%
**NI Margin**	5.0%	5.8%	6.5%	7.3%	8.0%	8.9%	9.5%	9.9%	10.0%	10.0%	**10.0%**	10.0%	10.0%	10.0%	10.0%
**Significance level**	0.5%	0.5%	0.5%	0.5%	0.5%	0.5%	0.5%	0.5%	2.5%	2.5%	**2.5%**	2.50%	2.50%	2.50%	2.50%
**Power**	95.8%	89.1%	84.4%	81.4%	80.4%	81.1%	82.2%	83.3%	84.6%	85.0%	**86.3%**	86.6%	85.7%	84.0%	82.0%
**Type 1 error**	2.50%	2.28%	2.36%	2.53%	2.67%	2.76%	2.86%	2.74%	2.69%	2.71%	**2.69%**	2.66%	2.64%	2.63%	2.61%
**P (change margin)****	100%	100%	100%	99.9%	99.0%	95.0%	84.8%	67.8%	47.5%	29.0%	**15.5%**	12.1%	6.1%	2.8%	1.2%

Footnote: The column in bold and shaded corresponds to the sample size calculation assumption made at the design stage.

*The choice of non-inferiority margin and significance level will depend on the observed confirmed viral rebound risk. The power, type 1 error and probability of changing margin depend on the true control event risk.

**The probability of changing the margin is the probability that, for a given true control event risk, the observed control event risk will be lower than 9%, hence leading to using a non-inferiority margin in the analysis different from the originally planned 10%.

Abbreviations: NI= non-inferiority

Appendix G: BREATHER Plus Statistical Analysis Plan

BREATHER Plus

A randomised open-label 2-arm, 96-week trial evaluating the efficacy, safety and acceptability of short cycle (five days on, two days off) dolutegravir/tenofovir-based triple antiretroviral therapy (ART) compared to daily dolutegravir/tenofovir-based triple ART in virologically suppressed HIV-infected adolescents aged 12 to 19 years of age in sub-Saharan Africa

ISRCTN: ISRCTN5058577

STATISTICAL ANALYSIS PLAN

Version 2.0

SAP version and approvals

**Version Number and Date:** 2.0 (17 June 2024)<br>Supersedes version: 1.4 (13 June 2024)<br>**In relation to protocol version:** 3.0			
**Author**	**Position**	**Signature**	**Date**
Angus Jennings	Delegated Statistician, MRC CTU at UCL		
**Reviewed by**			
Dr Deborah Ford	Trial Statistician (Senior Statistician), MRC CTU at UCL		
Professor Rodolphe Thiébaut	Independent Senior Statistician (reviewer), University of Bordeaux		
**Approved by**			
A/Professor Adeodata Kekitiinwa-Rukyalekere	Trial Chief Investigator, Baylor College of Medicine Children's Foundation-Uganda		
Professor Sarah Pett	Project Lead at MRC CTU at UCL, MRC CTU at UCL		

SAP revision history

Version	Date	Author	Comments
0.1	18 March 2020	Deborah Ford	Brief analysis plan described in protocol version 2.0.
0.2	03 December 2021	Ellen White	First draft
0.3	07 December 2021	Ellen White	Incorporating feedback on D3 SAP
0.4	08 December 2021	Ellen White	Incorporating feedback from Debbie Ford
IDMC Meeting (Discussion of protocol and SAP prior to enrolment opening) - 16 December 2021			
0.5	11 February 2022	Ellen White	Incorporating details on modified snapshot algorithm and non-inferiority margin in line with D3 SAP
0.6	25 February 2022	Ellen White	Incorporated feedback from Debbie Ford and Man Chan on modified snapshot algorithm
0.7	03 March 2022	Ellen White	Incorporated feedback from Debbie Ford and Sarah Pett on modified snapshot algorithm<br>Incorporated additional non-inferiority margin information from Matteo Quartagno
0.8	07 March 2022	Ellen White	Incorporated feedback from Debbie Ford and Sarah Pett
0.9	04 April 2022	Ellen White	Incorporate feedback from Rodolphe Thiébaut on D3 SAP and at first IDMC meeting.<br>Updated resistance and neuropsychiatric analysis sections.
1.0	06 April 2022	Ellen White	Up-versioned SAP
Pilot Study IDMC Review (via email) – 23 December 2022			
IDMC Meeting – 14 February 2023			
IDMC Meeting – 04 October 2024			
1.1	22 May 2024	Angus Jennings/Debbie Ford	Full specification of the MI approach to be used<br>Include switch SCT-CT for viral rebound in primary endpoint<br>SAE status when site and clinical reviewer disagree clarified<br>Clarified that models will only adjust for randomisation stratification factors assuming there are sufficient participants in strata levels<br>Safety analyses excluding spontaneous and induced abortions included
1.2	30 May 2024	Angus Jennings	Incorporated feedback from Debbie Ford<br>Updated censoring dates from W102 to 'W102 – 1'
1.3	06 June 2024	Angus Jennings	Finalising Primary Analysis sensitivity analyses

1.4	13 June 2024	Angus Jennings	Modified section on Sensitivity Analysis (10.9) following Ian White's review of D3 SAP and amended one censoring date to account for any clinic visit with no VL measurement.
2.0	17 June 2024	Angus Jennings	Up-versioned SAP
IDMC Meeting – 27 June 2024			

Table of Contents

1. IMPORTANCE AND RATIONALE 7
2. TRIAL OBJECTIVES AND HYPOTHESIS 7
2.1 Primary objective ________ 7
2.2 Hypothesis ________ 7
3. ESTIMAND FOR PRIMARY ENDPOINT 7
4. TRIAL DESIGN 8
4.1 Study design ________ 8
4.2 Study Population ________ 9
4.3 Selection of Participants ________ 9
4.3.1 Participant Inclusion Criteria 9
4.3.2 Participant Exclusion Criteria 9
5. OUTCOME MEASURES 10
5.1 Primary outcome ________ 10
5.2 Secondary outcomes ________ 10
5.2.1 Efficacy 10
5.2.2 Safety 10
5.2.3 Patient-reported outcomes 10
6. SAMPLE SIZE CALCULATIONS 10
6.1 The SAFE Non-Inferiority frontier ________ 11
7. ANALYSIS POPULATIONS FOR ANALYSIS 14
7.1 Intention-to-treat population and analysis ________ 14
7.2 Per protocol population and analysis ________ 14
8. STRATA AND COVARIATES 14
8.1 Stratification variables ________ 14
9. DATA HANDLING AND DERIVATION 15
9.1 Definition of baseline ________ 15
9.2 Definition of Follow-up time ________ 15
9.3 Definition of nominal week for clinical and laboratory measurements ________ 15
9.4 Handling missing data ________ 16
9.4.1 HIV-1 RNA data 16
9.4.2 Immunology data 16
9.5 Handling of viral load data ________ 16
9.6 Handling adverse event data ________ 16
9.7 Other definitions ________ 17
9.8 Data quality ________ 17
10. STATISTICAL ANALYSES 17
10.1 General principles ________ 17

10.2 Analysis of continuous variables 18
10.3 Analysis of adverse event data 18
10.4 Analysis of participant and parent/carer questionnaire data 18
10.5 Analysis details 19
10.5.1 Enrolment and eligibility 19
10.5.2 Baseline characteristics 19
10.6 Follow up and availability of HIV-1 RNA measurements 21
10.6.1 Follow up 21
10.6.2 Availability of HIV-1 RNA measurements 21
10.7 Treatment adherence and protocol deviations 21
10.7.1 Use of antiretroviral therapy after randomisation 21
10.7.2 Protocol deviations 22
10.8 Primary outcome analysis by week 96 22
10.9 Sensitivity analyses for the primary outcome 25
10.10 Subgroup analysis for the primary outcome 25
10.11 Seconday analysis of primary endpoint 25
10.12 Secondary outcome analyses 26
10.12.1 Efficacy 26
10.12.2 Safety 27
10.12.3 Patient-reported outcome measures (questionnaires) 28
10.12.4 Other outcomes 29
10.13 Testing multiple secondary outcomes 29
11. SUBSTUDIES 30
12. TIMING OF INTERIM ANALYSIS 30
12.1 Pilot study 30
12.2 Interim Analyses 30
13. TIMING OF FINAL ANALYSIS 30
14. APPENDICES 31
14.1 Appendix 1 – Handling Missing Data with Multiple Imputation 31
14.1.1. General procedure 31
14.1.2. Handling viral load measurement below the limit of detection 34
14.1.3. Combining bootstrapping with multiple imputation 35
14.1.4. Using imputed missing viral load measurements after loss to follow-up and death 35
14.2 Appendix 2 - Proposed rules for requesting retrospective HIV-1 RNA testing from stored plasma samples 37
14.3 Appendix 3 - Considerations/assumptions on HIV-1 RNA data for analysis 38
14.4 Appendix 4 - Details of modified FDA snapshot algorithm 39
REFERENCES 43

1. IMPORTANCE AND RATIONALE

The BREATHER PLUS trial will evaluate the virological efficacy, safety, acceptability and Quality of Life of DTG-based Short-cycle Therapy (SCT) with weekends off compared with Continuous Therapy (CT) with a DTG-based ART regimen. The backbone drugs will consist of tenofovir either as the TAF or TDF formulations partnered with either 3TC or FTC. Importantly for generalisability to low- and middle-income settings, the trial will be conducted using standard-of-care real-time viral load monitoring as recommended by the World Health Organization (currently six-monthly and annual in the adolescent population in sub-Saharan Africa); with additional plasma samples taken for safety monitoring by the Independent Data Monitoring Committee (IDMC) but not returned to doctors/patients.

2. TRIAL OBJECTIVES AND HYPOTHESIS

The overall aim of the BREATHER Plus trial is to assess whether DTG-based SCT with a tenofovir and lamivudine/emtricitabine backbone will provide non-inferior sustained virological suppression compared to continuous dolutegravir-based ART with a tenofovir and lamivudine/emtricitabine backbone.

2.1 PRIMARY OBJECTIVE

To assess whether DTG-based SCT with a tenofovir and lamivudine/emtricitabine backbone is non-inferior to continuous dolutegravir-based ART with a tenofovir and lamivudine/emtricitabine backbone in terms of virological suppression.

2.2 HYPOTHESIS

Dolutegravir-based SCT with a tenofovir and lamivudine/emtricitabine backbone will provide non-inferior sustained virological suppression compared to continuous dolutegravir-based ART with a tenofovir and lamivudine/emtricitabine backbone over 96 weeks.

3. ESTIMAND FOR PRIMARY ENDPOINT

**Treatments**	The comparison is between the SCT group and the CT group (control), as described in section 4.
**Population**	The population of interest is HIV-1 infected adolescents aged 12 to 19 years in Kenya, South Africa, Uganda, and Zimbabwe that meet the inclusion/exclusion criteria as defined in section 4.3.
**Endpoint**	Proportion of children with confirmed viral rebound, defined as the first of 2 consecutive HIV-1 RNA≥50 c/mL at any time up to the 96-week assessment (as defined in section 5.1)
**Population-level summary measure**	Difference in proportions (SCT - CT)
**Intercurrent events**	
**Any treatment modification including:**<br>• change in any ART component;<br>• ART dose modification;<br>• ART discontinuation;<br>• Return to continuous ART in the SCT group	Treatment policy

**Missed doses of treatment**	Treatment policy
**Died**	Hypothetical

4. TRIAL DESIGN

4.1 STUDY DESIGN

The BREATHER Plus trial is an open-label, randomised (1:1), multicentre, non-inferiority trial in HIV-infected, non-pregnant, non-breastfeeding adolescents aged 12 to 19 years of age, virologically-suppressed for at least one year, without any history of treatment failure, on 3-drug combination antiretroviral (ART) consisting of dolutegravir with a 2-drug NRTI backbone consisting of tenofovir and lamivudine/emtricitabine for at least 1 month. All participants will be recruited in sub-Saharan Africa.[1]

A total of 460 adolescents will be randomised to DTG-based short-cycle therapy with weekends off (SCT) or continuous therapy with a DTG-based ART regimen (CT) (230 in each group). Randomisation will be a permuted block-randomisation approach, stratified by centre and mode of infection (horizontal or vertical). Visits are at screening, week 0 (randomisation), 4 (SCT only), 8, 16, 24, 32, 40, 48, 60, 72, 84, 96, and then every 12 weeks until the last participant reaches their 96 week visit.

Figure 4.1 Trial Entry, Randomisation and Treatment

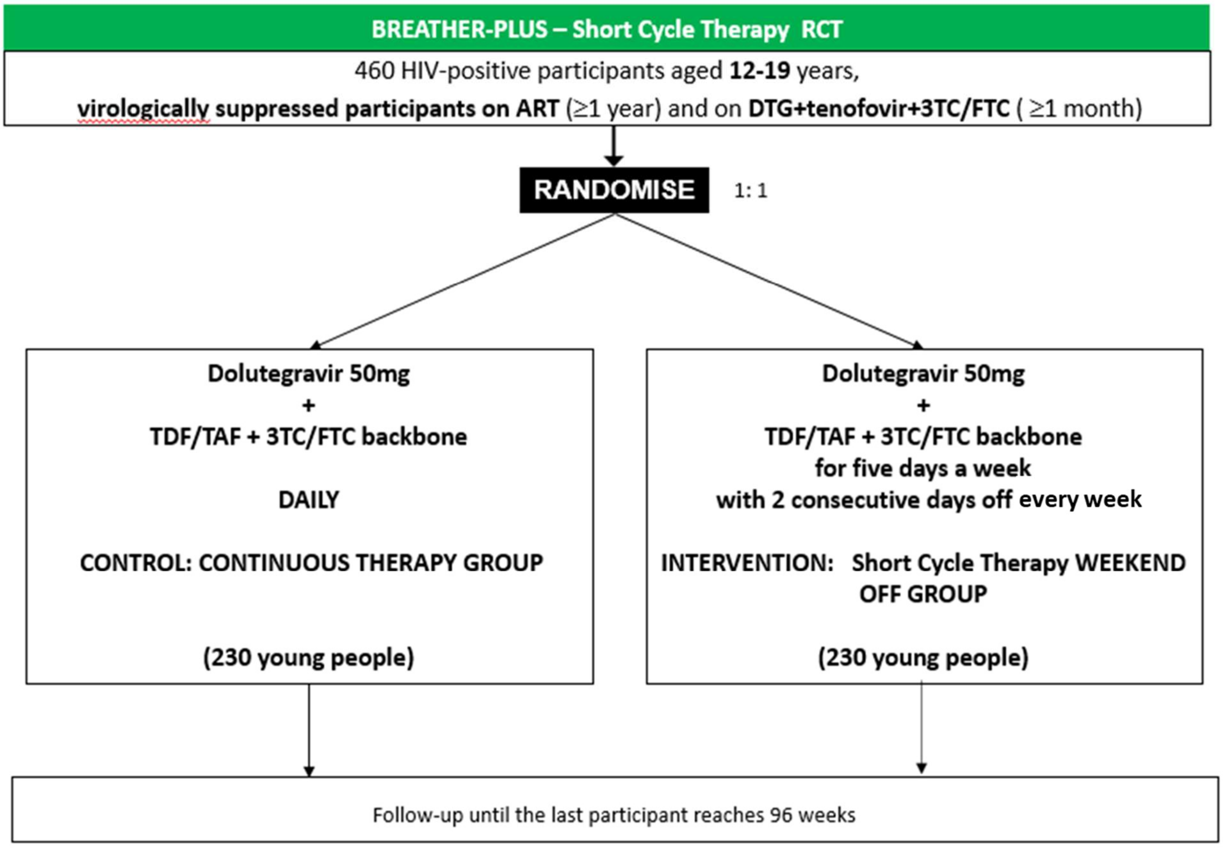

Visits at screening, week 0 (randomisation), 4[Ω], 8, 16, 24, 32, 40, 48, 60, 72, 84, 96, and then every 12 weeks until the last participant reaches their 96 week visit.

[Ω]SCT group only

---

[1] Full details of trial documents (including the Protocol) are stored in the BREATHER Plus eTMF

4.2 STUDY POPULATION

HIV-1 infected adolescents aged 12 to 19 years will be recruited from sites in Kenya, South Africa, Uganda, and Zimbabwe.

4.3 SELECTION OF PARTICIPANTS

There will be **no exceptions** to eligibility requirements at the time of randomisation. Questions about eligibility criteria must be addressed prior to attempting to randomise the participant. The eligibility criteria are the standards used to ensure that only medically appropriate patients are considered for this study. Patients not meeting the criteria should not join the study. For the safety of the participants, as well as to ensure that the results of this study can be useful for making treatment decisions regarding other patients with similar diseases, it is important that no exceptions be made to these criteria for admission to the trial.

Participants will be considered eligible for enrolment in this trial if they fulfil all the inclusion criteria and none of the exclusion criteria as defined below.

4.3.1 Participant Inclusion Criteria

1. HIV-1-infected
2. Aged 12 to 19 years
3. Aware of HIV status
4. On ART for ≥1 year, with no previous regimen change for treatment failure
5. On ART consisting of DTG, tenofovir and lamivudine/emtricitabine for ≥1 month prior to screening
6. Virologically suppressed with all HIV-1 RNA viral loads <50 c/mL[a] in the last 12 months up to and including screening. Additionally there must be one result <50 c/mL[a] at least 12 months prior to screening and the viral load at trial screening must be <50 c/mL
7. Girls who are sexually active must be willing to adhere to highly effective methods of contraception[b]
8. Written informed consent provided by participant (if aged 18 to 19 years) and/or carer/legal guardian (if participant aged 12 to 17 years) as appropriate
9. Written informed assent in participants aged 12 to 17 years

4.3.2 Participant Exclusion Criteria

1. Females who are pregnant or breastfeeding
2. Females who plan to become pregnant during the trial follow-up or are unwilling to use a highly effective method of contraception[b] for the duration of the trial if sexually active
3. Moderate or High risk score on the Columbia-Suicide Severity Rating Scale
4. On treatment for any active TB
5. Contraindication to continued receipt of dolutegravir or any formulation of tenofovir, lamivudine/emtricitabine
6. Underlying medical condition that in the opinion of the Investigator precludes participation
7. Previous randomisation in the LATA trial

[a]If a historic viral load is from a diluted sample (maximum dilution 1:5), and below lower limit of quantification (LLQ), a calculated VL<100 copies/mL is allowed; if the viral load in the diluted sample is equal to the LLQ, the calculated VL should be below 50 copies/mL. If there are any viral loads measured on dried blood spots since the most recent viral load on plasma more than 12 months ago these must be below the LLQ for the assay used. The screening sample viral load must always be <50 c/mL and cannot be done using a dry blood spot. [Protocol v3.0; amended between protocols for clarity only]

[b] Highly effective contraception are injectable, implantable, oral and intrauterine contraceptives which have an expected failure rate <1% per year

5. OUTCOME MEASURES

5.1 PRIMARY OUTCOME

The proportion of participants with confirmed viral rebound, defined as 2 consecutive plasma HIV-RNA ≥50 copies/mL at any time up to the 96-week assessment.

5.2 SECONDARY OUTCOMES

5.2.1 Efficacy

(i) Proportion of participants with HIV-RNA ≥50 copies/mL at 48 and 96 weeks using a modified FDA snapshot algorithm
(ii) The proportion of participants with HIV-RNA ≥1000 copies/mL (confirmed) by week 96
(iii) The number and type of HIV mutations at confirmed virological rebound
(iv) HIV-RNA <50 copies/mL and no switch to second-line ART for treatment failure at 24, 48, 72[2] and 96 weeks

5.2.2 Safety

(i) Change in toxicity profile including change in metabolic parameters (lipids, HbA1c, phosphate), renal function (eGFR) from baseline to 96 weeks; change in anthropometric measures from baseline to 48 and 96 weeks
(ii) Time to any new or recurrent WHO stage 3 or WHO stage 4 event or death
(iii) Incidence of serious, grade 3, 4 and 5, and treatment-modifying (of any grade) adverse events
(iv) The proportion of participants with any change from baseline ART regimen
(v) Change in CD4+ and CD8+ T-cell count from baseline to 48 and 96 weeks

5.2.3 Patient-reported outcomes

(i) Adherence, acceptability, wellbeing and including neuropsychiatric problems (e.g. depression, anxiety and sleep disturbance)
(ii) Healthcare resource utilisation (a sub-study outcome)
(iii) Health-related quality-of-life (a sub-study outcome)

6. SAMPLE SIZE CALCULATIONS

Non-inferiority of SCT will be assessed by the difference between the SCT group and the CT group in the estimated proportion of participants with viral rebound (defined as the first of two consecutive HIV-1 RNA ≥50 c/mL) by week 96.

The BREATHER Plus trial was designed with a fixed non-inferiority margin of 10%. At the design stage, it was estimated that a total of 460 participants (230 per arm) would provide 90% power to exclude a non-inferiority margin of 10% for the difference in the proportion of participants reaching the primary endpoint assuming 11% have had confirmed viral rebound (2 consecutive HIV RNA ≥ 50 c/mL) by 96 weeks in both arms, 10% loss to follow-up and a two-sided α of 0.05.

[2]Version 2.0 of the protocol stated that the visit schedule would be 8-weekly visits throughout the trial (week 0, 4 [SCT only], 8, 16, 24, 32, 40, 48, 56, 64, 72, 80, 88, 96 and then every 8 weeks), with a secondary outcome of HIV-RNA <50 c/mL and no switch to second-line ART for treatment failure defined at week 64. However, under protocol version 3.0, visits will be 12-weekly in year 2 (week 0, 4 [SCT only], 8, 16, 24, 32, 40, 48, 60, 72, 84, 96 and then every 12 weeks), where visits will no longer be conducted at week 64.

6.1 THE SAFE NON-INFERIORITY FRONTIER

There is uncertainty around the expected control event risk and the conservativeness of the assumed values. In order to protect against unexpected control event risks, methods based on non-inferiority frontiers will be implemented [1]. A non-inferiority frontier is a curve that defines the appropriate non-inferiority margin for each value of the control event risk. Most trials assume a fixed risk difference frontier, i.e. they are designed so that the non-inferiority margin remains fixed whatever the control event risk is. However, this has implications: if the control event risk turns out to be much lower than assumed, then a 10% non-inferiority margin might be considered too large, making results difficult to interpret; alternatively, if the control event risk turns out to be much larger than assumed, power would be lost if the same non-inferiority margin were used.

Given that the assumed confirmed viral rebound risk in CT arm ("control event risk") for the sample size calculation was conservative (i.e. the event risk was assumed on the high side, given the literature), it is important to protect against lower-than-expected risks (i.e. substantially lower than 11%), in order to preserve interpretability of results. A control event risk much larger than 11% is not considered likely in this trial. We will use the Smooth Away From Expected (SAFE) non-inferiority frontier for the main analysis, as shown in Figure 6.1. Accordingly, the non-inferiority margin to be used will depend on the observed confirmed viral rebound risk in the CT arm; should the confirmed viral rebound risk in the CT arm be substantially lower than 11% by 96 weeks, i.e. less than 9%, we will modify the non-inferiority margin (currently set at 10%). For example, if the confirmed viral rebound risk in CT arm was 5%, the non-inferiority margin would be modified to 8%; in the unlikely event that it was as low as 1%, the non-inferiority margin would be modified to 5%.

Simply using observed data to change the non-inferiority margin would lead to inflation of type 1 error. For this reason, if the non-inferiority margin is being changed, the significance level will be modified to control the type 1 error to ≤3%. Provided that the observed confirmed viral rebound risk in the CT arm is not lower than 9%, a 95% two-sided confidence interval will be computed for the difference in confirmed viral rebound between SCT and CT arms and a 10% non-inferiority margin will be used. If the observed confirmed viral rebound risk in the CT arm is less than 9%, a 99% two-sided confidence interval will be computed for the difference in confirmed viral rebound between SCT and SCT arms; the non-inferiority margin will depend on the observed confirmed viral rebound risk as shown in Figure 6.1 and Table 6.1. If the upper bound of the respective CI is no higher than the selected non-inferiority margin, then the null hypothesis will be rejected and SCT will be declared non-inferior to CT.

Different non-inferiority frontiers imply different null hypotheses, and hence different powers to reject the null under the alternative hypothesis. Table 6.1 shows the power of the trial if the SAFE frontier is used, depending on the true control event risk. The power for the expected event risk of 11% remains high at 87.5% and is always above 80% for lower control event risks. Power for larger event risks decreases, similarly to how it would change in a standard non-inferiority trial with fixed margin. Table 6.1 reports type 1 error rates as well, which remain close to 2.5% whatever the (unknown) true control event risk is. The probability of wrongly changing the margin under the null hypothesis is 15%.

Figure 6.1 Choice of non-inferiority margin based on observed confirmed viral rebound risk using the Smooth Away From Expected (SAFE) frontier

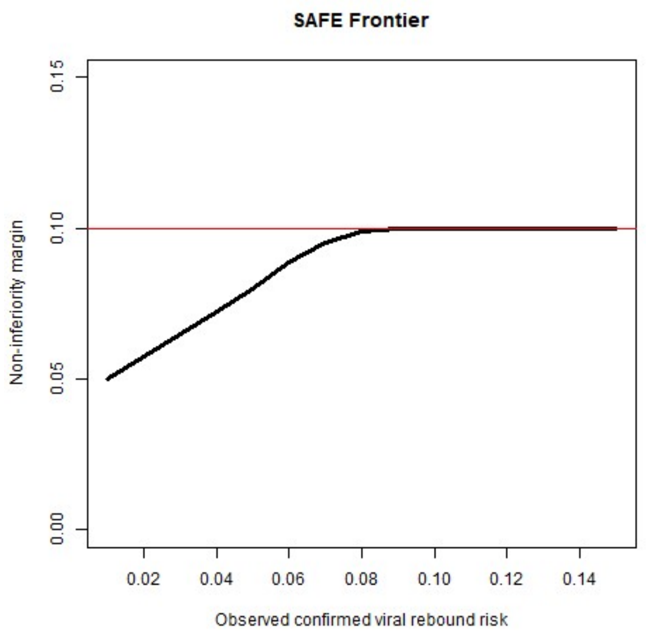

Table 6.1 Choice of non-inferiority margin and significance level based on observed confirmed viral rebound risk using the Smooth Away From Expected (SAFE) frontier

Observed confirmed viral rebound risk (P0)*	1%	2%	3%	4%	5%	6%	7%	8%	9%	10%	11%	12%	13%	14%	15%
**NI Margin**	5.0%	5.8%	6.5%	7.3%	8.0%	8.9%	9.5%	9.9%	10.0%	10.0%	**10.0%**	10.0%	10.0%	10.0%	10.0%
**Significance level**	0.5%	0.5%	0.5%	0.5%	0.5%	0.5%	0.5%	0.5%	2.5%	2.5%	**2.5%**	2.50%	2.50%	2.50%	2.50%
**Power**	95.8%	89.1%	84.4%	81.4%	80.4%	81.1%	82.2%	83.3%	84.6%	85.0%	**86.3%**	86.6%	85.7%	84.0%	82.0%
**Type 1 error**	2.50%	2.28%	2.36%	2.53%	2.67%	2.76%	2.86%	2.74%	2.69%	2.71%	**2.69%**	2.66%	2.64%	2.63%	2.61%
**P (change margin)****	100%	100%	100%	99.9%	99.0%	95.0%	84.8%	67.8%	47.5%	29.0%	**15.5%**	12.1%	6.1%	2.8%	1.2%

The column in bold corresponds to the sample size calculation assumption made at the design stage.

*The choice of non-inferiority margin and significance level will depend on the observed confirmed viral rebound risk. The power, type 1 error and probability of changing margin depend on the true control event risk.

**The probability of changing the margin is the probability that, for a given true control event risk, the observed control event risk will be lower than 9%, hence leading to using a non-inferiority margin in the analysis different from the originally planned 10%.

7. ANALYSIS POPULATIONS FOR ANALYSIS

7.1 INTENTION-TO-TREAT POPULATION AND ANALYSIS

The intention-to-treat (ITT) population will consist of all randomised participants excluding those demonstrably randomised in error; where randomisation in error will be judged by the participant meeting a major violation of the eligibility criteria, including for example a participant being HIV negative, and will not depend on treatment allocation or post-randomisation follow-up. Major violation of the eligibility criteria will be determined by clinical decision, blind to treatment allocation.

The BREATHER Plus trial will be analysed as ITT. This will be the primary population used for the main analysis, which will use the randomised treatment allocation rather than actual treatment received. Primary inference will be based on the primary endpoint analysis of the ITT population. ITT was chosen over the per protocol analysis (described below) as it respects randomisation and ensures that the two treatment arms are, on average, comparable in terms of prognostic factors.*

7.2 PER PROTOCOL POPULATION AND ANALYSIS

The per protocol population will consist of all randomised participants excluding those who did not meet all the eligibility criteria and excluding any participants randomised in an incorrect stratum. Participants will also be excluded if they report taking <75% intended weekend breaks (SCT arm only; according to self-reported adherence) or <90% of time on ART (CT arm only; according to ART log) to earliest of 96 weeks or censoring date (see below).

Follow-up will be censored if a participant:

- had a break in any component of ART regimen for more than 7 days (SCT and CT arms);
- changed ART component for any reason, excluding changes between TDF and TAF and 3TC and FTC, and vice versa (SCT and CT arms);
- changed to continuous therapy for reasons other than confirmed viral rebound (SCT arm only)**.

A per protocol analysis of the primary endpoint will be done in the per protocol population.

* Some researchers consider that, in non-inferiority trials, a per protocol analysis is more appropriate than the ITT analysis because of the belief that under the null hypothesis of inferiority, the ITT analysis is anti-conservative in the presence of deviations from the protocol and missing data. However, simulation studies have demonstrated that this is not always the case and that the anticonservatism or otherwise depend on a number of factors including the nature of protocol deviations and the reasons for missing data (see for example [2, 3]). A per protocol analysis violates the integrity of the randomisation process. Consequently, estimates of the treatment difference obtained via per protocol analysis reflect a combination of a true treatment effect and subset selection bias and it is impossible to disentangle the two. These considerations which are widely accepted in superiority trials apply equally well to non-inferiority designs (see for example [4]).

**Note this would include changing from SCT to CT for pregnancy or while receiving TB treatment. Switch from SCT to CT for viral rebound following a single HIV-1 RNA ≥50 c/mL (where confirmatory measure was not performed before change in strategy) is considered "confirmed viral rebound" hence is not a reason for censoring (see section 10.8).

8. STRATA AND COVARIATES

8.1 STRATIFICATION VARIABLES

Randomisation will be stratified by:

- Centre
- Mode of infection (horizontal or vertical)[3]

---

[3] Vertical transmission defined as acquiring HIV through mother to child transmission (MTCT); horizontal transmission defined as acquiring HIV through other routes (sexual contact, blood product, unknown or other).

9. DATA HANDLING AND DERIVATION

9.1 DEFINITION OF BASELINE

Baseline is defined as the date of randomisation (day 0). For all variables, the week 0 measurement is defined as the latest single measurement up to 42 days prior to (and including) date of randomisation.

9.2 DEFINITION OF FOLLOW-UP TIME

Time will be measured from randomisation (day 0). For analysis of the primary endpoint, follow-up will be to end of week 96 window [week 102 date – 1 day]. For safety and occurrence of new or recurrent WHO 3 or WHO 4 event or death analysis, the primary analysis will include all follow-up to end of trial visit. If participants are censored earlier due to loss to follow-up or withdrawal of consent, it will be assumed that such censoring is independent of the outcome. For interim analyses, the date of data freeze will be used. For analyses by scheduled visits, only time-points where there are at least 10 participants in each group with a measurement will be included.

9.3 DEFINITION OF NOMINAL WEEK FOR CLINICAL AND LABORATORY MEASUREMENTS

Laboratory measurements, and other clinical parameters (e.g. weight), at any nominal week are defined as those taken nearest to the nominal week within equally spaced windows according to the protocol visit schedule (note that the visit schedule changes frequency). The midpoint between two scheduled visit weeks should be taken as belonging to the latter window (see below). Where there are two values within one of these equally spaced windows, but both equidistant from the nominal visit week, the later value will be used.

**Week 4 (SCT only*):**	2 ≤ week < 6 [between 2 & 6 weeks, including 2 but excluding 6]
**Week 8*:**	4 ≤ week < 12
**Week 16:**	12 ≤ week < 20
**Week 24:**	20 ≤ week < 28
**Week 32:**	28 ≤ week < 36
**Week 40:**	36 ≤ week < 44
**Week 48:**	44 ≤ week < 54
**Week 60:**	54 ≤ week < 66
**Week 72:**	66 ≤ week < 78
**Week 84:**	78 ≤ week < 90
**Week 96:**	90 ≤ week < 102

For all the subsequent visit weeks beyond week 96 (i.e. every 12 week follow-up visits), the same rule of +/- 6 weeks defining intervals will be applied as described above.

For laboratory measurements or assessments taken less frequently in line with the protocol schedule, the nominal week windows will be widened according to the above principle, as determined by scheduled visits and midpoint between scheduled visits. For measurements taken every 48 weeks, a rule of +/- 24 weeks defining intervals will be applied; measurements taken every 24 weeks, a rule of +/- 12 weeks will be applied; while measurements taken every 16 weeks, a rule of +/- 8 weeks will be applied.

*Week 4 visits will be conducted in the pilot and SCT arm only and will provide descriptive data at this timepoint. Week 8 is designed as the first comparative timepoint between trial arms, therefore week 4 measurements taken in SCT arm may contribute to cross-sectional week 8 analyses if they fall in the week 8 window and there is no nearer measurement. If anyone in either arm in the pilot, meets the primary endpoint at ≤4 weeks, they will be included as a failure at week 8.

9.4 HANDLING MISSING DATA

Complete case analysis will be performed for all analyses except for analyses using HIV-1 RNA and immunology data as described below.

9.4.1 HIV-1 RNA data

For analysis of the primary endpoint (section 10.8) and other virological outcomes (section 10.12.1), except for the FDA snapshot analysis, multiple imputation will be applied if either of the following criterium is met:

- 5% of all HIV-1 RNA measurements at scheduled visits are missing

Or

- 10% of confirmatory HIV-1 RNA measurements are missing.

If the criterium for imputation is met, missing HIV-1 RNA measurements at scheduled visits will be multiply imputed based on the procedure outlined in Appendix 1

9.4.2 Immunology data

Missing immunology data imputation will be performed as follows (following confirmation with participating labs):

- Immunology (CD4%, CD4+ count and total lymphocyte): If one of the three measurements is missing, the standard formula [CD4+ count = CD4% x Lymphocyte count/100] will be used to calculate the missing result using the remaining two available values. The corresponding formula is also used for CD8+.

9.5 HANDLING OF VIRAL LOAD DATA

The primary endpoint requires confirmation of HIV-1 RNA measurements ≥50 c/mL at any time up to end of week 96 window (week 102 date – 1 day), with the first of 2 consecutive HIV-1 RNA≥50 c/mL strictly measured prior to the end of week 96 window.

For real-time HIV-1 RNA (results obtained from "immediate" testing of samples taken at scheduled visits, which are entered directly onto the database by sites, as opposed to retrospective testing of stored samples), participants with HIV-1 RNA≥50 c/mL will be recalled within the respective visit week window to confirm their HIV-1 RNA result. EDTA-plasma samples will be stored at weeks 0, 4 (SCT only), 8, 16, 24, 32, 40, 48 and then 12-weekly from protocol version 3.0 for batched retrospective viral load testing where real time HIV-1 RNA results are not available (see Appendix 2 for proposed rules for requesting retrospective HIV-1 RNA testing from stored plasma samples and Section 10.8 for details of use of HIV-1 RNA data results for primary endpoint analysis).

The date of the first of two consecutive HIV-1 RNA measurements ≥50 c/mL will be used for calculating the time to the first confirmed measurement ≥50 c/mL (primary endpoint rebound date). For the cross-sectional analysis at weeks 24, 48, 72 and 96, where multiple measurements fall within the same visit window, the closest measurement to scheduled visit will be used as described in Section 9.3.

9.6 HANDLING ADVERSE EVENT DATA

Serious Adverse Events (SAEs) and notable events are reported to the CTU within 1 working day of the site becoming aware of the event. All SAEs, notable events, new or recurrent WHO 3 or 4 events, ART modifying events of any grade, clinical and clinically significant laboratory events of grade ≥3 during the trial will be reviewed by MRC CTU clinical reviewers. Asymptomatic laboratory events (that are not clinically significant) of grade ≥3 close in calendar time (±28 days) to clinical events will also be reviewed by the clinical reviewers to avoid double-reporting of events.

Clinical and laboratory adverse events will be graded according to the toxicity grading tables found in Appendix I of the protocol. Only new events or new recurrences of events that occur post-randomisation will be included in the analysis. This includes any event (clinical or laboratory) of grade ≥3 and ART modifying events (clinical or laboratory) of any grade. Ongoing events present at randomisation which had been reported prior to entry into the trial will only be reported as baseline data. For adverse event analysis, "Date of event diagnosis" on AE eCRF will be used as date of event. All deaths that occur after the date of randomisation up to end of trial will be included as BREATHER Plus events. The "date of death" will be used as the date of event for deaths. When calculating incidence rates, the time to the first event and recurrence will be used in the main comparison between the two study arms. For AEs where the site clinician and CTU clinical reviewer disagree on seriousness, the event will be reported as an SAE.

9.7 OTHER DEFINITIONS

- The WHO staging of HIV infection is defined according to diagnostic criteria for WHO stage 1, 2, 3 and 4 conditions [5] are described in the protocol (Appendix II).
- Clinical and laboratory adverse events will be classified by System Organ Class (SOC) according to version 25.0 of Medical Dictionary for Regulatory Activities (MedDRA).
- Gradings of clinical and laboratory adverse events (AEs) are defined according to the Adapted Division of AIDS (DAIDS) Table for Grading the Severity of Adult and Pediatric Adverse Events (Corrected Version 2.1 - July 2017), in the protocol (Appendix IV - Toxicity Gradings) [6]. Neutrophil grading is based on WHO guidelines [7] recognising the lower normal levels in African populations. For creatinine clearance DAIDS ranges of estimated creatinine clearance rather than comparison with the baseline will be used.
- New asymptomatic grade ≥3 laboratory events will be identified by detection of follow-up measurements of grade ≥3 starting from a baseline measurement of grade <3. The laboratory event will be classified as grade 3 or 4 according to the maximum grade achieved before resolution. Resolution is defined as return to a minimum of grade 2 or baseline grade.
- Pregnancies occurring during the trial are reported as notable events (protocol Section 7.2).

9.8 DATA QUALITY

General data quality is monitored within the OpenClinica database through real time generation of database queries, as described in the BREATHER Plus Data Management Plan. Additional data checks will be carried out at least monthly or as required by the statistician, depending on accrual, in order to ensure ongoing data quality. In addition to this, extensive data cleaning will take place before each IDMC meeting and final analysis.

10. STATISTICAL ANALYSES

10.1 GENERAL PRINCIPLES

The two arms of the trial will be compared as randomised according to the intention-to-treat principle. For the primary endpoint, a per protocol analysis will also be performed. Descriptive statistics will be reported overall and by randomised group, and percentages will be of non-missing values, with the number (%) of non-missing values given if data are not complete. Statistical tests will be 2-sided, estimates will be presented with a 2-sided 95% CI (or 99% as required per the primary analysis) and comparisons will adjust for the stratification factors (all combinations of centre and mode of infection, where sufficient participants are enrolled across strata). Appropriate transformations for all variables will be applied after inspection of the data.

Time-to-event outcomes will consider time from randomisation to the event date, using Kaplan-Meier estimation. Differences between treatment groups in time-to-event outcomes will be tested using adjusted Cox proportional hazard regression models (adjusted for stratification factors). All hazard ratios or estimates of the difference between groups will be presented with a 2-sided 95% CI and p-value.

Differences between randomised groups in binary outcome variables will be tested using Chi-squared tests or Fisher's exact tests, as appropriate. Fisher's exact test will be used if expected frequencies in one or more of the cells in 2x2 matrices is <5, otherwise Chi-square test will be used. Logistic regression models will be used for adjusted analyses (presented with 2-sided 95% CI).

Percentages will be reported to 0 decimal places, unless <0.5% when they will be given to one decimal place. P-values will be given to 3 significant figures. There will be no formal adjustment of α-values because of any interim analyses performed for IDMC meetings.

All analyses will be performed using the STATA software (updated and validated), unless otherwise specified.

10.2 ANALYSIS OF CONTINUOUS VARIABLES

For the analysis of continuous variables, the mean change from baseline over follow-up will be calculated using linear mixed models (unstructured covariance) with random intercept for participants and fixed effects for randomised group and visit weeks, including interaction between randomised group and visit weeks, adjusting for baseline. Adjusted analyses will be performed adjusting for stratification factors in addition to baseline.

For each of the scheduled follow-up visit weeks, the number (%) of participants with a measurement along with the following will be reported based on the above linear mixed models:

- Unadjusted (only adjusting for baseline) difference between arms: mean (SE), 95% CI, p-value.
- Adjusted (adjusting for baseline and stratification factors) difference between arms: mean (SE), 95% CI, p-value.
- Mean change from baseline, by arm (estimated from adjusted model, assuming baseline mean): mean (SE)

For each analysis, a graph showing the mean change from baseline across scheduled visits by randomised group will be presented including 95% CI.

Average treatment differences through follow-up will be estimated with 2-sided 95% confidence intervals by fitting linear mixed models with random intercept for participants and fixed effects for randomised group and visit weeks, adjusting for baseline measurement. The adjusted model will also adjust for stratification factors.

10.3 ANALYSIS OF ADVERSE EVENT DATA

Clinical and laboratory adverse events will be tabulated by MedDRA System Organ Class and Preferred Term (PT). Randomised groups will be compared in terms of time to first adverse event using Cox proportional hazard regression models calculating hazard ratios. Multiple events will be compared between randomised groups using Poisson regression models to calculate the incidence rate ratios of adverse events in the SCT vs CT arms adjusted for clustering within individuals. The 95% CI will be given along with p-value for the unadjusted and adjusted models. The adjusted model will be adjusted for the stratification factors. The total number of adverse events and number of participants with at least one adverse event will be computed. Adverse event rates (per 100 person years) will be calculated as the number of events/total person years at risk*100 (presented with 2-sided 95%CI).

10.4 ANALYSIS OF PARTICIPANT AND PARENT/CARER QUESTIONNAIRE DATA

Descriptive analysis of questionnaire data will be undertaken for carer and participant completed questionnaires separately. Participant and parent/carer reported binary responses will be compared between randomised groups over time using logistic mixed models with a random effect for intercept and fixed effects for treatment group, post-randomisation study visits and adjustment covariates [baseline answer and stratification factors]. The ordered categorical responses will be compared between randomised groups over time using ordered logistic mixed model with a random effect for intercept and fixed effects for treatment group, post-randomisation study visits and adjustment covariates [baseline answers and strata].

10.5 ANALYSIS DETAILS

10.5.1 Enrolment and eligibility

- **Enrolment over time:** plot by calendar month
- **Enrolment by arm:** number (%)
- **Enrolment by country and site:** number (%)
- **Enrolment by stratification factors:** number (%)
- **Numbers screened, randomised, not randomised including tabulation of reason for non-randomisation**
- **Details of minor and major ineligibility and number excluded from analysis:** number (%)
- **Trial CONSORT diagram:** details of the number of participants screened for eligibility, the number not randomised and reasons why, the number randomised to each arm, and the number in follow-up at week 96, and the number included in the primary analysis.

10.5.2 Baseline characteristics

Baseline characteristics will be summarised using descriptive statistics for all randomised participants. No statistical significance tests will be used to compare treatment arms.

10.5.2.1 Demographics

- **Sex at birth:** number (%) by sex (male, female)
- **Age (years):** number of participants and missing; mean, standard deviation (SD), median, interquartile range (IQR), range; number (%) in categories (12-<15, 15-<18, 18-<20 years).
- **Ethnic origin:** number (%) in categories (Asian, Black, Hispanic/Latino, Caucasian, other); number missing.

10.5.2.2 HIV and immune related parameters

- **Mode of infection:** number (%) in categories (mother to child, blood product, sexual transmission, other, unknown); number missing.
- **WHO staging for HIV:** number (%) in categories (1, 2, 3, 4); number missing.
- **CD4%:** number of participants and missing; mean, SD, median, IQR, range; number (%) in categories (<10%, 10-<15%, 15<20%, 20-<25%, 25-<30%, 30-<40%, ≥40%).
- **CD4+ absolute (cells/mm^3):** number of participants and missing; mean, SD, median, IQR, range; number (%) in categories (<350, 350-1000, 1000-1500, ≥1500).
- **CD8%:** number of participants and missing; mean, SD, median, IQR, range.
- **CD8+ absolute (cells/mm^3):** number of participants and missing; mean, SD, median, IQR, range.
- **Total Lymphocyte count (cells/mm^3):** number of participants and missing; mean, SD, median, IQR, range.
- **HIV-1 RNA:** number (%) in categories (<50 or ≥50 c/mL); number missing.

10.5.2.3 Growth parameters

- **Weight (kg):** number of participants and missing; mean, SD, median, IQR, range; number (%) in categories (<35kg, 35-<40kg and ≥40kg).
- **Height (cm), BMI (kg/m^2):** number of participants and missing; mean, SD, median, IQR, range.
- **Weight-for-age, height-for-age, BMI-for-age, z-score#:** number of participants and missing; mean, SD, median, IQR, range; number (%) in categories (<-3, -3 to <-2, -2 to <0, ≥0).
- **Waist (cm), hip (cm):** number of participants and missing; mean, SD, median, IQR, range.
- **Waist-hip ratio:** number of participants and missing; mean, SD, median, IQR, range.

#BMI, weight and height will also be analysed as BMI-for-age, weight-for-age and height-for-age z-scores. For BMI, weight and height, British 1990 Reference data (0–23 years), which covers the full age range of BREATHER Plus participants, will be used for standardisation.

10.5.2.4 Laboratory data

Lipid Results

- **Triglycerides (mmol/L):** number of participants and missing; mean, SD, median, IQR, range.
- **LDL Cholesterol (mmol/L):** number of participants and missing; mean, SD, median, IQR, range.
- **HDL Cholesterol (mmol/L):** number of participants and missing; mean, SD, median, IQR, range.
- **Total Cholesterol (mmol/L):** number of participants and missing; mean, SD, median, IQR, range.
- **Phosphate (mmol/L):** number of participants and missing; mean, SD, median, IQR, range.

Blood Biochemistry Results

- **Albumin (g/dL):** number of participants and missing; mean, SD, median, IQR, range.
- **Alanine transaminase (IU/L):** number of participants and missing; mean, SD, median, IQR, range.
- **Total Bilirubin (mg/dL):** number of participants and missing; mean, SD, median, IQR, range.
- **Creatinine (mg/dL):** number of participants and missing; mean, SD, median, IQR, range.
- **Creatinine clearance (eGFR)* (mL/min):** number of participants and missing; mean, SD, median, IQR, range.

*Calculation of eGFR will be performed using the Cockcroft-Gault formula

Haematology Results

- **Haemoglobin (g/dL):** number of participants and missing; mean, SD, median, IQR, range.
- **White blood cell count (cells/mm^3):** number of participants and missing; mean, SD, median, IQR, range.
- **Absolute lymphocyte count (cells/mm^3):** number of participants and missing; mean, SD, median, IQR, range.
- **Platelet count (cells/mm^3):** number of participants and missing; mean, SD, median, IQR, range.
- **Absolute neutrophil count (cells/mm^3):** number of participants and missing; mean, SD, median, IQR, range.
- **Glycosylated haemoglobin A1c (mmols/mol):** number of participants and missing; mean, SD, median, IQR, range.

10.5.2.5 Antiretroviral exposure pre-randomisation

- **Different ART drugs ever received, overall and by class:** median, range, summarised by categories (All classes, nucleoside/nucleotide reverse transcriptase inhibitor [NRTI], protease inhibitor [PI], non-nucleoside reverse transcriptase [NNRTI], integrase inhibitors [INSTI]).
- **Participants exposed to ART classes:** number (%) in categories (NRTI+PI only, NRTI+NNRTI only, NRTI+INSTI only, NRTI+PI+INSTI only, NRTI+NNRTI+INSTI only, NRTI+NNRTI+PI only, NRTI+NNRTI+PI+INSTI, DTG as a separate category).
- **Cumulative ART exposure in years, overall and by class:** median, range, summarised by categories (All classes, NRTI, PI, NNRTI, INSTI, DTG as a separate category)

10.5.2.6 Antiretroviral regimen at randomisation

- **ART regimen at randomisation by NRTI backbone:** number (%) in categories (TDF+3TC, TDF+FTC, TAF+3TC, TAF+FTC)

10.5.2.7 Questionnaire data

- Acceptability, neuropsychiatric symptoms, suicidal ideation and health-related quality of life questionnaire data will be summarised using frequency table and summary statistics.

10.6 FOLLOW UP AND AVAILABILITY OF HIV-1 RNA MEASUREMENTS

10.6.1 Follow up

Follow-up will be presented up to date of last follow-up or the date of data freeze for interim analysis, overall and by randomised group.

- **Number of participants randomised and seen after baseline.**
- **Weeks from randomisation to most recent study visit[¥]*:** number (%) in week categories (0, 1 to <4 weeks, 4 to <8 weeks, 8 to <16 weeks, 16 to <24 weeks, 24 to <32 weeks, 32 to <40 weeks, 40 to <48 weeks, 48 to <60 weeks, 60 to <72 weeks, 72 to <84 weeks, 84 to <96 weeks)
- **Median weeks from randomisation[†]:** median (IQR), range.
- **Weeks since last seen[¥]:** number (%) in week categories (Seen in last 0-8 weeks, seen in last 8-16 weeks, seen in last 16-24 weeks, seen in last 24-32 weeks, seen in last 32-40 weeks, seen in last 40-48 weeks). This will be reported at interim analyses.
- **Died or confirmed by site to have withdrawn consent:** number (%)
- **Potentially LTFU****: number (%)
- **LTFU/withdrawal:** number (%), timing and reason
- **Reached primary endpoint or seen at week 96: number (%)**
- **Scheduled visit attendance:** number (%) of scheduled study visits attended, overall and by assessment week.

* Week number is based on exact time in weeks rounded to the nearest week number given on CRF. This is not based on nominal windows around visit week.
[†] Using exact time rounded to the nearest week.
** Defined as either withdrawn consent or not seen for >24 weeks
[¥] More week categories may be added as required.

10.6.2 Availability of HIV-1 RNA measurements

- **Availability at week X*:** number (%), available/missing

* Week number is determined by the window in which the visit date falls (see section 9.3). This means a participant may have a week X eCRF but if this does not fall in the window for week X and no other results fall in the window then they will be listed as missing.

10.7 TREATMENT ADHERENCE AND PROTOCOL DEVIATIONS

10.7.1 Use of antiretroviral therapy after randomisation

- **Follow-up regimen:** number (%) by anchor drug and NRTI backbone of regimen.
- **Tabulation of reason for switch to continuous therapy (SCT only):** number (%) switched to continuous therapy, time of switch and reasons for switch.
- **Tabulation of reasons for any ART changes:** number (%) on initial regimen and not on initial regimen. Number (%) changed from initial regimen and reasons for change or interruption. Initial regimen is defined as the regimen participants were on at time of randomisation: dolutegravir, TDF or TAF, and 3TC or FTC.
- **List of switches to continuous therapy (SCT only):** arm, trial number, date of switch, week, initial regimen, reason for switch
- **List of ART changes:** arm, trial number, date of change, week, initial regimen, regimen after change, reason for change in ART regimen.
- **List of reasons for treatment interruption:** arm, trial number, date of change, week, duration of treatment interruption in days, regimen interrupted and reason for treatment interruption.

- **Time to discontinuation of randomised treatment (weeks):** number of participants (%); mean, SD, median, IQR, range.

10.7.2 Protocol deviations

Protocol deviations are defined in the BREATHER Plus protocol deviations list and managed according to the BREATHER Plus Protocol Deviation Management Plan, stored in the BREATHER Plus eTMF. These two documents have been developed in line with the MRC CTU protocol deviations and violations standard operating procedures (SOP).

Protocol deviations: Number (%) by protocol deviation type (critical and major).

10.8 PRIMARY OUTCOME ANALYSIS BY WEEK 96

Participants will have an end of trial visit within ±6 weeks of the last recruited participant reaching 96 weeks follow-up (with return for confirmatory viral load if viral load is ≥50 c/mL at the end of trial visit). At analysis, a participant who does not attend their end of trial visit will be classified as "lost to follow-up" if they are not known to have died and the clinic has confirmed that they are unable to contact them.

The primary outcome measure is HIV-1 RNA ≥50 c/mL (confirmed on a separate sample) at any time up to end of week 96 window (week 102 – 1 day). Real-time HIV-1 RNA will be measured at weeks 48 and 96 (and 48-weekly thereafter). Participants with HIV-1 RNA ≥50 c/mL will be recalled for blood draw within the week 48 analysis window (44-54 weeks), week 96 analysis window (90-102 weeks), then 48 weekly (±6 weeks) and at the end of trial visit (+6 weeks) to confirm their HIV-1 RNA results. If a real time HIV-1 RNA is ≥50 c/mL at any other scheduled visits, participants will also be recalled for blood draw within the respective week window. A participant who has one HIV-1 RNA ≥50c/mL and switches ART for treatment failure or from SCT to CT for viral load rebound will be assumed to have met the primary outcome at the HIV-1 RNA ≥50c/mL prior to switch.

At visits where real time HIV-1 RNA are not measured, stored samples will be used for HIV-1 RNA measurement retrospectively (see Appendix 2 for proposed rules for requesting retrospective HIV-1 RNA testing from stored plasma samples; Appendix 3 for HIV-1 RNA data considerations/assumptions for analysis relating to presence of real-time and retrospective HIV-1 RNA results within same window and HIV-1 RNA results measured on diluted samples).

Date of first of 2 consecutive HIV-1 RNA ≥50 c/mL will be used as date of meeting the primary endpoint (virologic rebound). The study has been powered to examine the difference in proportion of children with confirmed viral rebound in SCT arm compared to the CT arm and therefore the conclusion regarding non-inferiority will be driven by this.

If the criterium for imputing missing results has not been met (section 9.4.1) and HIV-1 RNA measurement(s) are missing at a scheduled visit week(s) or at recall(s) for repeat:

- The participant will be assumed to be virologically suppressed between visits if the HIV-1 RNA result prior to missed visit/repeat is <50 c/mL.
- The participant will be assumed to be virologically suppressed from baseline until their first non-missing HIV-1 RNA result.
- The participant will be assumed to be virologically suppressed between visits if HIV-1 RNA result prior to missed visit/repeat is ≥50 c/mL and next available result is <50 c/mL.
- The participant will be considered to have met the primary endpoint for confirmed viral rebound if HIV-1 RNA result prior to missing visit/repeat is ≥50 c/mL and participant switches ART for treatment failure or from SCT to CT for or viral rebound before confirmatory HIV-1 RNA test. Date of confirmed viral rebound will be the date of HIV-1 RNA ≥50 c/mL prior to missed visit/repeat.
- The participant will be considered to have met the primary endpoint for confirmed viral rebound if HIV-1 RNA prior to missed visit/repeat is ≥50 c/mL and next available result is ≥50 c/mL. Date of confirmed viral rebound will be the date of HIV-1 RNA ≥50 c/mL prior to missed visit/repeat.

- The participant will be censored if the last available HIV-1 RNA is ≥50 c/mL, after which the participant is LTFU, withdrew or died prior to end of week 96 window. Participant will be censored at the last HIV-1 RNA<50 c/mL before last available HIV-1 RNA ≥50 c/mL (or at baseline if the only available viral load during trial follow-up was ≥50 c/mL).

For those participants who have not already reached the primary endpoint by their last visit prior to end of week 96 window [week 102 date – 1 day], we will determine their primary endpoint status as follows, provided that criterium for imputing missing results has not been met (section 9.4.1):

**Last HIV-1 RNA *before* W102 date*** (end of week 96 window)	**Last seen in clinic *on or after* W102 date** (end of week 96 window)	**Primary endpoint status**	**Primary endpoint date**
**<50 c/mL**	Yes	Censored	W102 date – 1 day
	No	Censored	Date of last HIV-1 RNA
**≥50 c/mL**§** (no repeat)	Yes & first HIV-1 RNA on/after W102 date <50 c/mL	Censored	W102 date – 1 day
	Yes & no HIV-1 RNA on/after W102 date	Censored	Date of last HIV-1 RNA<50c/mL before the last HIV-1 RNA≥50c/mL before W102 date
	Yes & first HIV-1 RNA on/after W102 date ≥50 c/mL	Reached endpoint	Date of last HIV-1 RNA ≥50 c/mL before W102 date
	No	Censored	Date of last HIV-1 RNA<50 c/mL before the last HIV-1 RNA≥50 c/mL before W102 date

* The last HIV-1 RNA result can be at any scheduled visit after randomisation before end of week 96 window. Anyone with no viral load data after randomisation (or week 4 in the SCT arm, where HIV1-RNA<50c/mL at week 4) will be censored at baseline. For example, if the participant was LTFU or withdrew or died at week 48, their last available HIV-1 RNA may be at week 48. The table illustrates how we will determine the primary endpoint status by week 96 depending on the last HIV-1 RNA result (< or ≥50 c/mL) available before end of week 96 window and if participants are last seen on or after end of week 96 window with or without HIV-1 RNA result.

** Last HIV-1 RNA≥50 c/mL available before end of week 96 window missing a repeat could be a real time measurement ≥50 c/mL missing a repeat; or, a measurement obtained from retrospective testing of stored sample, where repeat request is not applicable.

§ Treatment switch for failure or change from SCT to CT for viral rebound on or after an unconfirmed HIV-1 RNA≥50c/ml will be considered as confirmation of viral rebound; date of primary endpoint date will be date of last HIV-1 RNA≥50c/mL before W102 date.

If the criterium for imputation is met (9.4.1), missing HIV-1 RNA measurements will be multiply imputed as described in Appendix 1. For analysis of the primary endpoint by week 96, missing HIV-1 RNA measurements at any of the scheduled follow-up visits up to and including week 108 visit will be multiply imputed.

For those participants who have not already reached the primary endpoint by their last visit prior to end of week 96 window [week 102 date – 1 day], we will determine their primary endpoint status as follows, provided that the criterium for imputation has been met:

**Last HIV-1 RNA (in W96 window¥) *before* W102 date (end of week 96-window)**	**Last seen in clinic *on or after* W102 date** (end of week 96 window)	**Primary endpoint status**	**Primary endpoint date**
**<50 c/mL**	Yes	Censored	W102 date – 1 day
	No	Censored	Date of last HIV-1 RNA
**≥50 c/mL** (no repeat observed)**‡ §**	Yes & W108 HIV-1 RNA<50 c/mL	Censored	W102 date – 1 day
	Yes & W108 HIV-1 RNA≥50 c/mL	Reached endpoint	Date of last HIV-1 RNA≥50 c/mL before W102 date
	**Imputed W108 HIV-1 RNA**	To be determined by imputed W108 HIV-1 RNA **(Reached endpoint if imputed HIV-1 RNA≥50 c/mL)**	To be determined by imputed W108 HIV-1 RNA **(Date of last HIV-1 RNA≥50 c/mL before W102 date if reached endpoint)**
		To be determined by imputed W108 HIV-1 RNA **(Censored if imputed HIV-1 RNA<50 c/mL)**	To be determined by imputed W108 HIV-1 RNA **(W102 date – 1 day if censored)**

¥ This table refers to last HIV-1 RNA in W96 window because it illustrates how we will determine the primary endpoint status by week 96 given that all missing results at previous scheduled visits will have been multiply imputed.
‡ Last HIV-1 RNA≥50 c/mL before W102 date with no repeat observed could be, for example, a real time week 96 HIV-1 RNA measurement where repeat sample was lost and no further samples available.
§ Treatment switch for failure or change from SCT to CT for viral rebound on or after an unconfirmed HIV-1 RNA≥50c/ml will be considered as confirmation of viral rebound; date of primary endpoint date will be date of last HIV-1 RNA≥50c/mL before W102 date.

For the primary analysis, the two treatment groups (SCT and CT) will be compared in the intention-to-treat population. The comparison will be of the cumulative probability of confirmed viral rebound by week 96 (as defined above); multiply imputed HIV-1 RNA data will be used as described in section 9.4.1. To allow for censoring, the survival curve for each combination of strata and randomised group will be calculated using a Cox model adjusting for stratification factors (as appropriate where sufficient participants are enrolled across strata) and randomised group. The average cumulative failure function (1-survival curve) for each randomised group will be estimated by standardisation procedure [8] as a weighted average of the corresponding stratum-specific cumulative failure functions with weights equal to the prevalence of that stratum in the whole ITT population. The difference in the probability of virological rebound between the SCT and CT arms will be estimated by the average difference between the cumulative failure functions at week 96. A 2-sided bias-corrected 95% CI or 99% (Selected as per in section 6) for the difference in the probability of confirmed viral rebound by week 96 (SCT – CT) will be calculated using appropriate (bias-corrected) percentiles of the bootstrap estimates. The bootstrapping will sample 10,000 times and be stratified by stratification factors. SCT will be considered non-inferior to CT if the upper limit of the respective confidence interval of the difference SCT-CT is less than the selected non-inferiority margin (Selected as per in section 6). The 95% CI or 99% of the estimated probabilities of confirmed viral rebound by week 96 in each randomised group will also bias-corrected.

Kaplan-Meier plots of cumulative probability of confirmed viral rebound stratified by treatment allocation to week 96 will be produced, alongside plots of adjusted cumulative probability of confirmed viral rebound by treatment allocation based on the Cox model.

A per protocol analysis of the primary endpoint will be carried out in the per protocol population as described in section 7.2. Analysis will be done as described previously for primary outcome analysis this section. If criterium for multiple

imputation has been met (section 9.4.1), multiply imputed HIV-1 RNA data used for primary endpoint analysis will be used.

In addition, since there may be other reasons for return from SCT to CT (e.g. persistent viral blips or clinician concern about adherence), we will consider a failure endpoint comprising HIV-1 RNA ≥50 c/mL (confirmed on a separate sample) or switch off randomised treatment regimen or strategy (except for treatment/strategy change for pregnancy) at any time up to end of week 96 window.

10.9 SENSITIVITY ANALYSES FOR THE PRIMARY OUTCOME

The following sensitivity analyses will be performed:

I. **Analysis accounting for non-proportional hazards**

The assumption of proportional hazards in the Cox regression model will be assessed through visual consideration of treatment-stratified Kaplan-Meier plots and plots of observed $\ln(-\ln(S(t))$ (requiring parallel lines for the two treatment groups). Sensitivity of conclusions to the proportional hazards assumption will be assessed by fitting a flexible parametric model[9], allowing the treatment effect to vary over follow-up using a restricted cubic spline function. In the case that proportionality of hazards is violated and overall conclusions differ with respect to non-inferiority of SCT, then the flexible parametric model will be used for the primary analysis.

II. **Analysis unadjusted for stratification factors**

Analysis unadjusted for stratification factors will be done as described previously for primary outcome analysis in section 10.8. If criterium for multiple imputation has been met (section 9.4.1), multiply imputed HIV-1 RNA data used for primary endpoint analysis (section 10.8) will be used. The bootstrapping will sample 1,000 times rather than 10,000 times used for primary outcome analysis.

III. **Analysis using non-imputed data**

If the criterium for imputing missing HIV-1 RNA results has been met, analysis of non-imputed data will additionally be done as described in section 10.8. The bootstrapping will sample 1,000 times rather than 10,000 times used for primary outcome analysis.

10.10 SUBGROUP ANALYSIS FOR THE PRIMARY OUTCOME

Subgroup analysis for primary endpoint will be performed by randomisation stratification factors (centre and mode of transmission [vertical, horizontal]), where sufficient participants are enrolled and by age category at baseline [12-<15, 15-<18, 18-<20 years].

10.11 SECONDAY ANALYSIS OF PRIMARY ENDPOINT

Secondary analyses comparing time to confirmed viral rebound between arms will be performed using Cox proportional hazard regression models, adjusted and unadjusted for the stratification factors. The adjusted and unadjusted hazard ratios will be presented with a 2-sided 95% confidence interval.

10.12 SECONDARY OUTCOME ANALYSES

10.12.1 Efficacy

10.12.1.1 Proportion of participants with HIV-1 RNA ≥50 c/mL at 48 and 96 weeks using a modified version of the FDA snapshot algorithm

The proportion of participants with HIV-1 RNA ≥50 c/mL at weeks 48 and 96 will be compared between arms using a modified version of the FDA snapshot algorithm (see Appendix 4) [10]. The estimated difference in proportion between SCT and CT arms will be computed with 95% CI by the Mantel-Haenszel weighted mean of proportions in each stratum.

10.12.1.2 The proportion of participants with HIV-RNA ≥1000 c/mL (confirmed) by week 96

The cumulative probability of confirmed viral rebound (defined as the first of two consecutive HIV-1 RNA ≥1000 c/mL) by week 96 will be estimated as described previously in section 10.8 (primary outcome analysis by week 96). If criterium for multiple imputation has been met (section 9.4.1), multiply imputed HIV-1 RNA data used for primary endpoint analysis (section 10.8) will be used. The bootstrapping will sample 1,000 times rather than 10,000 times used for primary outcome analysis by week 96.

10.12.1.3 The number and type of HIV mutations at confirmed viral rebound

In participants with confirmed HIV-1 RNA ≥50c/mL, resistance testing at failure will be performed on stored plasma samples. Activity of the background regimen will be defined according to the most current Stanford database algorithms at time of analysis. NRTI, PI, NNRTI and INSTI resistance will be defined according to the most current IAS-USA list of mutations at the time of analysis. Resistance mutations (overall and by ART class) detected at failure will be tabulated by number (%) of participants and type of mutation. The proportion of participants with resistance at failure will be compared between arms using the Chi-squared test or Fisher's exact test, as appropriate. 95% confidence interval for the difference in proportion will be provided. Logistic regression will be used for analyses adjusting for stratification factors.

10.12.1.4 Proportion of participants with HIV-1 RNA <50 c/mL and no switch to second-line ART for treatment failure at weeks 24, 48, 72[4] and 96 (cross-sectional analysis)

To evaluate the crude proportion difference between the arms at weeks 24, 48, 72 and 96, Fisher's exact test or Chi-squared test will be used to assess the difference between the SCT and CT arms in the proportion of participants with HIV-1 RNA <50 c/mL and no switch to second-line ART for treatment failure*. 95% confidence interval for the estimated difference in crude proportion of virological rebound at weeks 24, 48, 72 and 96 will be provided using normal approximation. Where appropriate, logistic regression will be used for analyses adjusting for stratification factors. If criterium for multiple imputation has been met (section 9.4.1), multiply imputed HIV-1 RNA data used for primary endpoint analysis (section 10.8) will be used.

*Switch for treatment failure as any change to third agent where reason for change is viral rebound or failure (clinical indication (i.e. what they say on ART log) and follows at least one real-time VL≥50 c/mL). Note that participants may meet the primary endpoint and delay or not switch to second-line ART.

[4]Version 2.0 of the protocol stated that the visit schedule would be 8-weekly visits throughout the trial (week 0, 4 [SCT only], 8, 16, 24, 32, 40, 48, 56, 64, 72, 80, 88, 96 and then every 8 weeks), with a secondary outcome of HIV-RNA <50 c/mL and no switch to second-line ART for treatment failure defined at week 64. However, under protocol version 3.0, visits will be 12-weekly in year 2 (week 0, 4 [SCT only], 8, 16, 24, 32, 40, 48, 60, 72, 84, 96 and then every 12 weeks), where visits will no longer be conducted at week 64.

10.12.2 Safety

10.12.2.1 Change in metabolic parameters from baseline to 96 weeks

Mean changes in lipids, HbA1c and phosphate from baseline will be reported by scheduled visit week and overall, over follow-up. Analysis of the mean change from baseline will be performed as described in section 10.2.

10.12.2.2 Change in renal function from baseline to 96 weeks

Mean changes in eGFR* from baseline will be reported by scheduled visit week and overall, over follow-up. Analysis of the mean change from baseline will be performed as described in section 10.2.

*Calculation of eGFR will be performed using the Cockcroft-Gault formula.

10.12.2.3 Change in anthropometric measures from baseline to 48 and 96 weeks

Mean changes in height, weight, BMI, height-for-age, weight-for-age, BMI-for-age z-scores, waist, hip, waist-hip ratio, from baseline will be reported by scheduled visit week and overall, over follow-up. Analysis of the mean change from baseline will be performed as described in section 10.2.

10.12.2.4 Time to any new or recurrent WHO 3 or WHO 4 events or death

Randomised groups will be compared in terms of time to first event using Cox proportional hazard regression models (adjusted for the stratification factors). Hazard ratios for first events will be presented with a 2-sided 95% confidence interval. Event rates will be calculated as the number of first events/total person years at risk*100 and will be reported per 100 person years including 95% confidence intervals.

10.12.2.5 Incidence of serious adverse events (SAEs)

Serious adverse events will be tabulated separately within the table as follows:

- **Total number of SAEs (number of participants)**
- **Number of SAEs (number of participants) by type of SAE:**
  - Fatal
  - Life-threatening
  - Hospitalisation
  - Persistent or significant disability/incapacity
  - Congenital anomaly/birth defect
  - Important medical condition that carries a real risk of one of the outcomes
- **Number of SAEs (number of participants) by:**
  - System Organ Class (SOC)
  - Preferred Term (PT) within System Organ Class

Hazard ratios, incidence rate ratios and event rates for serious adverse events will be calculated as described previously in section 10.3.
A secondary comparison of the incidence of SAEs between arms will also be made excluding all abortions (spontaneous and induced).

10.12.2.6 Incidence of Grade≥3 clinical and laboratory adverse events (AEs)

Clinical AEs are graded by the clinician reporting the event. Laboratory AEs of Grade≥3 will be graded using the limits defined in Appendix I of the protocol.

Clinical and laboratory AEs will be tabulated separately within the table as follows:

- **Total number of Grade≥3 AEs (number of participants)**

- **Number of Grade≥3 AEs (number of participants) by:**
    - System Organ Class
    - Preferred term within System Organ Class

Hazard ratios, incidence rate ratios and event rates for Grade≥3 AEs will be calculated as described previously in section 10.3.
A secondary comparison of the incidence of Grade≥3 AEs between arms will also be made excluding all abortions (spontaneous and induced).

10.12.2.7 Incidence of adverse events leading to discontinuation or modification of the treatment regimen

Discontinuation or modification of treatment regimen for AE is defined as change of any ART component for AE including dose increase due to use of concomitant medication required for treatment of the AE (e.g. increase in DTG dose due to starting rifampicin for TB treatment). Treatment interruption for >31 days for AE is considered to be discontinuation of treatment regimen for AE. In a secondary analysis, we will exclude AEs which only led to dose modification related to use of concomitant medication (e.g. increase in DTG dose due to starting rifampicin for TB).

AEs (clinical or laboratory) of any grade leading to discontinuation or modification of the treatment regimen will be tabulated as follows:

- **Total number of AEs (number of participants)**
- **Number of AEs (number of participants) by:**
    - System Organ Class
    - Preferred term within System Organ Class.

Hazard ratios, incidence rate ratios and event rates for adverse events leading to discontinuation or modification of the treatment regimen will be calculated as described previously in section 10.3.

10.12.2.8 Proportion with a change in baseline ART regimen

Change in baseline ART regimen will be defined as a change to any component of the baseline regimen (excluding switched between TDF and TAF and 3TC and FTC, and vice versa). Changes in baseline ART regimen during the trial will be tabulated by number (%) of participants. The proportion of participants with changes in baseline ART will be compared between arms using the Chi-squared test or Fisher's exact test, as appropriate. 95% confidence interval for the difference in proportion will be provided. Logistic regression will be used for analyses adjusting for stratification factors.

10.12.2.9 Change in CD4+ and CD8+ count from baseline to weeks 48 and 96

Mean changes in CD4+ and CD8+ from baseline will be reported by scheduled visit week and overall, over follow-up. Analysis of the mean change from baseline will be performed as described in section 10.2.

10.12.3 Patient-reported outcome measures (questionnaires)

10.12.3.1 Adherence, acceptability and wellbeing questionnaires

Descriptive analysis of the adherence and acceptability (HAT-QOL) questionnaires will be undertaken. The proportion of questionnaires where the participant reports missing any dose in the last week (ignoring weekend breaks allowed for in SCT arm) will be compared between randomised groups over time using logistic mixed models, as described in section 10.4.

10.12.3.2 Neuropsychiatric problems (mood survey questionnaire)

Descriptive analysis of the mood survey questionnaire (containing questions on depression, anxiety and sleep disturbance*) will be performed. The proportions reporting symptoms will be compared between randomised groups over time using logistic mixed models or ordered logistic mixed models, as appropriate, as described in section 10.4.

* Three longer questionnaires on depression (PHQ-9), anxiety (GAD-7) and sleep disturbance (based on Pittsburgh sleep questionnaire) will be completed only by participants in the neuropsychiatric toxicity sub-study and will be analysed separately.

10.12.3.3 Suicidal ideation and behaviour

Analysis of suicidal ideation and behaviour data will be performed as per guidance from the "Columbia–Suicide Severity Rating Scale Scoring and Data Analysis Guide" [11]. Analysis will be performed as described in section 10.4.

10.12.3.4 Quality of life (EQ-5D-Y) questionnaires

A descriptive analysis of each dimension of the EQ5D will be undertaken, as described in section 10.4.

10.12.4 Other outcomes

10.12.4.1 Changes in other laboratory measurements from baseline to week 96

Changes in other laboratory measurements from baseline will be reported by scheduled visit week and overall, over follow-up. Analysis of the mean change from baseline will be performed as described in section 10.2.

Analysis will include assessment of changes in:
- Total lymphocyte count (absolute and percentage).
- Haemoglobin, platelets, white cell count, neutrophil and lymphocyte counts, glycosylated haemoglobin A1c. Creatinine, albumin, total bilirubin, alanine transaminase (ALT).

10.12.4.2 MEMS Caps

For participants in the MEMS Cap sub-study, descriptive analyses will report MEMS Cap opening data by day of the week and trial arm.

10.13 TESTING MULTIPLE SECONDARY OUTCOMES

We will make no adjustment to p-values or confidence intervals to allow for testing multiple secondary outcomes. This study has a single primary outcome (confirmed viral rebound by week 96). The primary analysis is non-inferiority of SCT versus CT. If the confidence interval for the treatment effect (difference in proportion experiencing confirmed viral rebound by 96 weeks (SCT-CT)) lies below the selected non-inferiority margin (selected according to the SAFE non-inferiority frontier described in Section 6.1), then we will also test for superiority; because this is a closed test procedure there is no issue of multiplicity.

Secondary outcomes are divided in the protocol into efficacy outcomes, safety outcomes and other patient-reported outcomes. For safety outcomes it is appropriate to test each independently since it is important to identify any risks associated with SCT. The secondary efficacy outcomes are very closely related to the primary outcome. We will not adjust for multiple testing for these since they are correlated with the primary outcome (so standard adjustments are conservative). Resistance and immunological outcomes are considered exploratory and significance tests on these outcomes alone will not be used to conclude superiority.

We will report significance tests for differences between treatment arms for patient-reported outcomes, but if we have failed to demonstrate non-inferiority of SCT versus CT for the primary outcome, we will not use significance tests for these patient-reported outcomes to conclude superiority.

11. SUBSTUDIES

The following sub-studies will be conducted in subsets of participants:

- **i.** Social science sub-study which will quantitatively and qualitatively assess adherence, acceptability and well-being among trial participants.
- **ii.** Neuropsychiatric toxicity sub-study. Specific objectives include: To compare neuropsychiatric toxicities, including depression, suicidality, anxiety and sleep disturbance longitudinally between randomised groups; to test practical and feasible tools to identify and monitor mental health illness among adolescents in busy over-stretched HIV clinics.
- **iii.** Health economics. Specific objectives are to assess the costs and cost-effectiveness of SCT compared to CT.

12. TIMING OF INTERIM ANALYSIS

12.1 PILOT STUDY

Among the first participants randomised into the trial, 15 participants randomised to SCT and 15 randomised to CT at sites participating in the pilot study will have viral load measurements at weeks 1, 2, and 3 (and a confirmatory viral load at week 4 following a single viral load above 50 c/mL at week 3) with these measures in the DTG SCT group being performed after weekends off treatment. Should any participants randomised to SCT in the pilot not take weekends off treatment, additional SCT participants will be recruited. The Independent Data Monitoring Committee (IDMC) will review these HIV RNA assessments after all participants in the pilot phase have completed this pilot. This will be to determine whether the SCT DTG group is safe to continue. Recruitment to the trial will continue during pilot follow-up and review of the pilot data.

12.2 INTERIM ANALYSES

The IDMC will meet to review unblinded data for randomised comparisons within 6 months and 12 months of the trial starting. Batch runs of viral loads will be planned prior to IDMC meetings and should include test results for visits up to 3-4 months prior to the IDMC meeting date; by the 12-month review meeting viral loads 8 and 16 weeks after enrolment should be available on ~140 participants, with 24-week viral loads on ~70 participants. Refer to the IDMC charter for guidance provided on stopping the trial early.

The frequency of future IDMC meetings will be at the discretion of the IDMC but are likely to be every 6-12 months.

13. TIMING OF FINAL ANALYSIS

Analysis of all outcomes will be completed within 6-12 weeks of end of trial defined in protocol: when all participants have attended their final study visit (including follow-up for VL HIV-RNA ≥50 c/mL), retrospective viral load testing is complete, and the database has been locked.

14. APPENDICES

14.1 APPENDIX 1 – HANDLING MISSING DATA WITH MULTIPLE IMPUTATION

This appendix describes the analysis that will be performed if either of the two criteria for multiple imputation listed in section 9.4 of the main statistical analysis plan are met.

14.1.1. GENERAL PROCEDURE

14.1.1.1 Handling missing log_{10}-transformed viral load measurements at baseline

Missing viral load (VL) data at baseline are expected to be minimal, and participants should be virologically suppressed at baseline. If there are missing log_{10}-transformed VL measurements at baseline, the missing values will be replaced with the overall mean of the observed log_{10}-transformed VL measurements. Mean imputation has been shown to be a valid approach for handling missing data in baseline covariates[12]. Baseline VL measurements known to be below the limit of detection (i.e. left-censored) will be handled using the procedure described in section 14.1.2. Note that baseline VL data do not contribute to the derivation of the primary endpoint and are therefore only used to inform imputation of follow-up VL data.

14.1.1.2 Imputing log_{10}-transformed viral load measurements post baseline

Multiple imputation of log_{10}-transformed VL measurements at 12 scheduled visits post-baseline (weeks 8, 16, 24, 32, 40, 48, 60, 72, 84, 96, 108, and 120) will be performed by multivariate imputation by chained equations (via mi impute chained in Stata)[13], based on the assumption of data being missing at random[14]. Week 120 visit window will be included in the imputation as an auxiliary variable (i.e. to inform imputation of week 108 visit window, and not to derive the primary endpoint), provided that there are at least 50% of participants with a VL measurement in that window.

Imputation will be performed separately in each randomised arm (via mi impute chained, by() in Stata) to allow for any interactions with randomised arms.

One log_{10}-transformed VL measurement per visit window will be included in the imputation model as a separate continuous variable (i.e. 12 incomplete variables to be imputed). For visit windows with more than 1 VL measurement, the choice of which measurement to include is described below.

Information on treatment changes will be summarised in the following two variables which will then be used to inform imputation of missing VL data:

- Before imputation, an overall binary indicator of whether each participant made a change to their originally randomised treatment (excluding changes between TDF and TAF or 3TC and FTC) or strategy (SCT to CT) (at any time after randomisation and before week 120), will be created; this indicator of treatment change takes value 1 if the participant made a treatment change, and 0 if the participant did not make a treatment change;

- Another variable containing time to treatment change will also be created. For participants who made a treatment change (i.e. for whom the binary indicator of treatment change takes value 1), this variable is defined as time (in weeks) from randomisation to the date of the treatment change. For participants who did not make a treatment change, this variable is replaced with time (in weeks) from randomisation to the scheduled visit date of their 120-week visit.

Each missing VL measurement per visit window will be imputed using predictive mean matching, using 11 nearest neighbours (via mi impute chained(pmm, knn(11))in Stata), conditional on the following:

- observed and imputed VL measurements from all other post-baseline visit windows;
- observed and mean imputed baseline log_{10}-transformed VL data;
- an overall binary indicator of whether the participants made a change to their originally randomised treatment (as defined above);

- a variable containing the time from randomisation to treatment change (as defined above); and
- site.

If the imputation procedure fails to converge in either arm (in more than 5% of any of the bootstrapped datasets, see section 14.1.3), the imputation models will be simplified for both arms (and for all bootstrapped datasets, see section 14.1.3), where each missing VL measurement per visit window will be imputed conditional on the following:

- observed and imputed VL measurements from ± 1 adjacent window (e.g. VL data at week 48 will be imputed using VL data at weeks 40 and 60, via the option omit() in mi impute chained in Stata);
- observed and mean imputed baseline VL data;
- an overall binary indicator of whether the participants made a change to their originally randomised treatment (as defined above);
- a variable containing the time from randomisation to treatment change (as defined above); and
- site.

The multivariate imputation by chained equations algorithm is an iterative procedure that cycles through the incomplete variables to be imputed a number of times before an imputed dataset is created, in order to ensure convergence of the conditional imputation models. Previous exploratory multiple imputation work using data from the ODYSSEY trial suggests that the number of cycles of the chained equations needs to be increased from the default of 10 to 30.

The choice of the number of imputations to be performed will be discussed in section 14.1.3.

14.1.1.2.1 SENSITIVITY ANALYSIS 1*

The multivariate imputation by chained equations procedure described in section 14.1.1.2 uses predictive mean matching to perform the imputation. Predictive mean matching uses normal linear regression to obtain linear predictions which are then used to match the missing values with the nearest neighbours. Since $\log_{10}$-transformed VL data are not likely to be normally distributed, the normality assumption of this model used by predictive mean matching might be violated. However, by drawing from the observed data to impute the missing data, predictive mean matching preserves the distribution of the observed VL measurements in the imputed data, which makes it more robust than using a linear regression imputation approach.

To explore sensitivity to violation of the normality assumption, a sensitivity analysis will be performed where, instead of imputing the $\log_{10}$-transformed VL measurement at each visit window, the indicator of suppression at each visit window (taking value 0 if HIV-1 RNA <50c/mL, 1 if HIV-1 RNA ≥50c/mL) is imputed. Left-censored VL measurements will be handled as per the procedure described in section 14.1.2, before the values of their associated indicators of suppression are derived.

One indicator of suppression per window will be included in the imputation model as a separate binary variable. For visit windows with more than 1 VL measurement, the choice of which measurement to include is described in section 14.1.1.3.

Imputation will be performed separately in each randomised arm (via mi impute chained, by() in Stata) to allow for any interactions with randomised arms.

Each missing indicator of suppression will be imputed using a logistic regression conditional imputation model (via mi impute chained (logit) in Stata), conditional on the following:

- observed and imputed indicators of suppression from all other visit windows;
- observed and mean imputed baseline $\log_{10}$-transformed VL data;
- an overall binary indicator of whether the participants made a change to their originally randomised treatment (as defined above);
- a variable containing the time from randomisation to treatment change (as defined above); and
- site.

This approach does not rely on the normality assumption, however it uses less information than modelling the distribution of $\log_{10}$-transformed VL data, and is prone to perfect prediction. In case of perfect prediction, an augmentation procedure will be performed where the dataset is augmented with a few extra observations to avert

perfect prediction, which are then assigned some small weights to limit their impact on the estimated imputation model[15]. This procedure has been implemented in the mi impute suite in Stata, via the option augment that is available with the logistic regression conditional imputation model. In case data augmentation cannot overcome perfect prediction in either arm (in more than 5% of the bootstrapped datasets, see section 14.1.3), the conditional imputation models will be simplified for both arms (and for all bootstrapped datasets, see section 14.1.3), so that the missing indicator of suppression in each visit window is imputed conditional on the following:

- the observed and imputed indicators of suppression from ± 1 adjacent window (via the option omit()in mi impute chained in Stata, e.g. indicator of suppression at week 48 will be imputed using indicators of suppression at weeks 40 and 60);
- observed and mean imputed baseline log_{10}-transformed VL data;
- an overall binary indicator of whether the participants made a change to their originally randomised treatment (as defined above);
- a variable containing the time from randomisation to treatment change (as defined above); and
- site.

14.1.1.3 Handling visit windows with more than 1 viral load measurement

For visit windows with more than 1 VL measurement, the measurement from the undiluted sample with date of collection closest to the scheduled visit date of that window (or if there are no undiluted samples, the diluted sample with closest date) will be selected for that window to be used in the imputation model. Where there are two values (meeting the same criteria) within a window, both equidistant from the nominal visit week, the later value will be used. Once missing VL measurements have been imputed for all scheduled visit windows, i.e. all windows have 1 measurement, the imputed data will be appended back to the originally observed data (for each of the B bootstrapped datasets, see section 14.1.3) in which some windows have multiple VL measurements. In this appended dataset, all log_{10}-transformed VL data will be back transformed to the original scale. The analysis of the primary endpoint will be performed on this appended dataset so that all available observed VL measurements as well as imputed VL measurements are used to derive the primary endpoint (the first of 2 consecutive HIV-1 RNA ≥50c/mL).

14.1.1.3.1 SENSITIVITY ANALYSIS 2*

A sensitivity analysis will be performed where the last undiluted log_{10}-transformed VL measurement of windows with multiple measurements will be selected for inclusion in the imputation model.

14.1.1.3.2 SENSITIVITY ANALYSIS 3*

Repeats following initial real-time HIV-1 RNA ≥50c/mL are mandated at all sites for weeks 48, and 96. A sensitivity analysis will be performed where, for each of weeks 48, and 96, 1 main log_{10}-transformed VL measurement and 1 repeat log_{10}-transformed VL measurement (if the initial VL is raised) will be selected, and missing repeats will be imputed as separate variables alongside VL measurements at all visit windows post-baseline as part of the *same* multiple imputation procedure described in sections 14.1.1.2 and 14.1.1.3 (i.e. 12 incomplete main VL variables and 2 incomplete repeat VL variables to be imputed together in one multivariable imputation by chained equations procedure).

Conditional imputation (via mi impute chained (pmm, cond()) in Stata) will be performed for the repeats, such that a missing repeat will only be imputed if the observed or imputed initial VL in the same visit window is raised. Each repeat variable will be imputed conditional on the following:

- the initial VL measurement in that window;
- the main measurement in the next window, e.g. missing repeat at week 48 will be imputed conditional on the initial VL in week 48 and VL in week 60 (via the option omit() in mi impute chained in Stata);
- observed and mean imputed log_{10}-transformed VL data at baseline;
- an overall binary indicator of whether the participants made a change to their originally randomised treatment (as defined above);
- a variable containing the time from randomisation to treatment change (as defined above); and

- site.

Repeats (i.e. the 2 repeat variables) are all excluded from the imputation model of missing VL measurements in all scheduled visit windows (i.e. the 2 repeats variables are not used to inform the imputation of the 12 main variables, via the option omit() in mi impute chained in Stata). Imputation will be performed separately in each randomised arm.

**Note: If 2 of the 3 sensitivity analyses described in sections 14.1.1.2.1, 14.1.1.3.1, 14.1.1.3.2 indicate sensitivity, they will be combined into a further sensitivity analysis.*

14.1.2. HANDLING VIRAL LOAD MEASUREMENT BELOW THE LIMIT OF DETECTION

In BREATHER Plus, samples are run on machines with highest LLOD = 50c/mL (e.g. LLOD = 20c/mL, 40c/mL, etc).

- If a sample is undiluted, the upper bound of the resulting VL measurement is equal to the LLOD (e.g. 20c/mL, 40 c/mL, 50c/mL);

- If a sample is diluted, the upper bound of the resulting VL measurement is equal to these values multiplied by the dilution ratio (1:2, 1:3, ..., 1:5), so the highest possible upper bound is 250c/mL.

A cut-off of <50c/mL is used to define suppression. Each sample either results in a VL reading ('x') or is known to be below the LLOD ('<x'), and the latter occurs with x = LLOD (e.g. <20c/mL, <40 c/mL, <50c/mL), or multiples of these limits when a dilution is made. This complicates both the primary analysis and the imputation of $\log_{10}$-transformed VL because for some observed VL measurements we only know the upper bound and not the actual values (i.e. VL data are left-censored). Suppression status (<50c/mL) is clear, except when the sample is known to be <LLOD *and* the LLOD is above 50c/mL due to dilution (see 3. below).

Below are some examples of potential scenarios given the 50c/mL cut-off for suppression:

Scenario 1 – Suppressed (<50c/mL):

a. Undiluted: observed HIV-1 RNA <20c/mL, <40c/mL, <50c/mL; VL measurement is left-censored;
b. Undiluted: observed HIV-1 RNA = 27c/mL, run on a machine with LLOD = 20c/mL; VL measurement is known;
c. Diluted: adjusted (for dilution) HIV-1 RNA <40c/mL, from a 1:2 diluted sample run on a machine with LLOD = 20 c/mL (so compared with adjusted upper bound = 40 c/mL); VL measurement is left-censored;
d. Diluted: adjusted HIV-1 RNA = 46c/mL, from 1:2 diluted sample run on a machine with LLOD = 20c/mL (so compared with adjusted upper bound = 40 c/mL); VL measurement is known;

Scenario 2 – Unsuppressed (≥50c/mL):

e. Undiluted: observed HIV-1 RNA = 105c/mL; VL measurement is known;
f. Diluted: e.g. adjusted HIV-1 RNA = 106c/mL, from a 1:2 diluted sample run on a machine with LLOD = 50c/mL (so compared with adjusted upper bound = 100c/mL); VL measurement is known;

Scenario 3 – Unclear:

g. Diluted: e.g. adjusted HIV-1 RNA <100c/mL, from a 1:2 diluted sample run on a machine with LLOD = 50c/mL (so compared with adjusted upper bound = 100c/mL); here we know that HIV-1 RNA is <100c/mL but we cannot determine if HIV-1 RNA is also <50c/mL or 50c/mL ≤ HIV-1 RNA < 100c/mL; VL measurement is left-censored.

The following procedure will be followed to deal with left-censored data.

Using all available undiluted samples at all visit windows considered in the analysis of the primary endpoint, we will calculate the percentage of participants who are virologically suppressed (HIV-1 RNA <50c/mL) among all participants whose HIV-1 RNA is known to be (i) <100/c/mL; and (ii) <200c/mL. The 200c/mL bound is chosen instead of the highest possible 250c/mL bound (for machines with LLOD = 50c/mL and samples diluted 1:5) since it is likely to occur more frequently in the dataset.

- If both percentages (i) and (ii) are ≥ 80%, all left-censored VL measurements (as described above) will be replaced with 25c/mL, based on the assumption that if the majority of participants whose VL measurements are <100c/mL and <200c/mL are in fact virologically suppressed then it is reasonable to replace with a measurement that reflects suppression;

- If percentage (i) is ≥ 80% while percentage (ii) is <80%, left censored VL measurements known to be either <50c/mL or <100c/mL will be replaced with 25c/mL; left censored VL measurements known to be <200c/mL (excluding those known to be <100c/mL) will be replaced with 100c/mL.

- If percentage (i) is <80% while percentage (ii) is ≥ 80% , or if both percentages (i) and (ii) are <80%, left-censored VL measurements known to be <50c/mL will be replaced with 25c/mL; left-censored VL measurements known to be <100c/mL (excluding those known to be <50c/mL) will be replaced with 50c/mL; and left-censored VL measurements known to be <200c/mL (excluding those known to be <100c/mL) will be replaced with 100c/mL.

14.1.3. COMBINING BOOTSTRAPPING WITH MULTIPLE IMPUTATION

Since bootstrapping will be used to estimate standard errors and confidence intervals in the primary analysis, multiple imputation needs to be performed in a way that allows for bootstrapping. We will use a procedure recommended by Bartlett & Hughes (2020) involving bootstrapping followed by imputation (termed Boot-MI)[16], where:

- B bootstrapped datasets are created from bootstrapping the incomplete dataset. Bootstrapped samples are selected within strata defined by randomised arms and stratification variables;

- Each bootstrapped dataset is imputed M times using the procedure described in sections 14.1.1 and 14.1.2, resulting in B×M bootstrapped imputed datasets;

- The substantive analysis is performed in each of these B×M datasets, producing B×M point estimates (adjusted difference in proportions);

- The pooled point estimate for inference is the average of these B×M estimates; a one-way random effects ANOVA model is fitted to these B×M point estimates and its results used to estimate the associated standard error; 95% confidence interval is based on a *t*-distribution.**

***Note: it is not possible to obtain bias-corrected bootstrap confidence intervals with Boot-MI, since the confidence interval is based on a t-distribution.*

Bartlett & Hughes (2020) showed that using a large number of bootstrapped datasets (e.g. B=1000) and a small number of imputations (M=2) is computationally efficient[16]. For BREATHER Plus, B=10,000 bootstrapped datasets and M=5 imputations will be created, resulting in 50,000 bootstrapped imputed datasets that will be analysed according to the Boot-MI procedure described above.

14.1.4. USING IMPUTED MISSING VIRAL LOAD MEASUREMENTS AFTER LOSS TO FOLLOW-UP AND DEATH

Loss to follow-up and death are not components of the primary endpoint in BREATHER Plus (proportion of children with confirmed viral rebound, defined as the first two consecutive HIV-1 RNA ≥50c/mL, by week 96).

Multiple imputation will be performed for all visit windows as described above. Then for participants who were lost to follow-up, withdrew, or died prior to meeting the primary endpoint, if their last observed or imputed VL measurement is raised (HIV-1 RNA ≥50c/mL) prior to loss to follow-up, withdrawal, or death, this measurement as well as the VL measurement imputed in the next visit window will be used to derive the primary endpoint or to determine the censoring time. All subsequent imputed VL measurements for these participants are not used for this purpose and will be deleted. The procedure is summarised in Appendix Table A1.

The imputation procedure described in section 14.1.1 does not include as covariates indicators of loss to follow-up, withdrawal, and death. Since none of these events is a component of the primary endpoint (proportion of children with confirmed viral rebound, defined as the first two consecutive HIV-1 RNA ≥50c/mL, by week 96), omitting these variables from the imputation model will likely not bias the results.

Appendix Table A1. Procedure for using VL measurement observed before loss to follow-up/death and imputed VL measurement at the following visit window to derive the primary endpoint or determine the censoring time.

**Observed or imputed VL at week *w* visit window**		**Imputed VL at week *w+12*ǂ visit window**	**Status**
≥50c/mL (with no confirmation)	The participant was lost to follow-up, withdrew, or died between week *w* and *w+12*ǂ	≥50c/mL	The participant met the endpoint at week *w*
≥50c/mL (with no confirmation)		<50c/mL	The participant did not meet the endpoint by week *w+12*ǂ, censored at week *w+12*ǂ
<50c/mL		≥50c/mL	The participant did not meet the endpoint by week *w*, censored at week *w*
<50c/mL		<50c/mL	The participant did not meet the endpoint by week *w*, censored at week *w*

ǂor *w+8* if *w≤40*.

Note that the methods described in Appendix 1 were written (initially for the D3 trial) by Dr Tra My Pham with support from Professor Ian White without reference to accumulating unblinded data. Dr Man Chan and Dr Debbie Ford (who are unblinded to accumulating data) reviewed the methods but did not provide information on D3 or BREATHER Plus data.

14.2 APPENDIX 2 - PROPOSED RULES FOR REQUESTING RETROSPECTIVE HIV-1 RNA TESTING FROM STORED PLASMA SAMPLES

To avoid requesting retrospective samples for a given visit, which already have real time HIV-1 RNA result available, the following rules will be used:

Appendix table A2. Proposed rules for requesting retrospective HIV-1 RNA testing from stored plasma samples

**Real time HIV-1 RNA**	**Retrospective HIV-1 RNA**<br>(Stored plasma sample)
**Available**<br>In database for a visit week window	**DO NOT REQUEST stored sample testing**<br>(unless dilution has been used and result cannot be classified as < or >=50 c/mL)<br>[in visit week window]
**Not available**<br>In database for a visit week window	
**Pending data entry**<br>Checked and confirmed by site real time HIV-1 RNA sample taken at visit; pending entry	**DO NOT REQUEST stored sample testing**<br>[in visit week window]
**Confirmed missing HIV-1 RNA**<br>Checked and confirmed by site real time HIV-1 RNA sample is not expected (e.g. due to missed visit, sample lost, failed test ,etc)	**REQUEST stored sample testing**<br>[in visit week window]

Regularly extract HIV-1 RNA data from database and keep track of availability of real time HIV-1 RNA data.

14.3 APPENDIX 3 - CONSIDERATIONS/ASSUMPTIONS ON HIV-1 RNA DATA FOR ANALYSIS

Real time and retrospective HIV-1 RNA

Real time HIV-1 RNA will be used for analysis where available at each study visit (mandatory at weeks 48, 96 and 48 weekly thereafter and end of trial visit). When a real time result is not available (including if missing at a mandatory visit or confirmatory timepoint), a retrospective HIV-1 RNA will be requested and used for the analysis. Similarly, if for a real-time result, the HIV-1 RNA Lower Limit of Detection (LLD) is >50 c/mL (for example, when a HIV-1 RNA test is conducted on a diluted sample or dried blood spot) a retrospective HIV-1 RNA test will be requested.

Where a participant has a real time and retrospective HIV-1 RNA within the same visit week window, the real time HIV-1 RNA will be used as the retrospective result should not have been requested. The exception to this is when the real time HIV-1 RNA Lower Limit of Detection (LLD) is >50 c/mL (as above), in which case, the retrospective sample will be used instead.

Diluted samples

Sites/labs must clearly indicate the dilution factor used if samples were diluted for HIV-1 RNA measurement.

HIV-1 RNA measured in diluted samples may be provided in two forms depending on site:

1) **Diluted HIV-1 RNA result (unadjusted for dilution factor; dilution factor not automatically adjusted for by the assay instrument):** these results will be adjusted for dilution factor when creating analysis datasets by multiplying the result by the dilution factor to obtain the “useable” result i.e. result adjusted for dilution. This “useable” result will be used in the analysis.

2) **Undiluted HIV-1 RNA result (adjusted for dilution factor; dilution factor already automatically adjusted for by the assay instrument**): these results are already in “useable” form and will be used in the analysis as provided.

For the analysis of primary endpoint (confirmed HIV-1 RNA ≥50 c/mL), the following considerations will be implemented for grouping of HIV-1 RNA results into <50 c/mL and ≥50 c/mL groups using the “useable” HIV-1 RNA results:

1) **“useable” HIV-1 RNA result with value:**
    - **a.** equal to or greater than (“≥”) 50 c/mL and sign “=” will be allocated to “≥50 c/mL” group (e.g. HIV-1 RNA=80 participant will be allocated to ≥50 c/mL);
    - **b.** less than (“<”) 50 c/mL and sign “=” or “<” will be allocated to “<50 c/mL” group;
    - **c.** equal to (“=”) 50 c/mL and sign “<” will be allocated to “<50 c/mL” group;

2) “useable” HIV-1 RNA result with value greater than (“>”) 50 c/mL and sign “<”, for example, <80, <100 and <200, will not be used for grouping and will be assumed to be missing.

3) If “useable” HIV-1 RNA result is missing due to target not detected, it will be replaced by the LLD with a sign “<”. For example, the LLD=40 c/mL, HIV-1 RNA = “target not detected” and sign is N/A, we will replace “target not detected” by 40 and assign sign “<”. Once replaced, grouping will be done as described above in points 1) and 2).

14.4 APPENDIX 4 - DETAILS OF MODIFIED FDA SNAPSHOT ALGORITHM

This section outlines the modified version of the FDA snapshot algorithm, which will be used to compare virological rebound (HIV-1 RNA ≥50 c/mL) in SCT vs. CT at weeks 48 and 96.

Since the BREATHER Plus protocol specifies the following drugs are interchangeable, switches from 3TC to FTC and TDF to TAF (or vice versa) will be ignored in the algorithm. Switch for lack of efficacy will always be treated as a non-permitted change, however, when a participant switches from 3TC to FTC and TDF to TAF (or vice versa) for this reason, they will likely switch another component of their regimen at the same time.

Non-permitted changes in SCT arm include:

- Switch from SCT to continuous therapy for any reason (excluding switch for protocol deviation or patient/carer decision <7 days [permitted change, see below]).

Non-permitted changes in both arms include:

- Change of ART component(s) due to lack of efficacy
- Change of ART component(s) due to adverse event*
- Change of ART component(s) due to pregnancy or desire to become pregnant*
- Change of ART component(s) due to protocol deviation or patient/carer decision where the time off the allocated regimen is ≥7 days*

*Except for changes from 3TC to FTC and TDF to TAF (or vice versa) which are ignored

Permitted ART changes in both arms include:

- Change of ART component(s) or strategy for incorrect prescribing (protocol deviation) or patient/carer decision provided that the participant switches back to their allocated regimen and strategy <7 days after change*

*Except for changes from 3TC to FTC and TDF to TAF (or vice versa) which are ignored

Ignored ART changes in both arms include changes where components of the ART regimen remain unchanged (and in the SCT arm, the strategy remains unchanged) unless otherwise specified:

- Dose change of 3TC for creatinine clearance
- Move to double DTG dose (or adjustment to TAF dose) for TB treatment and return to single dose following completion of TB treatment or similar dose adjustments due to drug-drug interactions with other concomitant medications (applicable in CT arm only)
- Switch to fixed dose ART combination (FDC) (or FDC to single/dual) containing the same ART components
- Changes between different products containing the same ART components
- Changes between mornings and evenings
- Switch from 3TC to FTC, or vice versa, for any reason other than lack of efficacy
- Switch from TDF to TAF, or vice versa, for any reason other than lack of efficacy

Classification (ignored/permitted/non-permitted) of ART changes for any other reason will be reviewed by a clinician blinded to trial arm and with no access to viral loads. ART switches for breastfeeding (not directly captured on ART log but via "Other" code) will be classified according to rules used for pregnancy.

ART interruptions/stops

Participants who have not been seen since baseline and have no HIV-1 RNA data on ART post-baseline will not be categorised for the purposes of the FDA snapshot algorithm and will therefore be excluded from the analysis.

Only post-baseline and HIV-1 RNA on-ART will be used in the algorithm. Stop of any ART component is considered an ART interruption. Participants interrupting ART prior to or within week 48/96 window are not classified as treatment switch until a new treatment has started (rules above will be applied). If they resume the same treatment, the period of the ART interruption is ignored although HIV-1 RNA off ART will not be used.

Where a participant has <7 days on an incorrect/new regimen/strategy for protocol deviation or patient/carer decision before returning to their allocated regimen/strategy, HIV-1 RNA data will not be used in the algorithm until the participant has been back on their allocated regimen and strategy for ≥7 days. HIV-1 RNA data post change are not used in the algorithm for participants who have ≥7 days on an incorrect/new regimen/strategy for protocol deviation or patient/carer decision.

Multiple HIV-RNA results within a window
In the case where a participant has multiple HIV-1 RNA results within week 48/96 window, the latest HIV-1 RNA should be used in the FDA snapshot algorithm.

Secondary analysis
Given the trial protocol stipulates SCT participants must switch to continuous therapy when becoming pregnant, receiving treatment for TB (or other dose adjustments due to drug-drug interactions with other concomitant medications), a secondary analysis will treat switches from SCT to CT due to pregnancy/breastfeeding or TB treatment/other concomitant medications as a permitted switch.

Appendix table A3. Full algorithm for modified FDA snapshot analysis of virological rebound

**1. Non-permitted change in therapy prior to week 48/96**		
**1a.** Last on-treatment HIV-1 RNA at/prior to change ≥50 c/mL (or change for lack of efficacy*)	HIV-1 RNA≥50 c/mL	Change in therapy/strategy while HIV-1 RNA ≥50 c/mL
**1b.** Last on-treatment HIV-1 RNA at/prior to change <50 c/mL or no on-treatment HIV-1 RNA available during study	No virologic data in week 48/96 window	Change in therapy/strategy while HIV-1 RNA <50 c/mL
**2. Permitted change in therapy prior to week 48/96 and last HIV-1 RNA on treatment at/prior to change ≥50 c/mL**	HIV-1 RNA≥50 c/mL	Change in therapy/strategy while HIV-1 RNA ≥50 c/mL
**3. Non-permitted change in therapy during week 48/96**		
**3a.** Last on-treatment HIV-1 RNA during week 48/96 prior to/on the date of change ≥50 c/mL	HIV-1 RNA≥50 c/mL	Data in window not below 50 c/mL
**3b.** Last on-treatment HIV-1 RNA during week 48/96 prior to/on the date of change <50 c/mL	HIV-1 RNA<50 c/mL	Data in window below 50 c/mL
**3c. No HIV-1 RNA during week 48/96 prior to/on the date of change**		
**3c (i).** Last on-treatment HIV-1 RNA at/prior to change ≥50 c/mL (or change for lack of efficacy)	HIV-1 RNA≥50 c/mL	Change in therapy/strategy while HIV-1 RNA≥50 c/mL
**3c (ii).** Last on-treatment HIV-1 RNA at/prior to change <50 c/mL or no on-treatment HIV-1 RNA available during study	No virologic data in week 48/96 window	Change in therapy/strategy while HIV-1 RNA<50 c/mL
**4. Permitted change in therapy during week 48/96 and last on treatment HIV-1 RNA at/prior to change ≥50 c/mL**		
**4a.** Last on-treatment HIV-1 RNA is during week 48/96 and prior to/on date of change	HIV-1 RNA≥50 c/mL	Data in window not below 50 c/mL (see 3a)
**4b.** Last on-treatment HIV-1 RNA is prior to week 48/96	HIV-1 RNA≥50 c/mL	Change in therapy/strategy while HIV-1 RNA≥50 c/mL (see 3c)
**If none of above**		

**5. HIV-1 RNA available on allocated regimen/strategy (with prior permitted changes allowed while HIV-1 RNA<50 c/mL) in 48/96 week window**		
**5a.** Last on-treatment HIV-1 RNA during week 48/96 ≥50 c/mL (assuming anyone with HIV-1 RNA≥50 c/mL at week 48/96 will be tested by week 54/102**)	HIV-1 RNA≥50 c/mL	Data in window not below 50 c/mL
**5b.** Last on-treatment HIV-1 RNA during week 48/96 <50 c/mL (assuming anyone with HIV-1 RNA≥50 c/mL at week 48/96 will be tested by week 54/102**)	HIV-1 RNA<50 c/mL	Data in window below 50 c/mL
**6. Participant is on allocated regimen/strategy (including permitted changes while HIV1-RNA<50 c/mL) but has no HIV-1 RNA data in 48/96 week window**	No virologic data in week 48/96 window	Missing data in window
**7. Participant died/lost to follow-up/withdrew prior to 48/96 week window and last on-treatment viral load <50 c/mL or no on-treatment HIV-1 RNA available during study**	No virologic data in week 48/96 window	Death, LTFU, withdrawal from trial while HIV1-RNA<50 c/mL
**8. Participant died/lost to follow-up/withdrew prior to 48/96 week window and last on-treatment viral load ≥50 c/mL**	HIV-1 RNA≥50 c/mL	Death, LTFU, withdrawal from trial while HIV-1 RNA≥50 c/mL

* Sites will be asked to do a viral load test before changing treatment for suspected treatment failure; however, if there is a treatment change for clinical or immunological failure, this will be included here irrespective of most recent viral load
**Every effort will be made to do confirmatory viral loads within the visit window. If this is not done for any reason, then the participant will be classified under 5a and not 5b (i.e. as HIV-1 RNA≥50 c/mL).
Ignored changes do not affect participant's classification.
Any viral loads which due to dilution cannot be classified as </≥50 c/mL will not be used.

Appendix table A4. Categorisation of Virological Outcomes at 48/96 weeks (within window 42-54/90-102 weeks)

Ignored, permitted, and non-permitted ART changes are specified above. If participant has changed regimen/strategy in any way in either arm (including loss to follow-up/withdrawal or death) before/during week 48/96 then **(i)** if last HIV-1 RNA prior to change is ≥50 c/mL, they are a rebound, **(ii)** if last HIV-1 RNA <50 c/mL they will have no virologic data unless they made a permitted change in which case 48/96 week data can be used as if they are continuing as randomised.

	**SCT**	**CT**
	N (%)	N (%)
**HIV-1 RNA≥50 c/mL[1]**		
**Treatment difference (95% CI)**		
**HIV-1 RNA<50 c/mL[2]**		
**No virological data in week 48/96 window**		
Discontinued allocated regimen/strategy due to AE or death and last on treatment (at/prior to change) HIV-1 RNA<50 c/mL[3]		
Discontinued allocated regimen/strategy for other reasons and last on treatment (at/prior to change) HIV-1 RNA<50 c/mL[4]		
On study allocated regimen and strategy but missing HIV-1 RNA data in window[5]		

[1] Includes: **(i)** participants on allocated regimen and strategy (SCT/CT), including those with prior permitted changes while HIV-1 RNA<50 c/mL, who had confirmed HIV-1 RNA≥50 c/mL in 48/96 week window; **(ii)** participants who changed any component of allocated regimen or resumed continuous therapy in the SCT arm because of lack of efficacy prior to/during week 48/96 window; **(iii)** participants who discontinued/changed any component of allocated regimen or resumed continuous therapy in the SCT arm for reasons other than lack of efficacy prior to/during week 48/96 with the last on treatment (prior to/on the date of change) HIV-1 RNA ≥50 c/mL.

[2] Includes: **(i)** participants on allocated regimen and strategy (SCT/CT), including those with prior permitted changes while HIV-RNA<50 c/mL, with HIV-1 RNA<50 c/mL in week 48/96 window.

[3] Includes participants who discontinued or changed any component of allocated regimen or strategy for AE or death before or during week 48/96 where last on treatment HIV-RNA<50 c/mL.

[4] Includes participants who discontinued or changed any component of allocated regimen or strategy for reasons other than an AE or death or lack of efficacy, e.g., withdrew consent, lost to follow-up, pregnancy (or desire to become pregnant), transferred care to a non-study site, patient/carer decision or protocol deviation that do not meet the criteria for permitted change, or had any other non-permitted change before or during week 48/96 where last on treatment HIV-1 RNA<50 c/mL.

[5]Includes participants remaining on allocated regimen and strategy (SCT/CT), including those with prior permitted changes while HIV-1 RNA<50 c/mL, who had no available HIV-1 RNA in week 48/96 window.

Ignored changes do not affect participant's classification.
Any viral loads which due to dilution cannot be classified as </≥50 c/mL will not be used.

REFERENCES

1. Quartagno, M., et al., *Handling an uncertain control group event risk in non-inferiority trials: non-inferiority frontiers and the power-stabilising transformation.* Trials, 2020. **21**(1): p. 145.
2. Matilde Sanchez, M. and X. Chen, *Choosing the analysis population in non-inferiority studies: per protocol or intent-to-treat.* Stat Med, 2006. **25**(7): p. 1169-81.
3. Sheng, D. and M.Y. Kim, *The effects of non-compliance on intent-to-treat analysis of equivalence trials.* Stat Med, 2006. **25**(7): p. 1183-99.
4. Wiens, B.L. and W. Zhao, *The role of intention to treat in analysis of noninferiority studies.* Clin Trials, 2007. **4**(3): p. 286-91.
5. World Health Organisation, *WHO case definitions of HIV for surveillance and revised clinical staging and immunological classification of HIV-related disease in adults and children* (http://www.who.int/hiv/pub/guidelines/HIVstaging150307.pdf). 2007.
6. *U.S. Department of Health and Human Services, National Institutes of Health, National Institute of Allergy and Infectious Diseases, Division of AIDS. Division of AIDS (DAIDS) Table for Grading the Severity of Adult and Pediatric Adverse Events, Corrected Version 2.1. [July 2017]. Available from: https://rsc.niaid.nih.gov/sites/default/files/daidsgradingcorrectedv21.pdf.*
7. WHO, *Antiretroviral therapy for HIV infection in infants and children: Towards universal access. Recommendations for a public health approach: 2010 revision* Vol. 13 July 2010. 2010, Geneva.
8. Morris, T.P., et al., *Planning a method for covariate adjustment in individually-randomised trials: a practical guide. arXiv:2107.06398.* https://arxiv.org/, 2021.
9. Royston, P. and M.K. Parmar, *Flexible parametric proportional-hazards and proportional-odds models for censored survival data, with application to prognostic modelling and estimation of treatment effects.* Statistics in medicine, 2002. **21**(15): p. 2175-2197.
10. *Food Drug Administration Center for Drugs Evaluation Research. Human Immunodeficiency Virus-1 Infection: Developing Antiretroviral Drugs for Treatment - Guidance for Industry.* 2015: Maryland, USA: FDA.
11. Mary E. Nilsson, et al., *https://cssrs.columbia.edu/wp-content/uploads/ScoringandDataAnalysisGuide-for-Clinical-Trials-1.pdf*. 2013.
12. White, I.R. and S.G. Thompson, *Adjusting for partially missing baseline measurements in randomized trials.* Statistics in medicine, 2005. **24**(7): p. 993-1007.
13. Van Buuren, S., H.C. Boshuizen, and D.L. Knook, *Multiple imputation of missing blood pressure covariates in survival analysis.* Statistics in medicine, 1999. **18**(6): p. 681-694.
14. Rubin, D.B., *Multiple imputation for nonresponse in surveys Donald B. Rubin*. Wiley classics library. 2004, Hoboken, N.J. ;: Wiley-Interscience.
15. White, I.R., R. Daniel, and P. Royston, *Avoiding bias due to perfect prediction in multiple imputation of incomplete categorical variables.* Computational statistics & data analysis, 2010. **54**(10): p. 2267-2275.
16. Bartlett, J.W. and R.A. Hughes, *Bootstrap inference for multiple imputation under uncongeniality and misspecification.* Statistical methods in medical research, 2020. **29**(12): p. 3533-3546.
